# Pathogen-inspired engineering of plant protease enhances late blight resistance

**DOI:** 10.1073/pnas.2524700123

**Published:** 2026-01-09

**Authors:** Jie Huang, Alice Penrose, Laura Ossorio Carballo, Renier A. L. van der Hoorn

**Affiliations:** ^a^The Plant Chemetics Laboratory, Department of Biology, University of Oxford, Oxford OX1 3RB, United Kingdom

**Keywords:** apoplast, plant immunity, protease, inhibitor, *Phytophthora infestans*

## Abstract

The apoplast is an important battlefield in plant–pathogen interactions. The late blight oomycete pathogen *Phytophthora infestans,* for instance, secretes cystatin-like protease inhibitors EpiC1 and EpiC2B to suppress C14, a papain-like immune protease secreted by tomato. Here, we found that *P. infestans* also secretes two distinct papain-like proteases termed Pain1 and Pain2, which are transcriptionally induced during infection. Both Pains promote *P. infestans* infection, but not when their catalytic residues are mutated. Strikingly, EpiC1 and EpiC2B preferentially inhibit tomato C14 rather than self-produced Pains, suggesting that they coevolved with Pains to avoid self-inhibition. To mimic the avoidance of inhibition by EpiCs, we engineered C14 (eC14) with seven Pain1 residues that potentially disturb the EpiCs–C14 interface. This eC14 is less sensitive to inhibition by EpiCs and enhances resistance to *P. infestans* infection. This strategy demonstrates that a pathogen-inspired protein engineering approach can increase crop resistance to plant pathogens.

## Significance

Genetic engineering to produce crops that are pathogen resistant is an important strategy for world food security. Here, we engineered tomato-secreted immune protease C14 to become less sensitive to inhibition by cystatin-like inhibitor extracellular protease inhibitors of cysteine proteases (EpiCs) secreted by the oomycete late blight pathogen *Phytophthora infestans*, and demonstrated that this engineered C14 (eC14) provides increased immunity. Importantly, this engineering was inspired by our identification of two proteases (Pain1 and Pain2) secreted by *P. infestans* that contribute to virulence and have reduced sensitivity to EpiCs inhibition. Thus, a pathogen avoiding self-inhibition can inspire crop engineering.

The extracellular space (apoplast) in plant tissues is a battleground in plant–pathogen interactions and plays a central role in basal immunity. As the first and main compartment colonized by oomycetes, fungi, and bacteria, the apoplast is a dynamic interface where both host and pathogen deploy numerous secreted proteins and metabolites to either enhance immunity or facilitate infection (1, 2). Plant-secreted hydrolases such as proteases, glycosidases, and lipases are pivotal for extracellular immunity against pathogens. Conversely, successful pathogens secrete inhibitors to suppress the activity of these hydrolases and promote infection (3–5). Consequently, dissecting the function and underlying mechanisms of apoplastic hydrolases and inhibitors is essential to understand the molecular arms race at the host–pathogen interface.

Among these hydrolases, papain-like cysteine proteases [PLCPs, family C1A of clan CA in MEROPS (6)], have emerged as central players in plant immunity (7). Plant PLCPs are required for full resistance against a range of pathogens (7). For instance, the *Arabidopsis thaliana rd19* knockout mutant exhibits increased susceptibility to *Ralstonia solanacearum* (8). In rice, overexpression of *Os*RD21 enhances resistance to the fungal pathogen *Magnaporthe oryzae* (9), while in wheat, *Ta*RD21A functions in immunity against wheat yellow mosaic virus (10). In cotton, *Gh*RD21-7 positively regulates cotton resistance to *Verticillium dahliae* (11), and in *Nicotiana benthamiana*, *Nb*RD21 also plays a positive role in resistance against Chinese wheat mosaic virus infection (12). Remarkably, in maize, the accumulation of Mir1 in leaves confers increased resistance to both caterpillars (13) and herbivores (14). Besides, overexpression of *Fv*RD21 inhibited Strawberry vein banding virus infection in strawberry plants (15). Notably, PLCPs are also targeted by a variety of unrelated pathogen-secreted effectors, which suppress host protease activity to subvert host immunity (7, 16).

Late blight disease caused by the oomycete *Phytophthora infestans* remains a major threat to the worldwide production of potato and tomato (17). Several tomato apoplast PLCPs, including Pip1, Rcr3, and C14, play critical roles in immunity against *P. infestans* (7, 18, 19). To counteract this, *P. infestans* secretes apoplastic cystatin-like effectors such as EpiC1 and EpiC2B that inhibit the activity of these host-derived PLCPs and suppress immune responses (20).

Here, we found that *P. infestans* also secretes PLCPs into the apoplast during infection and that these PLCPs promote the virulence of *P. infestans*. The fact that both plant and pathogen secrete PLCPs raises intriguing questions on their differential specificity to substrates and sensitivity to inhibitors. Inspired by weak EpiC–Pain interactions, we engineered tomato C14, the high-affinity target of EpiCs, to reduce EpiCs inhibition and increase resistance to *P. infestans*.

## Results

### Genome-Wide Identification of PLCPs from *P. infestans*.

**Fig. 1.**
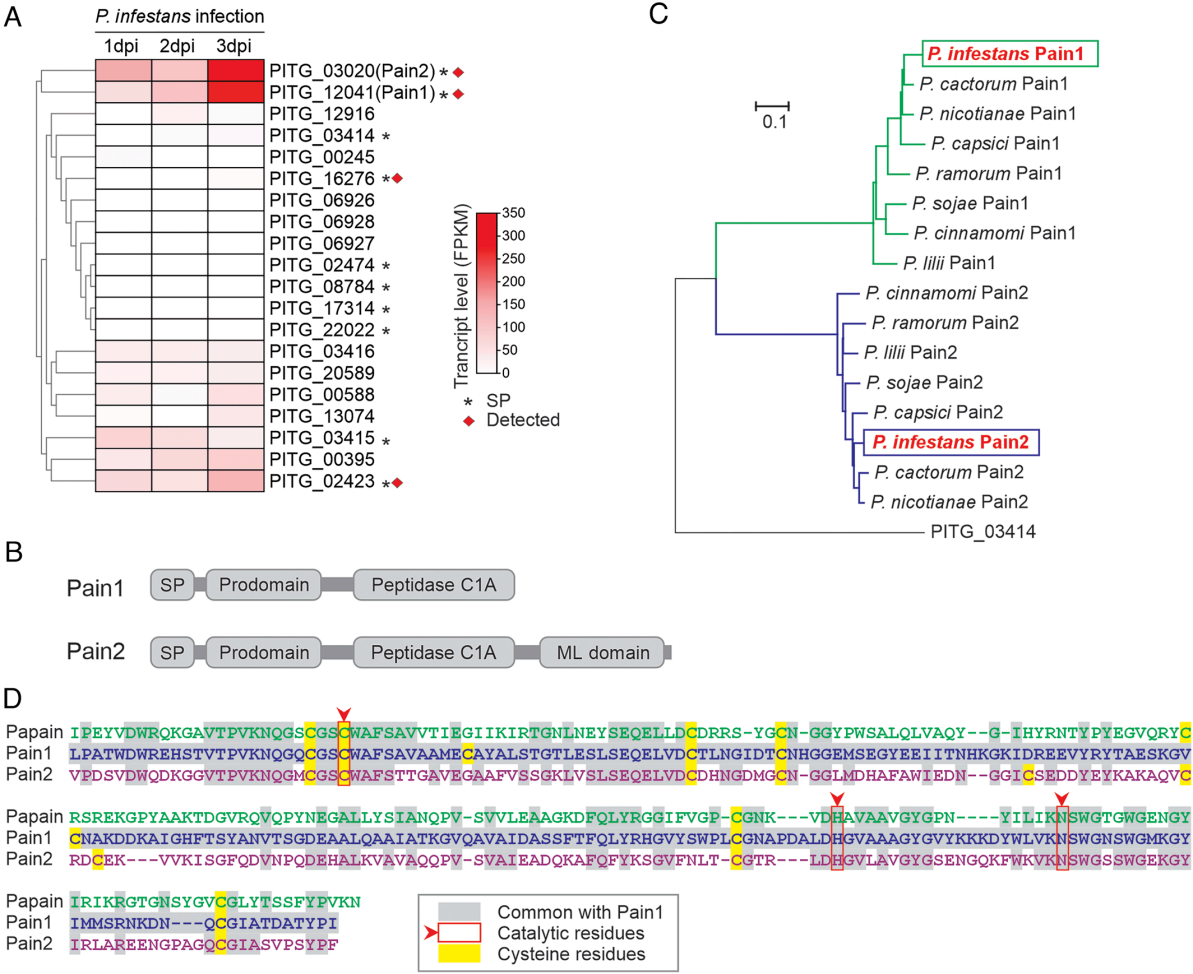
Pain1 and Pain2 are induced and secreted during infection. (*A*) Gene expression profiles of 20 putative PLCPs of *P. infestans*. Ten PLCPs contain predicted signal peptides (asterisks), and four (red squares) have been detected in the *P. infestans* secretome (24). Gene expression data of 20 PLCPs are provided in *SI Appendix*, Table S5. (*B*) Domain architecture of Pain1 and Pain2. Both proteins contain a signal peptide, a prodomain, and a C1A protease domain. In addition, Pain2 possesses a C-terminal ML domain. (*C*) Phylogenetic analysis of Pains orthologs across Phytophthora species based on full-length protein sequences. Homologous sequences were identified in *P. cactorum*, *P. nicotianae*, *P. capsici*, *P. lilii*, *P. ramorum*, *P. sojae*, and *P. cinnamomi*. The secreted PLCP PITG_03414 from *P. infestans* was included as an outgroup. Sequences are provided in Dataset S2. (*D*) Sequence alignment of the protease domains of Pain1 with Papain (*Top*) and Pain2 (*Bottom*). Conserved catalytic residues are highlighted with red arrows and boxes.

Our interest in *P. infestans*-secreted PLCPs was sparked by the detection of two PLCPs (PITG_06927 and PITG_03020) in the apoplast of *P. infestans*–infected tomato plants (21). BLAST searches revealed 20 PLCP-encoding genes in the genome of *P. infestans* (22) (Fig. 1*A*). InterPro domain analysis confirmed that 19 of these genes encode a single protease domain, while one gene, PITG_02423, is predicted to encode a tandem of two protease domains (*SI Appendix*, Fig. S1 and Dataset S1). SignalP and TargetP predictions indicated that 10 of the PLCPs probably carry a signal peptide for secretion (Fig. 1*A*). Notably, genes encoding two PLCPs (PITG_12041 and PITG_03020) carrying signal peptides are highly transcribed during infection of tomato (23) (Fig. 1*A*). These two PLCPs have also been detected by mass spectrometry in the medium of *P. infestans* when grown in cultures (24) (Fig. 1*A*). In addition, PITG_03020 has been detected in the apoplastic fluid of *P. infestans*–infected tomato plants (21), demonstrating that these *P. infestans* PLCPs are secreted into the apoplast during infection.

We named these two proteases Pain1 (PITG_12041) and Pain2 (PITG_03020) as they are papain-like cysteine proteases of *P. infestans*. In addition to the predicted N-terminal signal peptide, both Pain1 and Pain2 contain an inhibitory prodomain and a protease domain (Fig. 1*B*), which is common for PLCPs. Interestingly, Pain2 also features a C-terminal MD-2-related lipid-recognition (ML) domain (Fig. 1*B*), implicated in lipid binding and innate immune signaling (25). Importantly, homologs of both Pain1 and Pain2 are identified in other *Phytophthora* species (Fig. 1*C* and Dataset S2), including soybean root rot pathogen *P. sojae* and sudden oak death pathogen *P. ramorum*. This conservation indicates that these PLCPs may fulfill important roles in the biology of *Phytophthora*, beyond potato late blight.

Alignment with Papain shows that both Pain1 and Pain2 carry the catalytic Cys, His, and Asn residues and additional Cys residues that are probably involved in conserved disulfide bridges that stabilize the protease domains (Fig. 1*D*). The protein alignment also shows that the protein sequence of Pain1 is very different from both Papain and Pain2 (Fig. 1*D*). Indeed, Pain1 and Pain2 share only 46% amino acid sequence identity in their protease domains (Fig. 1*D*).

### Pain1 and Pain2 Are Distinct Papain-Like Cysteine Proteases.

**Fig. 2.**
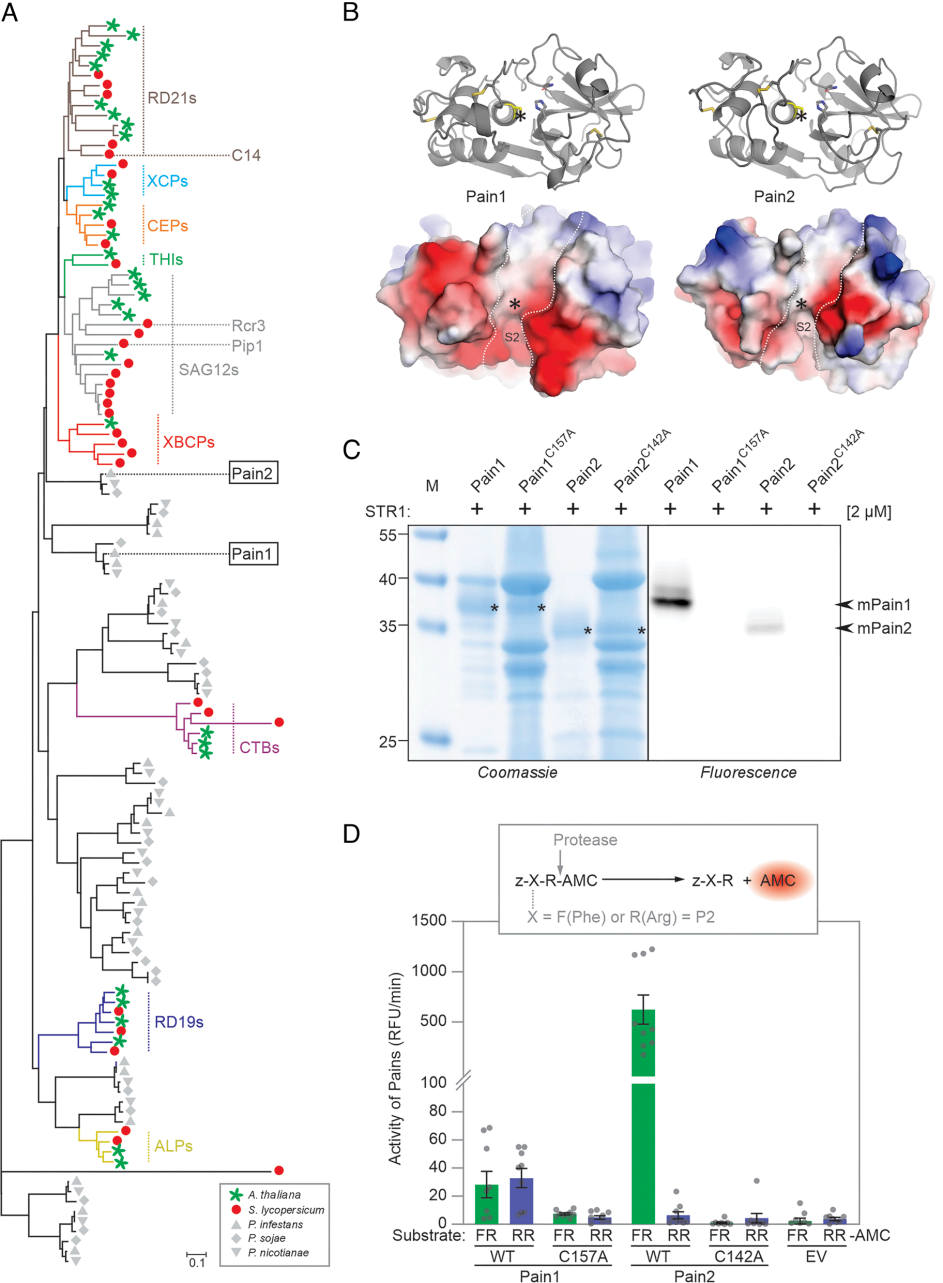
Pain1 and Pain2 represent distinct PLCP subfamilies. (*A*) Evolutionary relationship between *P. infestans*, *P. sojae*, *P. nicotianae*, *A. thaliana*, and *S. lycopersicum* PLCPs based on phylogenetic analysis. Different PLCP subfamilies are indicated in distinct colors. The full tree is shown as *SI Appendix*, Fig. S2, and sequences are provided in Dataset S3. The tree was constructed using the neighbor-joining method using full-length protein sequences. (*B*) AlphaFold-3 predictions of the structures of the Pain1 and Pain2 protease domains. The disulfide bonds are represented in yellow, and the active site cysteine are shown in yellow with an asterisk indicating their position. The surface charge distribution of Pain1 and Pain2 was visualized based on their predicted 3D structures using PyMOL. Positively and negatively charged regions are shown in blue and red, respectively. The substrate binding groove is indicated with the dashed line. (*C*) Pain1 and Pain2 are active proteases. Pain1 and Pain2 and their catalytic mutants were transiently expressed by agroinfiltration and apoplastic fluids isolated at day-5 were labeled with 2 µM STR1 for 3 h at pH 5.0, separated on protein gels, scanned for fluorescence and stained with Coomassie. (*D*) Pain1 and Pain2 have different substrate specificities. Apoplastic fluid from plants transiently expressing Pain1 and Pain2 was isolated at 5 dpi, diluted 16-fold, incubated with 80 μM zFR-AMC and zRR-AMC, and fluorescence was measured as the average value over 10 min. Error bars represent mean ± SE of n = 9 replicates. RFU, relative fluorescence units.

To further investigate the evolutionary origin of Pain1 and Pain2, we performed a comprehensive phylogenetic analysis of all PLCPs from *P. infestans*, *P. sojae*, *P. nicotianae*, *A. thaliana*, and *Solanum lycopersicum*. The results revealed that Pain1 and Pain2 do not cluster with any of the known nine plant PLCP subfamilies (Fig. 2*A* and *SI Appendix*, Fig. S2), indicating that Pains are phylogenetically distinct. Notably, phylogenetic analysis also showed that Pain1 and Pain2 fall into separate clades (Fig. 2*A*), suggesting that they represent very distinct PLCPs. Structural modeling revealed notable differences in the substrate-binding grooves: Pain1 exhibits a higher density of negatively charged residues in the S2 substrate-binding area, in contrast to Pain2, which has uncharged residues (Fig. 2*B*).

We hypothesized that differences in the substrate-binding grooves might lead to divergent substrate specificities. To test this, we produced full-length Pain1 and Pain2 and their catalytic mutants (C157A and C142A for Pain1 and Pain2, respectively) as C-terminally His-tagged proteins by agroinfiltration of *N. benthamiana* and isolated apoplastic fluids (AFs) at 5 days postinfiltration (5 dpi). Unfortunately, Western blot analysis of these AFs with anti-His antibodies did not reveal a signal (*SI Appendix*, Fig. S3*A*), possibly because the epitope tag is cleaved off.

We next incubated AFs with the fluorescent activity-based probe STR1, which contains a bodipy fluorophore and an E-64 warhead (*SI Appendix*, Fig. S3*B*). Separation of STR1-labeled proteins on protein gels followed by fluorescence scanning revealed that both Pain1 and Pain2 produce robust signals that are absent for the catalytic mutants (Fig. 2*C*). These signals were detected at apparent molecular weights that are consistent with the predicted molecular weights of the mature proteins.

We next selected two commercially available fluorogenic dipeptides containing the hydrophobic Phe (F) or basic Arg (R) at the P2 position (zFR-AMC and zRR-AMC, respectively), to test the observation from structural modeling (Fig. 2*B*) that Pain2 prefers less basic substrates. Incubation of AFs containing Pain1 and Pain2 with the dipeptide substrates indeed revealed that Pain2 is more active toward zFR-AMC, whereas Pain1 has similar activity toward zFR-AMC and zRR-AMC (Fig. 2*D* and *SI Appendix*, Fig. S3*C*). In contrast, the catalytic mutants Pain1^C157A^ and Pain2^C142A^ exhibited little to no detectable activity. These data confirm that Pain1 and Pain2 have different cleavage specificities and might have different natural substrates.

### Pains Promotes *P. infestans* Infection in *N. benthamiana* Leaves.

**Fig. 3.**
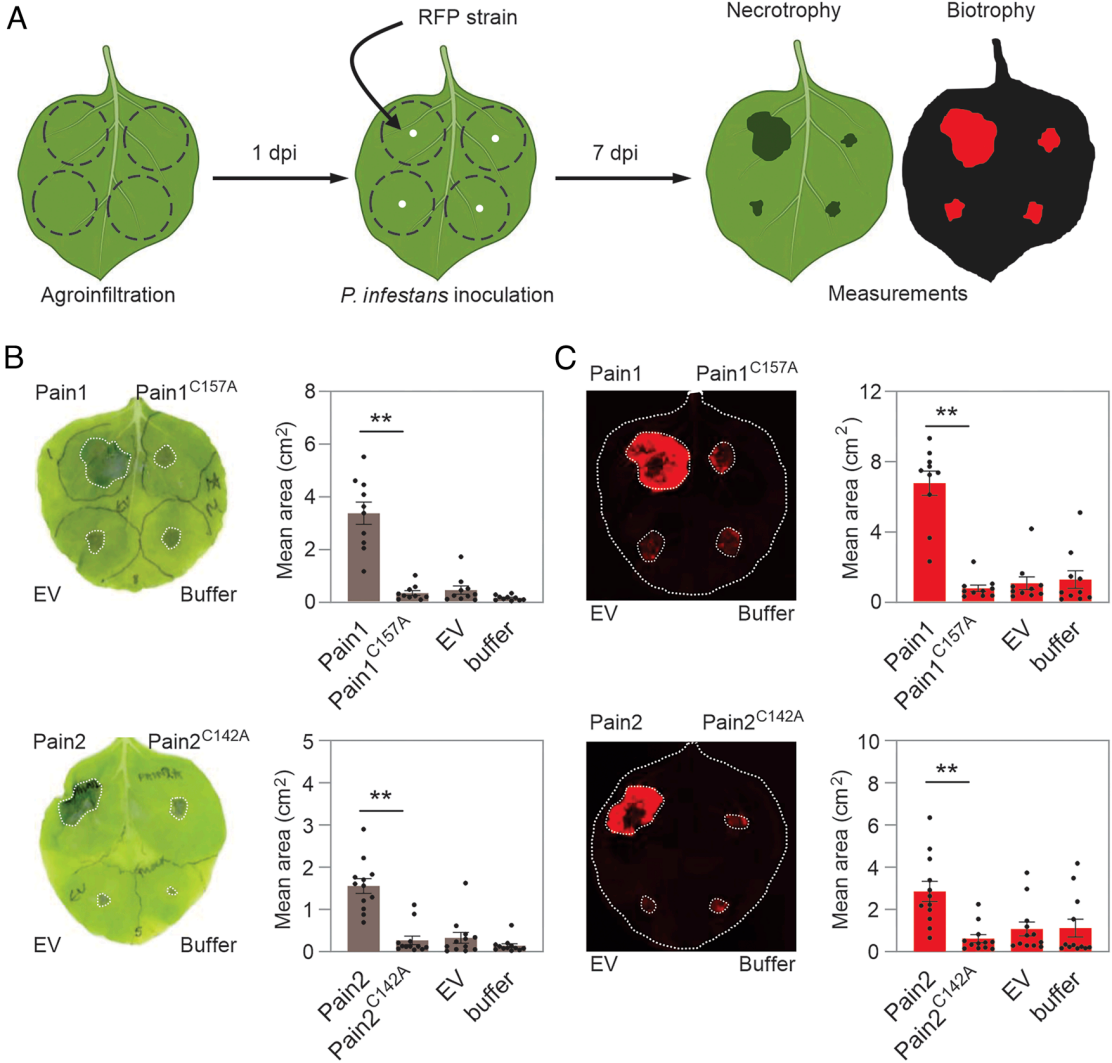
Pains promote *P. infestans* infection. (*A*) Method for testing the hypothesis that Pains promote the virulence of *P. infestans*. Agrobacteria carrying constructs encoding Pain1, Pain2, their catalytic mutants (Pain1^C157A^ and Pain2^C142A^), an empty vector control, or infiltration buffer were infiltrated into four distinct sites on the same *N. benthamiana* leaf. One day postinfiltration, the leaves were detached and inoculated with *P. infestans* strain 88069td, which expresses the red fluorescent protein (tandem dimer RFP) (18). Inoculated leaves were maintained in a growth chamber at 18 °C in darkness for 7 d. Disease progression was then assessed by measuring both necrotrophy (visible lesion area) and biotrophy (RFP-labeled *P. infestans*). (*B* and *C*) Quantification of visible necrotic lesion areas (*B*) and RFP fluorescent pathogen growth areas (*C*). Each dot represents an individual biological replicate. Means and SE from different replicates are shown (**P < 0.01; Student’s *t* test).

To further investigate the functional role of Pains, we transiently expressed Pain1, Pain2, their corresponding catalytic mutants, an empty vector, and an infiltration buffer control in *N. benthamiana* leaves. At one day postinfiltration, leaves were challenged with a transgenic *P. infestans* strain (88069td) constitutively expressing a red fluorescent protein (18), and areas of necrotrophy (lesions) and biotrophy (red fluorescence) were quantified at 7 days post infection (7 dpi) (Fig. 3*A*). Transient expression of Pain1 and Pain2 in *N. benthamiana* leaves significantly promoted *P. infestans* colonization, as evidenced by increased areas of both biotrophic and necrotrophic infection (Fig. 3 *B* and *C*). In contrast, only small lesions were detected in leaves expressing their inactive mutant, the empty vector, or those infiltrated with infiltration buffer (Fig. 3 *B* and *C*). These results demonstrate that Pains promote *P. infestans* infection on *N. benthamiana* leaves, depending on the active site Cys residue.

### *P. infestans*-Secreted EpiCs Preferentially Inhibit Tomato C14 Over Pain1/2.

**Fig. 4.**
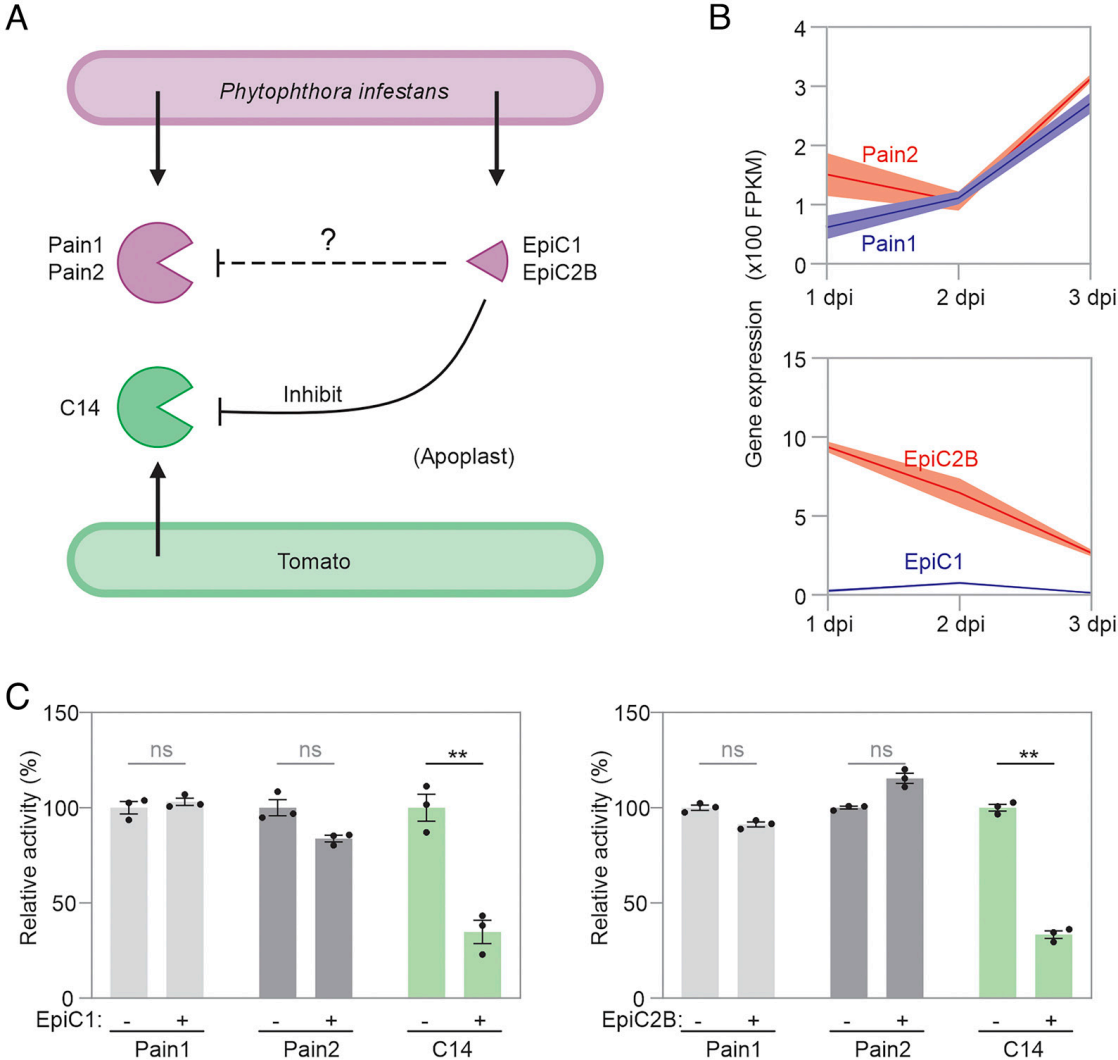
EpiCs preferentially inhibit C14 over Pains. (*A*) Both *P. infestans* and tomato secrete PLCPs into the apoplast during infection. *P. infestans* also delivers PLCP inhibitors, EpiC1 and EpiC2B, into the same compartment. While the inhibition of plant-derived PLCPs by EpiCs has been well characterized (20), it remains unclear whether EpiC1 and EpiC2B suppress the activity of *P. infestans*-derived Pains. (*B*) The gene expression of Pains and EpiCs during infection. The raw data are provided in *SI Appendix*, Table S6. (*C*) EpiC1 and EpiC2B selectively inhibit C14 activity, while failing to suppress the activity of Pain1 and Pain2. Apoplastic fluids from agroinfiltrated *N. benthamiana* leaves transiently expressing Pain1, Pain2, or C14 were preincubated for 30 min with 10 μL 16 ng/μL EpiC1 or EpiC2B, followed by labeling for 3 h with 2 μM TK011. Samples were separated by SDS-PAGE and scanned for fluorescence. Signal intensities were quantified and are shown as percentages relative to the labeling signal without inhibitor treatment. See *SI Appendix*, Fig. S5 for the dose-dependent inhibition of protease activity by EpiC1 and EpiC2B.

In addition to Pain1 and Pain2, *P. infestans* also secretes cystatin-like inhibitors EpiC1 and EpiC2B into the apoplast during infection (Fig. 4*A*). Whereas the interaction between EpiCs and plant-secreted Pip1, Rcr3, and C14 of tomato has been well characterized (20), we wondered if EpiCs also inhibit Pain1 and Pain2. We expect both proteins to coexist in the apoplast of infected plants, even though the transcript levels of both Pain-expressing genes are expressed higher at later stages of infection, while expression of both EpiC-encoding genes declines (Fig. 4*B*). Indeed, both Pains and EpiCs can be detected by mass spectrometry in the secretome of *P. infestans* (24), suggesting that they may coexist in the apoplast of infected plants.

Since EpiCs inhibit C14 with greater affinity than Pip1 and Rcr3 (20), we compared C14 and Pains to assess their relative sensitivity to inhibition by EpiCs. We incubated AF containing Pain1, Pain2, or C14 with increasing concentrations of EpiC1 and EpiC2B (*SI Appendix*, Fig. S4), and then labeled the remaining proteases with an activity-based probe. Interestingly, labeling of Pain1 and Pain2 was only reduced upon incubation with high EpiCs concentrations, in contrast to tomato C14, which was inhibited upon preincubation with low concentrations of EpiCs (*SI Appendix*, Fig. S5). These results suggest that *P. infestans* uses EpiCs to selectively inhibit host immune proteases like C14, while avoiding inhibition of its own proteases, such as Pain1 and Pain2 (Fig. 4*C*). This indicates a strategy by which the pathogen distinguishes between self and host hydrolase to promote infection.

### Engineered C14 Enhance Resistance against *P. infestans*.

**Fig. 5.**
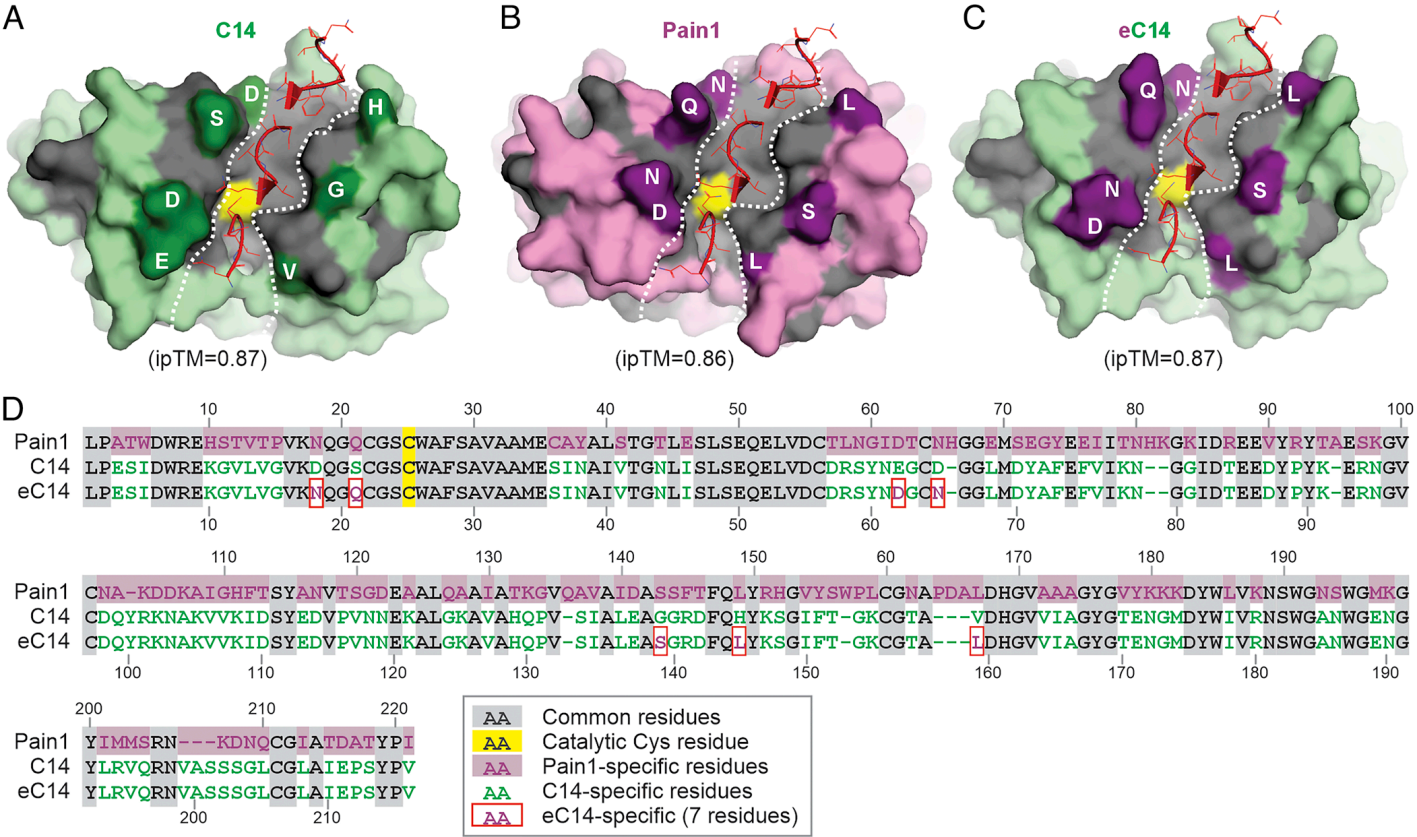
Design of engineered eC14 with Pain1 residues from the EpiC2B-interface. (*A*–*C*) Structural model of complexes of EpiC2B with C14, Pain1, and eC14, predicted with AlphaFold3. Highlighted are the three interacting loops of EpiC2B (red), the substrate-binding groove (white dashed line), and the catalytic Cys residue (yellow). Key residues that differ between C14 and Pain1 at the EpiC2B interface are marked in dark green and purple, respectively. (*D*) Protein sequence alignment of the catalytic domains of Pain1, C14, and eC14, with seven amino acid substitutions highlighted (red boxes).

Given that C14 activity is suppressed by cystatin-like EpiCs while Pains remain largely unaffected, we hypothesized that we could use the avoidance of EpiC–Pain self-inhibition to engineer an EpiCs-insensitive C14 to enhance plant resistance to *P. infestans*. Cystatins interact with PLCPs via the tripartite wedge consisting of three loops: the N-terminal loop, the central loop containing the QxVxG motif, and the C-terminal loop (CTL), containing a conserved Trp (W) residue (26). Models of EpiC2B–C14 and EpiC2B–Pain1 complexes generated with AlphaFold3 (27) are consistent with cystatin–papain complexes. To identify contact residues, we selected all residues in the proteases that are within 5 Å of the tripartite wedge of EpiC2B (*SI Appendix*, Fig. S6) and mapped these onto structurally aligned protein sequences (*SI Appendix*, Fig. S7 *A* and *B*). This procedure identified seven residues in C14 at the interface that are different in Pain1 and may be critical for the interaction with EpiC2B (Fig. 5 *A*–*C* and *SI Appendix*, Fig. S7*C*). Importantly, all these residues are located outside the substrate-binding groove (Fig. 5 *A*–*C*), suggesting that these residues can be substituted without compromising the protease function of C14. We next replaced these seven residues in wild-type C14 with the corresponding Pain1 residues to generate an engineered C14 (eC14) (Fig. 5).

**Fig. 6.**
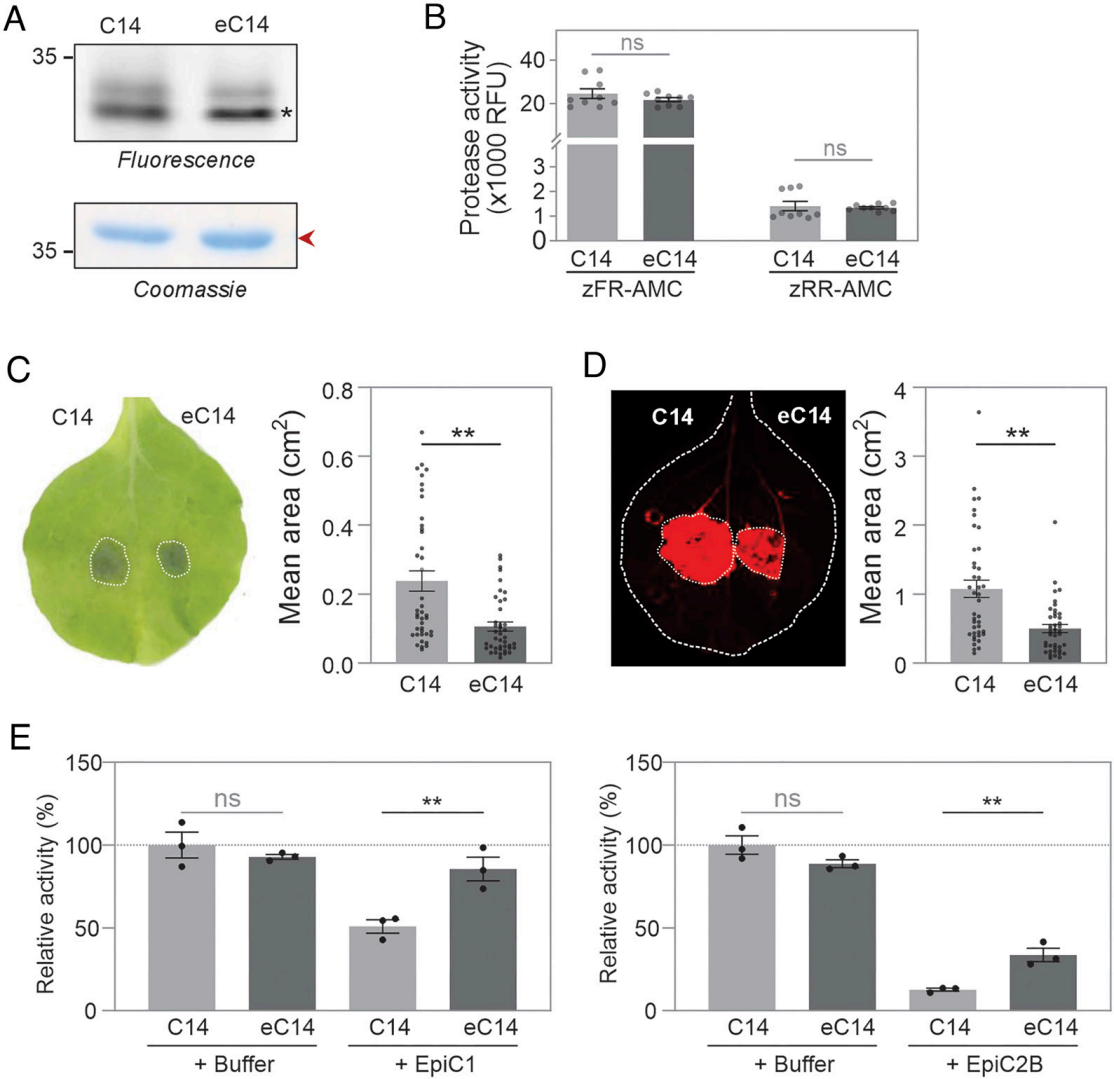
Engineered eC14 enhances resistance to *P. infestans*. (*A*) Wild-type C14 and eC14 have similar activity in apoplastic fluid. Apoplastic fluids isolated from agroinfiltration leaves transiently expressing wild-type C14 and eC14 were labeled with 2 µM STR1, and proteins were analyzed on protein gels by fluorescence scanning and Coomassie staining. Endogenous PR2 (red arrow) demonstrates that similar amounts of protein were loaded. The asterisks indicate the sizes of the mature wild-type C14 and eC14, respectively. (*B*) Engineered eC14 has unaltered protease activity. Apoplastic fluid was isolated at 5 dpi from *N. benthamiana* plants transiently expressing wild-type C14 and eC14. Samples were incubated with 80 μM zFR-AMC or zRR-AMC substrates in the presence of 10 mM TCEP and 50 mM sodium acetate buffer (pH 5.0) for 5.5 min. Data are presented as mean ± SE from n = 9 measurements, derived from three biological replicates, each with three technical replicates. RFU, relative fluorescence units. (*C* and *D*) Overexpression of engineered C14 in *N. benthamiana* enhances resistance against *P. infestans*. *N. benthamiana* leaves were agroinfiltrated with wild-type C14 or eC14. One day postinfiltration, the leaves were detached and inoculated with *P. infestans* strain 88069td. Visible necrotic lesion areas and RFP fluorescent pathogen growth areas are both outlined with dashed lines and quantified. Each dot represents a biological replicate. Data are presented as mean ± SE (*P* < 0.01, Student’s *t* test). The experiment was repeated twice with similar results. (*E*) Compared to wild-type C14, eC14 is less sensitive to inhibition by EpiC1 and EpiC2B. Apoplastic fluids isolated from *N. benthamiana* leaves transiently expressing wild-type C14 or eC14 were preincubated for 10 min with 50 ng of EpiC1 or EpiC2B in the presence of 50 mM Tris-HCl, pH 7.5, 150 mM NaCl, and 5 mM TCEP, followed by labeling with 2 μM STR1 for 30 min. Samples were separated by SDS-PAGE, scanned for fluorescence, and fluorescence intensity was determined for the C14-derived signals. Error bars represent the SE of n = 3 replicates. Statistics were calculated with a Student’s *t* test. Original gels are shown in *SI Appendix*, Fig. S9.

To evaluate if eC14 enhances resistance to *P. infestans*, we transiently expressed both wild-type C14 and eC14 in opposite leaf halves of *N. benthamiana*. Activity-based labeling on apoplastic fluids isolated from these leaves showed that eC14 and wild-type C14 have very comparable active levels (Fig. 6*A*). Their activity toward fluorogenic dipeptide substrates is also very comparable (Fig. 6*B* and *SI Appendix*, Fig. S8 *A* and *B*).

We next inoculated the agroinfiltrated leaves with *P. infestans* and monitored disease progression at 7 days postinoculation. Importantly, we observed a significant reduction in both biotrophic and necrotrophic infection areas in eC14-expressing leaf halves, when compared to wild-type C14-expressing leaf halves (Fig. 6 *C* and *D*), indicating that engineering C14 increases late blight resistance. Moreover, we found that eC14 also enhances resistance to *P. capsici* (*SI Appendix*, Fig. S8*C*), which secretes nearly identical EpiCs.

To investigate whether the enhanced resistance conferred by eC14 correlates with reduced inhibition by EpiCs, we preincubated AF containing wild-type C14 or eC14 with low concentrations of EpiC1 and EpiC2B and then labeled the remaining noninhibited proteases with STR1. Activity-based labeling of wild-type C14 was strongly inhibited by EpiCs, whereas eC14 activity was only slightly reduced (*SI Appendix*, Fig. S9). Notably, the inhibitory effect of EpiCs on wild-type C14 and eC14 is significantly different (Fig. 6*E*). These results demonstrate that the enhanced disease resistance mediated by eC14 can result from reduced sensitivity to EpiCs-mediated inhibition.

## Discussion

In this study, we identified and functionally characterized two previously unrecognized *P. infestans*-secreted PLCPs, Pain1 and Pain2. These two proteases promote *P. infestans* virulence, and this is dependent on their protease activity (Fig. 3). We further demonstrate that *P. infestans*-secreted cystatin-like protease inhibitors, EpiC1 and EpiC2B, preferentially inhibit host C14 rather than Pain1 and Pain2 (Fig. 4), suggesting a coevolutionary adaptation to avoid self-inhibition. To mimic this evasion strategy, we engineered a C14 variant (eC14) by introducing seven residues from Pain1 that are likely to disrupt the EpiC2B–C14 interaction interface. This modified eC14 exhibits reduced sensitivity to EpiCs-mediated inhibition and confers enhanced resistance to *P. infestans* infection.

This finding is significant as it highlights that both host and pathogen use the same class of protease in opposite roles: to enhance plant immunity and increase pathogenicity, respectively. This implies that their substrates in the apoplast are very likely different from each other. Identifying the substrates of these proteases would provide key insights into their biological functions. However, substrate identification for proteases remains technically challenging. The development of protease-trap technologies (28, 29) has provided a promising new avenue for capturing substrates and could be employed to uncover the direct targets of proteases in the apoplast. Additionally, cutting-edge tools such as HUNTER (30) (high-efficiency undecanal-based N termini enrichment) and structural predictions from AlphaFold3 (27) will undoubtedly be helpful to identify substrates. The fact that both host and pathogen secrete PLCPs into the apoplast raises questions on how they distinguish friend from foe. We demonstrated that tomato C14 is strongly inhibited by EpiCs, whereas the pathogen-derived Pain1 and Pain2 proteases are only weakly inhibited by EpiCs (Fig. 4*C*). This observation suggests that *P. infestans* has evolved a discriminatory mechanism to selectively target host proteases, thereby avoiding self-inhibition during infection.

Over the past decades, numerous studies have shown that plant-secreted PLCPs contribute to plant immunity (7). In contrast, much less is known about the roles of pathogen-secreted PLCPs. Only a few pathogen-produced PLCPs have been characterized. For example, *Pp*Cys44 and *Pp*Cys45 from *P. parasitica* (also named *P. nicotianae*) induce cell death in multiple *Nicotiana* species and function as virulence factors (31). However, Pain1 and Pain2 do not induce cell death in *N. benthamiana*, and phylogenetic analysis indicates that they are not orthologous to *Pp*Cys44 or *Pp*Cys45. Our observations suggest that *Phytophthora*-secreted PLCPs may employ diverse mechanisms to promote infection, and their functions likely extend beyond eliciting cell death. Similarly, *Xo*CP from *Xanthomonas oryzae* pv. oryzae contributes to bacterial pathogenicity, as knockout mutants of *xocp* exhibit reduced virulence on rice (32). However, our study differs from this work in several important aspects. First, Pain1 and Pain2 originate from an oomycete pathogen, whereas *Xo*CP is produced by a bacterium, suggesting that PLCPs have independently evolved as virulent factors across distinct pathogens. Second, *Xo*CP targets a monocot host (rice), while Pain1 and Pain2 are studied in the context of dicot plants such as *N. benthamiana* and tomato. These differences suggest that different pathogens may use PLCPs in distinct ways to infect their hosts. Interestingly, the oomycete root pathogen *Aphanomyces euteiches* also secretes various active modular papain-like proteases into the pea apoplast during infection, suggesting that the use of modular pathogen-secreted PLCPs might be common to oomycete plant pathogens (33).

We found that the protease domains of Pain1 and Pain2 fall into distinct phylogenetic clades (*SI Appendix*, Fig. S1), suggesting that they represent functionally divergent PLCPs. Consistently, they share only 46% amino acid sequence identity within their protease domains. Activity assays using fluorogenic dipeptide substrates further support this divergence: Pain2 shows a clear substrate preference for zFR-AMC, whereas Pain1 exhibits comparable activity toward both zFR-AMC and zRR-AMC (Fig. 2*D*). These findings strongly suggest that Pain1 and Pain2 have evolved distinct specificities and likely fulfill different biological functions. Notably, orthologs of Pain1 and Pain2 are also conserved in other Phytophthora species, including the soybean root rot pathogen *P. sojae* and the sudden oak death pathogen *P. ramorum*, indicating that both these PLCPs have been maintained during Phytophthora evolution, likely due to their essential but distinct roles in pathogenesis. Elucidating the functional divergence between Pain1 and Pain2 will deepen our understanding of how Phytophthora species deploy distinct proteases to suppress host immunity.

Transient expression of Pain1 and Pain2 in *N. benthamiana* promotes pathogen infection, suggesting that these two proteases function as virulent factors. If the essential role of endogenous Pain1 and Pain2 in *P. infestans* pathogenicity can be confirmed, this knowledge could facilitate the development of disease control strategies. One promising method would be the design of inhibitors that specifically target Pains without interfering with host PLCPs. With recent advances in de novo protein design (34), it will be feasible to develop protein-based inhibitors that only bind to Pains and suppress their activity. Alternatively, high-throughput screening could be employed to identify small-molecule compounds that specifically inhibit Pains activity. For example, a recent study identified FY21001, a compound that binds *Mo*Ers1 of *Magnaporthe oryzae*, and disrupts the *Mo*Ers1–*Os*RD21 interaction, effectively controlling rice blast disease (9). Similar approaches could be employed to interfere with Pains-mediated immune suppression, offering a potential new route for controlling Phytophthora disease.

Notably, Pain2 carries a C-terminal ML domain, which in animal systems is often associated with lipid binding and innate immune signaling (25). Although its precise role in Phytophthora remains unclear, it may be involved in host–pathogen interactions or lipid-mediated localization. One hypothesis is that the ML domain targets Pain2 to specific apoplastic niches through lipid binding. For example, the *Arabidopsis* ML protein ML3 undergoes NEDD8 conjugation, which may regulate its subcellular localization and activity (35). Besides, pathogens have been reported to exploit ML domain-related pathways. For instance, the dengue virus upregulates ML domain proteins in its mosquito host to facilitate infection (36). Although ML domain-containing proteins are also present in tomato, the host of *P. infestans*, their functions remain poorly understood. Further investigation is needed to determine whether this domain contributes to *Phytophthora* virulence by mediating interactions with lipid or by interfering with host ML domain-dependent immune processes.

Transient expression of Pains in *N. benthamiana* results in active proteases that lack the pro-domain. However, whether this activation is due to autocatalytic processing or mediated by host proteases remains unclear. Previous research has shown that the prodomain removal of *Arabidopsis* RD21 is mediated by a proteolytic cascade, whereas granulin domain removal requires autocatalytic processing (37). Whether a similar mechanism applies to Pain2 warrants further investigation.

In addition to EpiC1 and EpiC2B, six other EpiC homologs are present in the *P. infestans* genome. To further determine whether the resistance conferred by eC14 depends on the presence of these EpiC proteins, the ideal strategy would be to generate an EpiCs-null mutant of *P. infestans* using CRISPR/Cas-mediated genome editing. However, as this technology is not yet established for *P. infestans*, it is currently not feasible to experimentally assess whether the resistance mediated by eC14 is EpiCs-dependent. Another limitation to note is that both EpiC2B–C14 and EpiC2B–Pain1 complexes show high ipTM scores (Fig. 5 *A* and *B*), EpiC2B inhibits C14 but has much less effect on Pain1 (Fig. 4*C*). This observation highlights a common limitation of AlphaFold-based predictions, which can occasionally predict false-positive interactions (38).

We found that although tomato and *P. infestans* secrete the same protease class into the apoplast during infection, pathogen-secreted EpiCs inhibitors preferentially target tomato protease C14, while avoiding inhibition of self-produced Pains. This observation suggests that *P. infestans* can distinguish friend from foe proteases and avoid self-inhibition during infection. We used that knowledge to select seven amino acid residues in C14 that may be critical for EpiC2B binding. Substitution of these residues in C14 with seven Pain1 residues resulted in an engineered variant, eC14, that retained proteolytic activity yet exhibited reduced sensitivity to EpiCs-mediated inhibition, and significantly enhanced resistance to *P. infestans* and *P. capsici*. Notably, C14-like proteases are widely conserved across plant species (16), and orthologs of EpiCs (39) and Pains (Fig. 1*C*) are present in numerous *Phytophthora* genomes. Therefore, engineering C14 homologs in other plants using genome-editing (40, 41) might be an effective strategy to enhance resistance against diverse Phytophthora pathogens. Recently, a structural model of the EpiC2B–Pip1 complex facilitated the design of an EpiC2B-insensitive Pip1 that not only avoided EpiC2B inhibition but also conferred enhanced resistance to *P. infestans* (42). Our study with eC14 further strengthens the concept that engineering of plant immune proteases to evade inhibition by pathogens represents a powerful strategy to enhance plant immunity against pathogens.

## Materials and Methods

### Plants Material and *P. infestans* Strain Cultivation.

Four-week-old *N. benthamiana* plants (LAB genotype) were cultivated in a greenhouse under a 16-h light (80 to 120 µmol m^−2^ s^−1^)/8-h dark cycle at temperatures of 21 °C (night) and 22 to 23 °C (day) before use in the experiments. *P. capsici* strain LT263 was routinely cultured on solid 10% (v/v) V8 agar medium at 22 °C in the dark (43). The *P. infestans* (88069td) (18) was grown on the rye sucrose agar-V8 medium at 18 °C in the dark as previously described (23).

### Plasmid Construction.

The gene sequence of *Pain1* and *Pain2* (with an N-terminal NtPR1a signal peptide and a C-terminal His tag, see *SI Appendix*, Table S1) was synthesized at Twist Bioscience and inserted into the binary vector pJK187 (44) to yield NtPR1a-Pain1-His (pAJVP001) and NtPR1a-Pain2-His (pHJ014) using the ClonExpress Ultra One Step Cloning Kit (Cat. No. C115, Vazyme Biotech). The catalytic mutants of Pain1 and Pain2 were generated using their respective mutagenic primers and subsequently inserted into the pJK187 vector, yielding NtPR1a-Pain1^C157A^-His (pAJVP006) and NtPR1a-Pain2^C142A^-His (pAJVP007). The pJK590#07_SP (*SI Appendix*, Table S2) vector (45), used for overexpressing wild-type C14 in *N. benthamiana*, was kindly provided by Jiorgos Kourelis. The partial sequence of eC14 (*SI Appendix*, Table S1) was synthesized at Twist Bioscience and cloned into pJK590#07_SP, which was linearized by PCR with pJK590#07_SP linearized primers, resulting in pHJ143 (pL2M-P19-2x35S::eC14). All plasmids were sequenced using Source Bioscience using PJK187 vector primers to confirm the inserts. The prokaryotic expression vectors of EpiC1 and EpiC2B were constructed by assembling inserts from pJP001, pJK120, pFGH029, and pJP002 into the pJK082 backbone using a BsaI Golden Gate reaction, resulting in pJK155 (pET28b-T7::OmpA-His-TEV-EpiC1) and pJK157 (pET28b-T7::OmpA-His-TEV-EpiC2B), as previously described (42, 46). All plasmids and primers used in this work are listed in *SI Appendix*, Tables S3 and S4, respectively.

### Transient Protein Expression in *N. benthamiana* and AFs Isolation.

To transiently express proteins in *N. benthamiana*, plasmids were first transformed into *Agrobacterium tumefaciens* GV3101(pMP90), and positive transformants were selected on LB agar plates containing 50 µg mL^−1^ kanamycin, 25 µg mL^−1^ rifampicin, and 10 µg mL^−1^ gentamicin. Overnight cultures of agrobacteria were harvested by centrifugation at 4,000 g for 5 min, and the cells were resuspended in infiltration buffer (10 mM MgCl_2_, 10 mM MES-KOH, pH 5.6, and 150 µM acetosyringone). The cultures were then mixed at a 1:1 ratio with agrobacteria carrying the silencing suppressor P19 (47) to a final OD_600_ of 0.5. After incubation at room temperature for 2 to 3 h, the agrobacteria suspension was infiltrated into 4-wk-old *N. benthamiana* leaves using a needleless syringe. Leaves were harvested for 5 days postinfiltration, washed briefly with Milli-Q water, and then submerged in ice-cold Milli-Q water. Vacuum infiltration was performed twice for 2 to 3 min each time, then leaf surfaces were gently dried with absorbent paper, and the leaves were carefully placed into an empty 20 mL syringe inserted into a 50 mL Falcon tube. AFs were collected by centrifugation at 500 to 1,000 g for 20 to 30 min at 4 °C.

### Fluorogenic Peptide Assay.

PLCP protease activity was measured using the Z-Phe-Arg-7-amino-4-methylcoumarin (zFR-AMC) and Z-Arg-Arg-7-amino-4-methylcoumarin (zRR-AMC) fluorogenic substrates. AFs were mixed with 80 µM fluorogenic substrates in a Corning 96-well clear-bottom black plate in the presence of 10 mM TCEP and 50 mM sodium acetate buffer (pH 5.0), at a final reaction volume of 100 µL. Fluorescence was measured with an excitation wavelength of 380 nm and an emission wavelength of 460 nm. Protease activity was calculated based on the change in fluorescence over time. Three biological replicates and three technical replicates were analyzed for each sample.

### Activity-Based Protein Profiling and Western Blot of Pains.

The AFs from Pain1, Pain2, Pain1^C157A^, Pain2^C142A^ were incubated at room temperature in the dark for 3 h in a total reaction volume of 60 µL containing 50 mM sodium acetate buffer (pH 5.0), 5 mM TCEP, and 2 µM STR1. The labeling reaction was stopped by adding 4× loading buffer [200 mM Tris-HCl (pH 6.8), 400 mM DTT, 8% SDS, 0.2% bromophenol blue, 40% glycerol] and heating for 5 min at 95 °C. Samples were separated on the 15% SDS-PAGE gel and scanned for fluorescence using the Typhoon scanner with Cy3 settings. The gel was then stained with InstantBlue® Coomassie Protein Stain (ISB1L) (Cat. No. ab119211, Abcam). For His-tag detection, samples were separated on the gel and detected with a His antibody (Cat. No. R931-25, Invitrogen).

### Virulence Assay.

For *P. infestans* inoculation, Agrobacteria infiltrated *N. benthamiana* leaves were detached 1-d postagroinfiltration and inoculated with 10 µL of a *P. infestans* (88069td) zoospore suspension (approximately 200-300 zoospores per microliter). The inoculated leaves were incubated in a growth chamber at 18 °C in the dark. Seven days postinoculation, the lesion areas (cm^2^) were scanned using an Epson scanner with a ruler for scale. Additionally, the growth of RFP-labeled *P. infestans* was visualized using an Amersham Typhoon scanner with Cy3 settings. Both the area of necrotrophy (visible lesion areas) and biotrophy (RFP-labeled *P. infestans*) were quantified using ImageJ. For *P. capsici* inoculation, *N. benthamiana* leaves infiltrated with Agrobacterium were detached 3 to 4 days postinfiltration and inoculated with *P. capsici* mycelium (5 mm). The inoculated leaves were incubated at 22 °C in the dark. Two days postinoculation, lesion areas (cm^2^) were scanned using an Epson scanner with a ruler for scale and quantified using ImageJ.

### Expression and Purification of EpiCs.

Plasmids pJK155 (EpiC1) and pJK157 (EpiC2B) were transformed into the *Escherichia coli* Rosetta (DE3) strain. Protein expression was induced with 0.1 mM IPTG at 20 °C overnight. Recombinant proteins were purified using HisPur™ Ni-NTA Resin (Cat. No. 88222, Thermo Scientific) following the manufacturer’s instructions and subsequently concentrated using a 15 mL Amicon Ultra centrifugal filter device with a 3 kDa molecular weight cut-off (Millipore). Purified EpiCs were confirmed by SDS-PAGE followed by Coomassie staining and western blotting using an N-terminal anti-His (HRP-conjugated) antibody (Cat. No. 130-092-785, Miltenyi Biotec). The protein concentration of purified proteins was measured using the Bio-Rad RC DC™ protein assay kit. Purified proteins were aliquoted and stored at −80 °C until further use.

### Inhibition Assays.

For ABPP inhibition assay of Pain1, Pain2, and C14, to ensure comparable activity levels of Pain1, Pain2, and C14, AF samples were normalized by dilution in a 10:80:1 ratio using Milli-Q water. The inhibitors EpiC1 and EpiC2B were serially diluted in reaction buffer (50 mM sodium acetate, pH 5.0, and 5 mM TCEP) to final concentrations of 64, 32, 16, 8, 4, 2,1 and 0 ng/µL. For each reaction, 10 µL of diluted inhibitor was added to 50 µL of AF and incubated for 30 min at room temperature. Subsequently, the samples were incubated with 2 µM TK011 (42) for 3 h at room temperature. Protein precipitation was performed by adding four times volume of ice-cold acetone, followed by centrifugation at 10,000 g for 2 to 3 min. The supernatant was discarded, and the tubes were left open for 10 to 20 min to allow residual acetone to evaporate. Precipitated proteins were resuspended in 20 µL Milli-Q water and mixed with 10 µL of 4× protein loading dye. Samples were separated by SDS-PAGE, and in-gel fluorescence was visualized using a Typhoon scanner (Cy2 channel). Fluorescence intensity was quantified using ImageJ.

For the ABPP inhibition assay of C14 and eC14, equal volumes of AF from C14 and eC14 samples were used in the assay. Given that some eC14 mutation sites altered residue polarity, the assay was performed at pH 7.5 to ensure optimal stability of the proteins. The inhibitors EpiC1 and EpiC2B were diluted in reaction buffer (50 mM Tris-HCl, pH 7.5, 5 mM TCEP, and 150 mM NaCl) to a final concentration of 5 ng/µL. For each reaction, 10 µL of inhibitor solution was added to 10 µL of AF and incubated for 10 min at room temperature. Subsequently, samples were incubated with 2 µM STR1 for 30 min at room temperature, followed by the addition of 8 µL 4× protein loading dye. Samples were separated by SDS-PAGE, and in-gel fluorescence was detected using an Amersham Typhoon scanner with Cy3 settings.

### Bioinformatic Analysis.

The phylogenetic tree analysis was done using MEGA software. The protein structures were predicted using AlphaFold Server (https://alphafoldserver.com/) and further analyzed using PyMOL software. SignalP-6.0 (https://services.healthtech.dtu.dk/services/SignalP-6.0/) and TargetP-2.0 (https://services.healthtech.dtu.dk/services/TargetP-2.0/) were used for signal peptide prediction. InterPro (https://www.ebi.ac.uk/interpro/) was used for domain prediction.

## Supplementary Material

Appendix 01 (PDF)

Dataset S01 (PDF)

Dataset S02 (PDF)

Dataset S03 (PDF)

## Data Availability

Study data are included in the article and/or supporting information.
