# Supplementary material for: Pathogen-inspired engineering of plant protease enhances late blight resistance
Source: Proc Natl Acad Sci U S A. 2026 Jan 9;123(2):e2524700123. doi: 10.1073/pnas.2524700123 (PMC12799129; doi:10.1073/pnas.2524700123)
Supplement: Supplementary file 1 — Appendix 01 (PDF) [file pnas.2524700123.sapp.pdf]

PNAS

Supporting Information for:

Pathogen-inspired engineering of plant protease enhances late blight resistance

Jie Huang (黄杰), Alice Penrose, Laura Ossorio Carballo, Renier A. L. van der Hoorn

Paste corresponding author name here
Email: renier.vanderhoorn@biology.ox.ac.uk

This PDF file includes:

SI Appendix, Figures S1 to S10
SI Appendix, Tables S1 to S6

SUPPLEMENTAL FIGURES

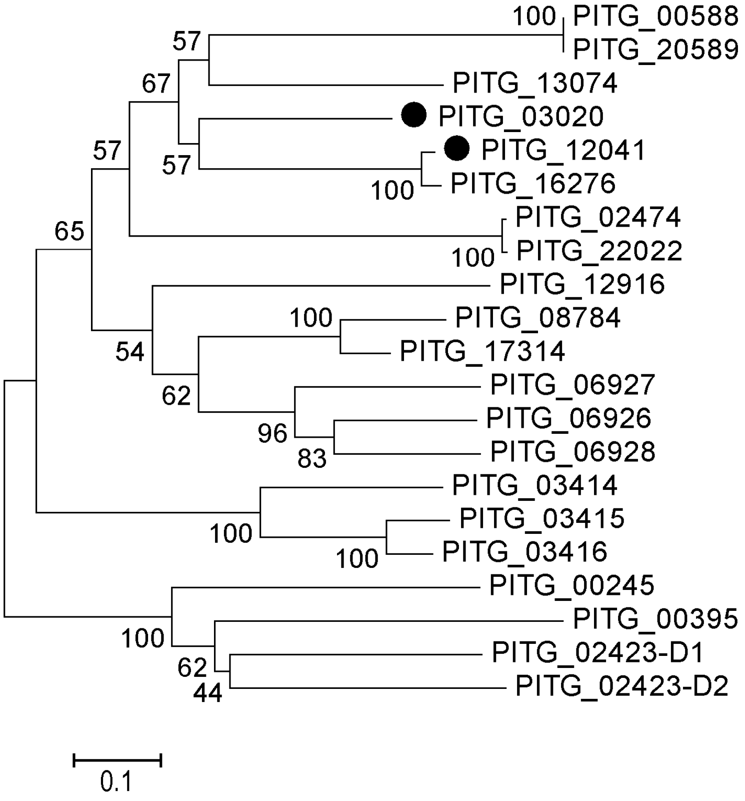

Figure S1 Phylogeny of all *P. infestans* PLCPs.

PITG_02423 contains two PLCP domains, referred to as D1 (domain 1) and D2 (domain 2), representing two distinct domains. All sequences are provided in Supplemental **File S1**.

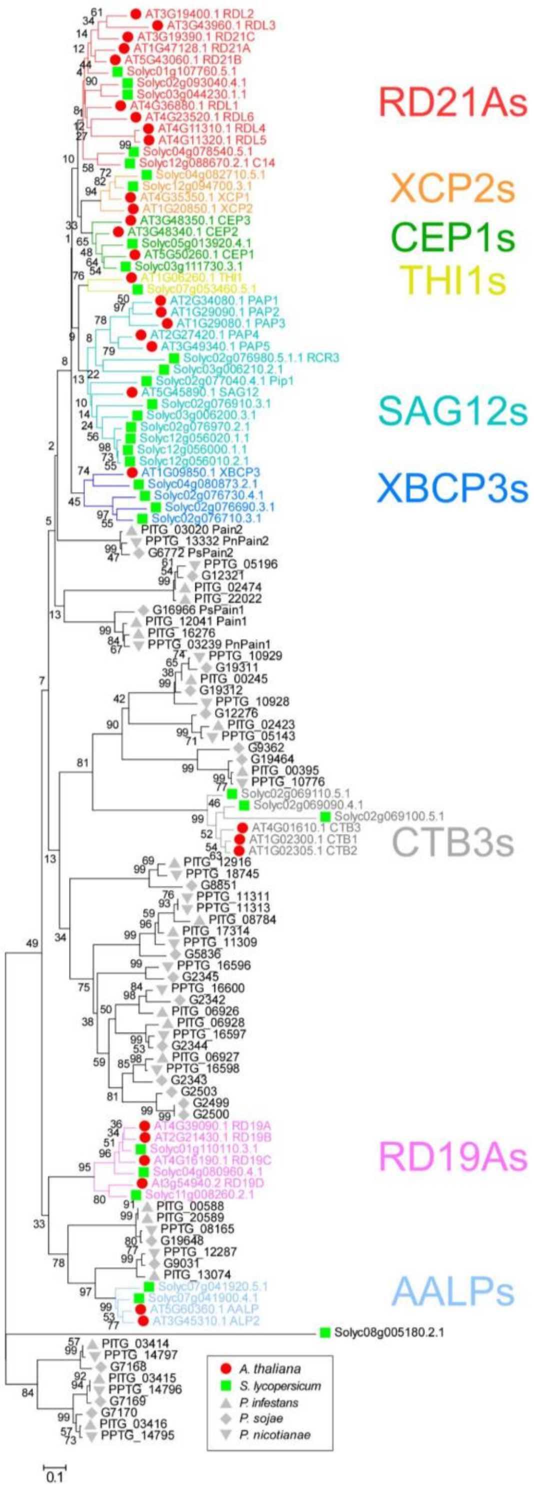

Figure S2 Phylogenetic analysis of PLCPs from Arabidopsis, tomato, *P. sojae*, *P. nicotianae* and *P. infestans*.

The evolutionary relationships among PLCPs were analyzed based on full-length protein sequences. All sequences used for phylogenetic reconstruction are listed in Supplemental **File S3**.

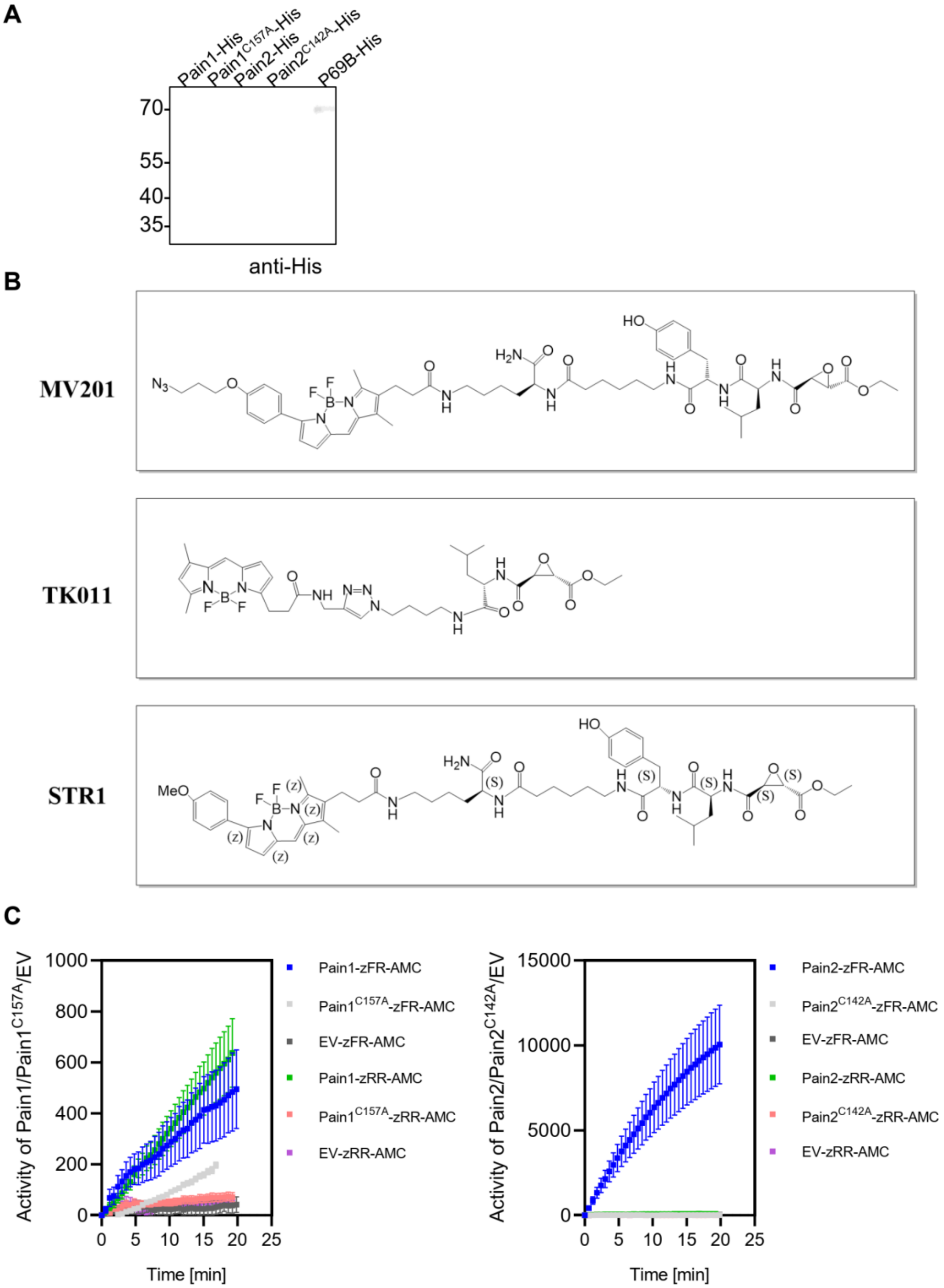
A
Pain1-His
Pain1C157A-His
Pain2-His
Pain2C142A-His
P69B-His
70
55
40
35
anti-His
B
MV201
TK011
STR1
C
Activity of Pain1/Pain1C157A/EV
Time [min]
Pain1-zFR-AMC
Pain1C157A-zFR-AMC
EV-zFR-AMC
Pain1-zRR-AMC
Pain1C157A-zRR-AMC
EV-zRR-AMC
Activity of Pain2/Pain2C142A/EV
Pain2-zFR-AMC
Pain2C142A-zFR-AMC
EV-zFR-AMC
Pain2-zRR-AMC
Pain2C142A-zRR-AMC
EV-zRR-AMC

Figure S3 Pain1 and Pain2 are distinct PLCPs.

(A) Detection of Pains and their catalytic mutants using anti-His antibody. P69B-His was used as a positive control (1). **(B)** Molecular structure of activity-based probe MV201 (2), TK011 (3) and STR1 probe for PLCPs. **(C)** Pain1 and Pain2 exhibit distinct substrate specificities toward zFR-AMC and zRR-AMC. Apoplastic fluid was isolated at 5 days post-infiltration (dpi) from *N. benthamiana* plants transiently expressing Pain1 or Pain2. Samples were diluted 16-fold and then incubated with 80 μM zFR-AMC or zRR-AMC substrates in the presence of 10 mM TCEP and 50 mM sodium acetate buffer (pH 5.0). Data represent the mean ± SE from nine replicates (n = 9).

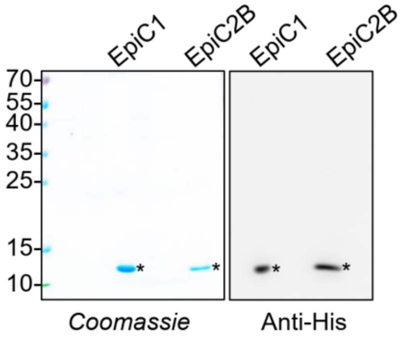

Figure S4 Detection of purified EpiC1 and EpiC2B proteins *in vitro*.

Coomassie staining and western blot detection of purified EpiC1 and EpiC2B proteins. White asterisks mark the positions of the target protein bands corresponding to the expected molecular weights.

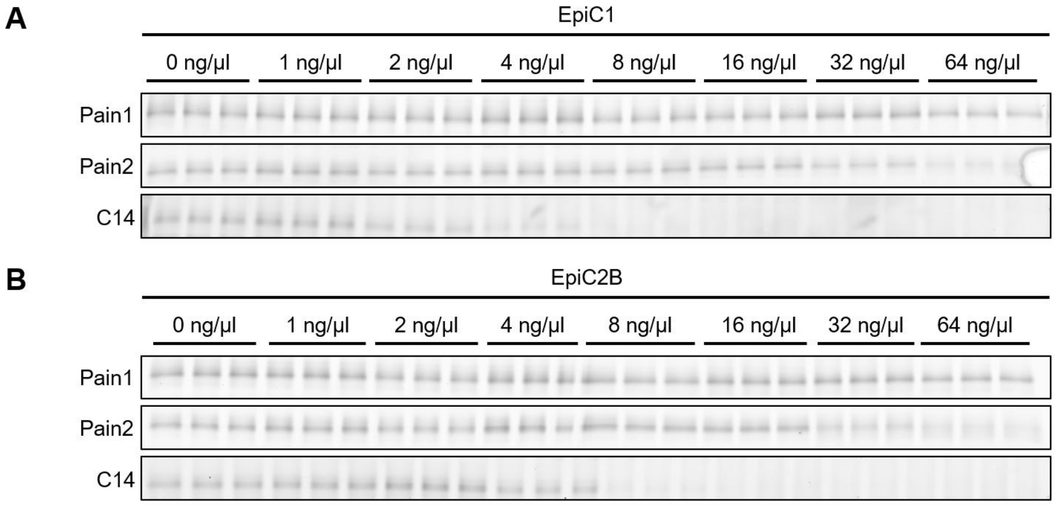

Figure S5 EpiCs preferentially inhibit C14 over Pains.

Apoplastic fluids from *N. benthamiana* leaves transiently overexpressing Pain1, Pain2, or C14 were preincubated for 30 minutes with different concentrations of EpiC1 **(A)** or EpiC2B **(B)**, followed by labelling with 2 µM TK011 probe for 3 hours. Labelled proteins were separated by SDS-PAGE and detected by fluorescence scanning. Consistent results were observed in three independent technical replicates.

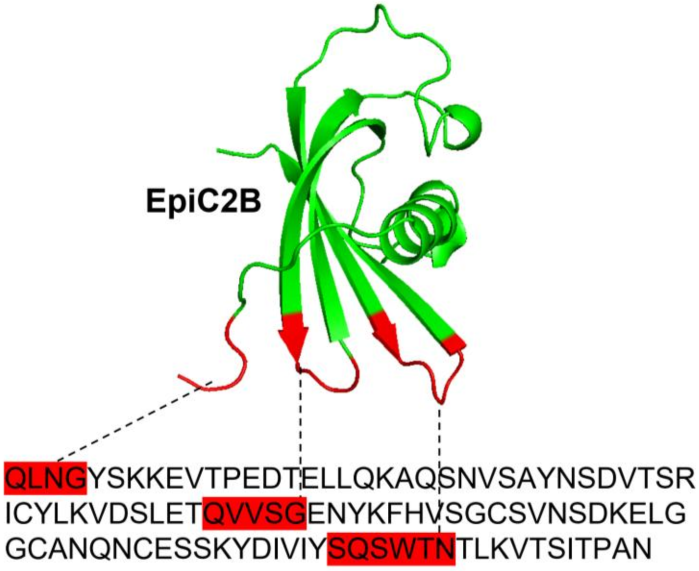

Figure S6 Inhibitory tripartite loops of EpiC2B.

The N-terminal (NT) loop (QLNG), the central loop containing the conserved QVVSG motif, and the C-terminal loop (SQSWTN), three key structural elements involved in protease inhibition, are highlighted in red.

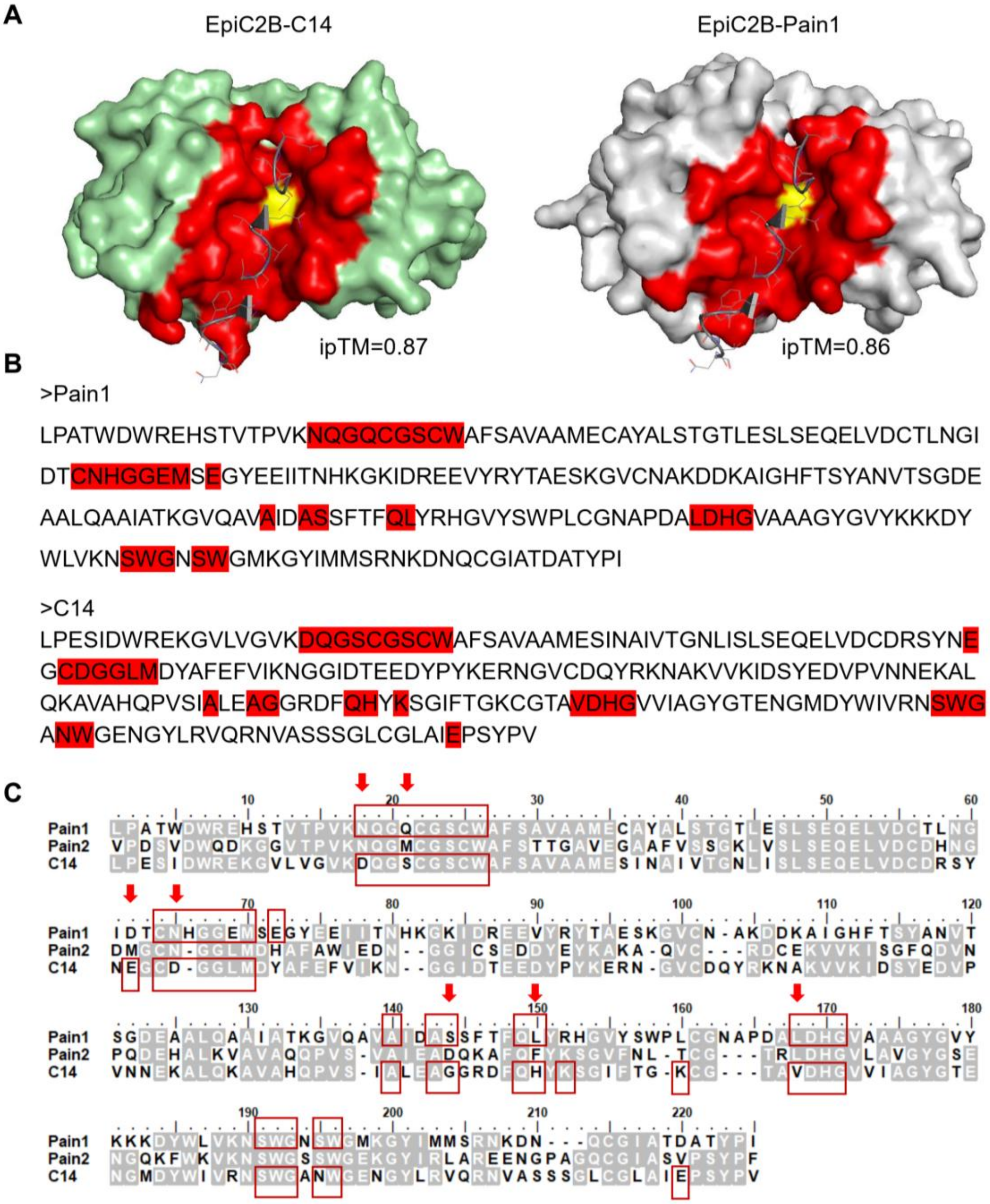

Figure S7 Mapping of the interface residues of EpiC2B with C14 and Pain1.

(A) Structural model of the EpiC2B-C14 and EpiC2B-Pain1 complex generated by AlphaFold3. The image shows the three loops of EpiC2B interacting with the substrate binding groove: the N-terminus (QLNG), the middle loop containing the QVVSG motif and the C-terminal loop (SQSWTN). The three surface-exposed loops of EpiC2B that interact with the substrate-binding groove of the proteases are highlighted in grey. The catalytic cysteine residue of Pain1 and C14 is highlighted in yellow, and all amino acid residues on the protease that are within 5 Å distance of the three loops of EpiC2B are highlighted in red. **(B)** Protein sequence of the catalytic domains of

Pain1 and C14. Residues that are within 5 Å distance of the inhibitory tripartite wedge of EpiC2B are highlighted in red. **(C)** Protein sequence alignment of the catalytic domains of Pain1, Pain2, and C14 proteases. Residues on Pain1 or C14 that are within 5 Å distance of the tripartite wedge of EpiC2B are boxed in red. The positions of seven amino acid residues, potentially crucial for inhibiting C14 protease activity, are indicated by red arrows.

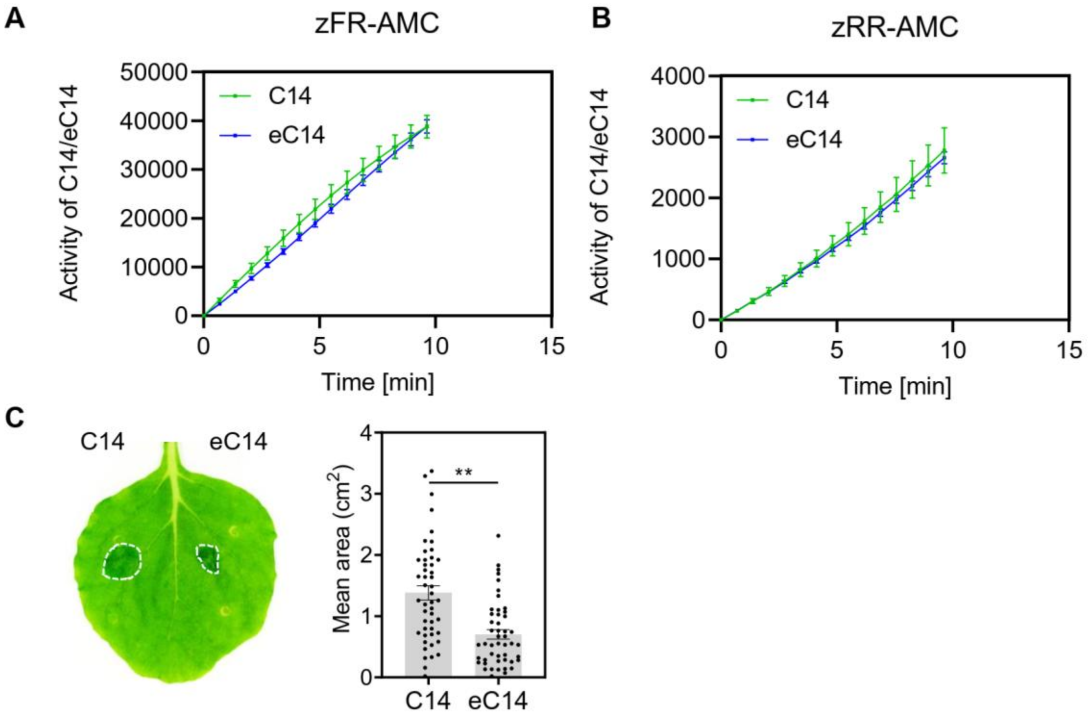

Figure S8 Engineering of C14 does not alter its substrate cleavage efficiency.

Apoplastic fluid was isolated at 5 dpi from *N. benthamiana* plants transiently expressing wild-type C14 and eC14. Samples were incubated with 80 μM zFR-AMC **(A)** or zRR-AMC **(B)** substrates in the presence of 10 mM TCEP and 50 mM sodium acetate buffer (pH 5.0). Data are presented as mean ± SE from nine measurements (n = 9), derived from three biological replicates, each with three technical replicates. **(C)** Overexpression of eC14 in *N. benthamiana* enhances resistance against *P. capsici*. The *N. benthamiana* leaves were agroinfiltrated with wild-type C14 or eC14, and 3-4 day after infiltration, the leaves were detached and inoculated with *P. capsici* strain LT263. Visible necrotic lesion areas are outlined with dashed lines and quantified. Each dot represents a biological replicate. Data are presented as mean ± standard error (P < 0.01, Student's t-test). The experiment was repeated four times with similar results.

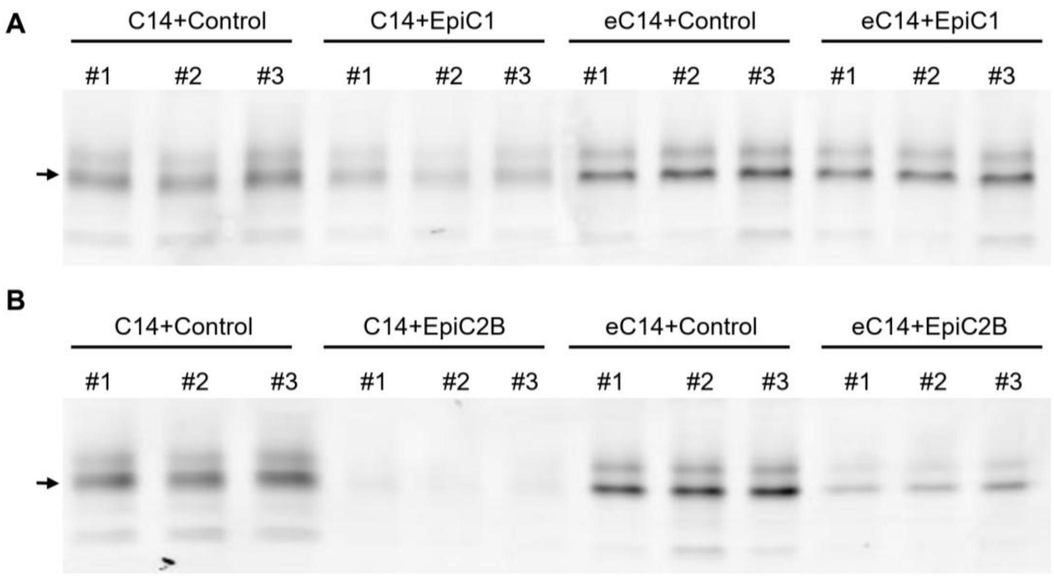

Figure S9 eC14 has reduced sensitivity to EpiC1 and EpiC2B.

Apoplastic fluids isolated from *N. benthamiana* leaves transiently expressing either wild-type C14 or eC14 were pre-incubated for 10 minutes with 50 ng of EpiC1 **(A)** and EpiC2B **(B)** in the presence of 5 mM TCEP, 50 mM Tris-HCl (pH 7.5) and 150 mM NaCl, followed by labeling with 2 µM STR1 for 30 minutes. The reaction buffer without EpiC1 and EpiC2B was used as a negative control. Samples were separated by SDS-PAGE and visualized by fluorescence scanning. Similar results were observed in at least three independent technical replicates.

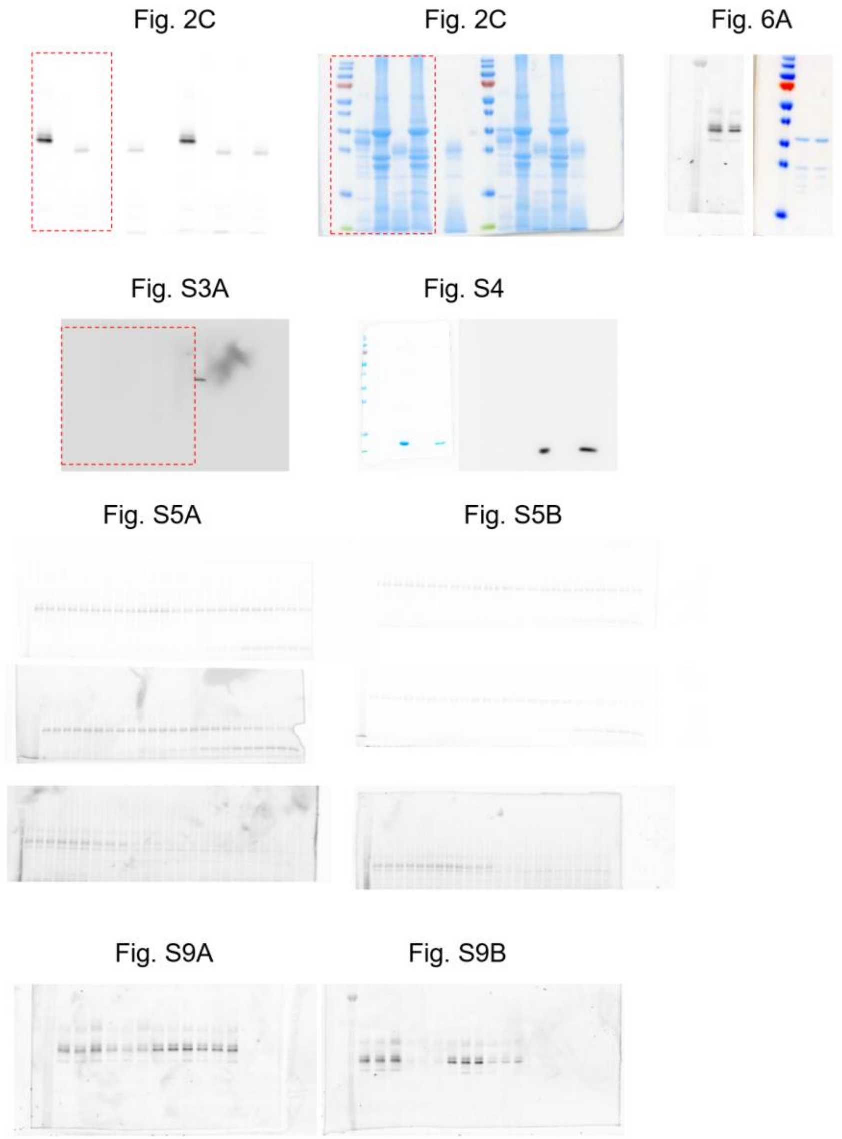

Figure S10 Original full-length images of ABPP, Coomassie staining and immunoblots. The figures associated with each gel image were indicated above the images. The regions used in the figures are highlighted with red rectangles.

SUPPLEMENTAL TABLES

Supplemental Table S1 Synthesised oligonucleotide sequences

>NtPR1-Pain1-His

TTACAATTATCGATACATGGGATTTGTTCTCTTTTCACAATTGCCTTCATTTCTTCTTGTCTCTACACTTCTCTTAT
TCCTAGTAATATCCCACTCTTGCCGTGCAGGAGGTCTAACAACCGATCTCCCATCGTCTCTGACTGCCTCGGAGCAG
AAGACGTGGGAAGCCTTCGTGGACTACGCTCTGGACTATGAGAAGAGCTATCGCAACGACGCCAACGACCACGACGT
GGTGCAGCTGCGTTTCCGATCCTTCGCGACCAATTTGGAGCGCATCCAGACGCACAACGAGGCCTACGAGCGTGGAG
AGCACAGCTTCACGCTGGGACTCAACGATCTCGCAGATCTGGCGGACGCCGAGTACAAGCAGCTTCTAAGCTACCGT
ACCAGAGACTCCAAGTCCTCGAGTGCATCTGAGACGTTCGTTAAACCTGAGAACGTTGAAGATCTACCAGCGACTTG
GGACTGGCGTGAGCATAGCACTGTCACACCCGTAAAGAACCAAGGCCAGTGCGGCTCGTGCTGGGCCTTCTCGGCCG
TTGCGGCCATGGAATGTGCCTACGCTCTGAGCACTGGCACGTTGGAGTCGCTCTCGGAGCAAGAGCTCGTGGACTGC
ACTCTTAACGGCATCGATACCTGCAACCATGGAGGAGAGATGAGTGAGGGATACGAAGAGATCATTACCAACCACAA
GGGCAAGATCGACCGCGAGGAGGACTATAGGTACACGGCCGAGTCTAAGGGCGTGTGCAACGCCAAGGACGATAAGG
CTATTGGCCACTTTACCTCGTACGCCAATGTGACGTCCGGCGATGAAGCTGCGCTACAGGCGGCCATTGCCACAAAG
GGTGTGCAGGCCGTGGCCATTGACGCCAGTAGCTTCACGTTCCAACTGTACCGCCACGGCGTGTACAGCTGGCCGCT
GTGCGGCAACGCCCCCGACGCGTTGGACCACGGCGTGGCTGCAGCTGGCTATGGCGTCTACAAGAAGAAGGACTATT
GGCTCGTTAAGAACTCGTGGGGCAACTCGTGGGGCATGAAGGGCTACATCATGATGAGCCGCAACAAAGACAACCAG
TGCGGCATCGCCACAGATGCCACGTACCCGATTATGACAAAGGAGGAAGTCGTCGAGGACCGTCCTATTGTTCTGGA
GACGACGGAGCTGGCCAGCATCATGGGTGGGCATCACCATCACCATCATTGAGCTTCTCTAGCTAGAGTCGA

>NtPR1-Pain2-His

TTACAATTATCGATACAATGGGATTTGTTCTCTTTTCACAATTGCCTTCATTTCTTCTTGTCTCTACACTTCTCTTA
TTCCTAGTAATATCCCACTCTTGCCGTGCAGGAGGTCTCAAGACGCCGCTTGAGTATGAGCATGAGTTCTCGGCCTG
GATGAAGACGCACTCCGTCTCGTTCTCGGACGCGCTCGAATTCGCTAAGCGTCTGGAGAACTACATCGCCAACGACA
TGTACATCATGGAACACAACCTCGAGAACGCCTGGACAGGCGTGAAGCTGGACCACAACGAGTTCTCCAGCATGTCG
TTCGAAGAGTTCAAGTTCAAAATGACGGGCTACGTCATGCCCGAGGGCTACTTGGAGCAGCGTCTGGCCTCCCGAGT
GGACAATCTCTGGAGCGACGTGCAGGTGCCTGACTCAGTGGACTGGCAAGACAAGGGAGGCGTGACCCCCGTCAAGA
ACCAGGGAATGTGTGGCTCGTGCTGGGCCTTCTCCACTACGGGTGCTGTAGAGGGTGCGGCCTTCGTCTCGAGCGGG
AAGCTGGTCAGTCTCTCGGAGCAGGAGCTGGTGGACTGCGACCATAATGGAGACATGGGCTGTAACGGAGGTCTCAT
GGACCACGCCTTTGCGTGGATTGAGGACAATGGAGGTATCTGTAGCGAGGATGACTACGAGTACAAGGCCAAGGCTC
AAGTCTGCCGCGATTGCGAGAAAGTTGTCAAGATCTCGGGTTTTCAGGACGTCAACCCGCAGGATGAACACGCACTC
AAGGTGGCGGTCGCTCAGCAGCCAGTTTCGGTGGCTATTGAGGCAGACCAGAAGGCGTTCCAGTTCTACAAGTCTGG
AGTCTTCAATCTCACATGCGGCACGAGACTGGACCATGGTGTGCTAGCTGTGGGCTATGGCTCGGAAAATGGACAGA
AATTTTGGAAGGTTAAGAACTCGTGGGGATCCTCATGGGGTGAGAAGGGGTACATCCGTCTTGCACGTGAGGAGAAC
GGACCTGCTGGACAGTGCGGCATCGCCTCGGTGCCTAGTTACCCGTTCGCGACGTTGATTAAAAAGGACGAAGAAAC
GGAGACCCAGAAGATTGTTGAGGAGCCGCGATCGGTCCCTGCTGCAAACGCCGTGGAGTCGTTCCCTGCCGAAGAAG
CACGCGACTTCCGTCCCGTGAACTTGGCTGATCTGTTCTCCTCCGCGAAGATTAAACAGTGTGGTGATGTTGGCAGC
GCGATCATTGACTTCTCCGACCTGGAAGTCACCCCGTCGTCCCCTCAACGCGGCCAGCCGGTTTCATTTTTCGGCAA
TGGGAACGCGAAGAAGGATTTCGACTCGGCGAACTTCAAGCTGGGCGTCAAGCTGGCCGGCACGCAGGTGTTCGGAC
ACTCGGGTAAGCTGTGCGGCGACACCCACATCCCTCTTCCGCTGGGCTTGGGCCACATCGATGTGCACGGCTTCGCG
TGCCCCATGAAAAAGGGCAAATCTTCGGACCTGAAGGTGGACGTGAACCTCCCAATCATTGCGCCTGCTGGCAACTA

CGAGATCCAGCTGACGAGCGATGACGACAGTAACAGCTCCTTGTTCTGCGTGAATGTTGAGCTTGACCTGACGGGTG<br>GCGAAACCGCGAAGAAGACCCACGTGTACGAACCCATTTCGTACATGGGTGGGCATCACCATCACCATCATTGAGCT<br>TCTCTAGCTAGAGTCGA
>eC14<br>TGGAGAGAAAAAGGTGTGCTTGTTGGTGTCAAGAATCAAGGACAATGTGGGAGTTGTTGGGCATTCTCTGCTGTTGC<br>TGCCATGGAATCAATAAACGCGATAGTCACTGGGAATTTGATATCACTATCAGAGCAAGAGTTGGTGGATTGTGATA<br>GGTCGTACAATGATGGTTGCAATGGTGGTCTTATGGACTACGCCTTTGAATTCGTCATTAAGAATGGAGGAATCGAC<br>ACTGAAGAGGACTACCCTTACAAAGAACGCAATGGCGTATGTGATCAATATAGGAAAAATGCCAAGGTTGTTAAAAT<br>AGATAGCTATGAAGATGTTCCTGTTAATAACGAAAAGGCGTTGCAAAAGGCTGTTGCACATCAACCTGTGAGCATTG<br>CACTTGAAGCTTCAGGCAGAGACTTCCAGCTTTACAAATCTGGTATCTTCACTGGAAAATGTGGTACTGCATTGGAT<br>CATGGTGTAGTTATTGCTGGATATGG

Highlighted are: the NtPR1 signal peptides (red), the GG linker regions (green), the His tag (grey), and catalytic cysteine (pink).

Supplemental Table S2 The sequence of pJK590#07_SP vector.

>pJK590#07_SP

GAACACTCTGTGCCGAATTCGGATCCAGCGGTCCTGCTGAGCCTCGACATGTTGTCGCAAAATTCGCCCTGGACCCG
CCCAACGATTTGTCGTCACTGTCAAGGTTTGACCTGCACTTCATTTGGGGCCCACATACACCAAAAAAATGCTGCAT
AATTCTCGGGGCAGCAAGTCGGTTACCCGGCCGCCGTGCTGGACCGGGTTGAATGGTGCCCGTAACTTTCGGTAGAG
CGGACGGCCAATACTCAACTTCAAGGAATCTCACCCATGCGCGCCGGCGGGGAACCGGAGTTCCCTTCAGTGAGCGT
TATTAGTTCGCCGCTCGGTGTGTCGTAGATACTAGCCCCTGGGGCACTTTTGAAATTTGAATAAGATTTATGTAATC
AGTCTTTTAGGTTTGACCGGTTCTGCCGCTTTTTTTAAAATTGGATTTGTAATAATAAAACGCAATTGTTTGTTATT
GTGGCGCTCTATCATAGATGTCGCTATAAACCTATTCAGCACAATATATTGTTTTCATTTTAATATTGTACATATAA
GTAGTAGGGTACAATCAGTAAATTGAACGGAGAATATTATTCATAAAAATACGATAGTAACGGGTGATATATTCATT
AGAATGAACCGAAACCGGCGGTAAGGATCTGAGCTACACATGCTCAGGTTTTTTACAACGTGCACAACAGAATTGAA
AGCAAATATCATGCGATCATAGGCTTCTCGCATATCTCATTAAAGCAGGACAAGCTTACTCGCTTTCTTTTTCGAAA
GTTTGAGTACCTTCAGGGCATCCTCTTGATACATTACTTTCCACTTCGATTGGGGCAAGCTGTAGCAGTTCTTGCTT
AGACCGAATTGCCATCTCACAGAGATGCTGAAGAGTTCGCGACCCTCCAGAAACGGTGATACTAACTCCTCGAAACC
GAATACTATAGGTACATCCGATCTGGTCGAAACCGAAAAATCGAGATGCTGCATAGTTAACCGAATCTCCCGTCCAA
GATCCAAGGACTCTGTGCAGTGAAGCTTCCGTCCTGTCGTATCTGAGATATCTCTTAAATACAACTTTCCCGAAACC
CCAGCTTTCCTTGAAACCAAGGGGATTATCTTGATTCGAATTCGTCTCATCGTTATGTAGCCGCCACTCAGTCCAAC
TCGGACTTTCGTCAGGAAGTTTGAAGGGAGAAGTGGTACCTCCTGATCCTCCATCCCAACGTTCACTGTTAGCTTGT
TCCCTAGCGTCGTTTCCTTGTATAGCTCGTTCCATCATTCGATTTGGTGTATCGAGATTGGTTATGAAATTCAGATG
CTAGTGTAATGTATTGGTAATTTGGGAAGATATAATAGGAAGCAAGGCTATTTATCCATTTCTGAAAAGGCGAAATG
GCGTCACCGCGAGCGTCACGCGCATTCCGTTCTTGCTGTAAAGCGTTGTTTGGTACACTTTTGACTAGCGAGGCTTG
GCGTGTCAGCGTATCTATTCAAAAGTCGTTAATGGCTGCGGATCAAGAAAAAGTTGGAATAGAAACAGAATACCCGC
GAAATTCAGGCCCGGTTGCCATGTCCTACACGCCGAAATAAACGACCAAATTAGTAGAAAAATAAAAACTGACTAGG
ATACTTACGTCACGTCTTGCGCACTGATTTGAAAAATCTCAATATAAACTCCGCAAGAATTCAAGCTTGGAGGTCAA
CATGGTGGAGCACGACACTCTGGTCTACTCCAAAAATGTCAAAGATACAGTCTCAGAAGATCAAAGGGCTATTGAGA
CTTTTCAACAAAGGATAATTTCGGGAAACCTCCTCGGATTCCATTGCCCAGCTATCTGTCACTTCATCGAAAGGACA
GTAGAAAAGGAAGGTGGCTCCTACAAATGCCATCATTGCGATAAAGGAAAGGCTATCATTCAAGATCTCTCTGCCGA
CAGTGGTCCCAAAGATGGACCCCCACCCACGAGGAGCATCGTGGAAAAAGAAGAGGTTCCAACCACGTCTACAAAGC
AAGTGGATTGATGTGATAACATGGTGGAGCACGACACTCTGGTCTACTCCAAAAATGTCAAAGATACAGTCTCAGAA
GATCAAAGGGCTATTGAGACTTTTCAACAAAGGATAATTTCGGGAAACCTCCTCGGATTCCATTGCCCAGCTATCTG
TCACTTCATCGAAAGGACAGTAGAAAAGGAAGGTGGCTCCTACAAATGCCATCATTGCGATAAAGGAAAGGCTATCA
TTCAAGATCTCTCTGCCGACAGTGGTCCCAAAGATGGACCCCCACCCACGAGGAGCATCGTGGAAAAAGAAGAGGTT
CCAACCACGTCTACAAAGCAAGTGGATTGATGTGACATCTCCACTGACGTAAGGGATGACGCACAATCCCACTATCC
TTCGCAAGACCCTTCCTCTATATAAGGAAGTTCATTTCATTTGGAGAGGACACGCTCGAGTATAAGAGCTCATTTTT
ACAACAATTACCAACAACAACAAACAACAAACAACATTACAATTACATTTACAATTATCGATACAATGGCAGCTCAC
AGCTCAACTCTCACCATATCCATACTTCTCATGCTCATCTTCTCCACCTTATCCTCTGCTTCCGATATGTCAATTAT
TAGCTACGACGAGACACATATTCACCGTCGTACCGACGATGAAGTCTCGGCGTTGTACGAGTCCTGGCTAATCGAGC
ACGGAAAATCGTACAACGCCTTAGGAGAAAAGGATAAGAGATTTCAGATCTTCAAGGATAACTTGAGATACATAGAC
GAACAGAACTCTGTTCCGAATCAGAGTTATAAGCTCGGATTGACGAAATTCGCCGATCTGACTAATGAGGAGTACAG
GTCGATTTACTTGGGAACGAAGAGTTCCGGTGACCGGAAAAAGTTATCGAAGAACAAAAGTGATCGGTATCTTCCTA
AAGTTGGGGATAGCTTGCCGGAATCAATTGACTGGAGAGAAAAAGGTGTGCTTGTTGGTGTCAAGGATCAAGGAAGC

TGTGGGAGTTGTTGGGCATTCTCTGCTGTTGCTGCCATGGAATCAATAAACGCGATAGTCACTGGGAATTTGATATC
ACTATCAGAGCAAGAGTTGGTGGATTGTGATAGGTCGTACAATGAAGGTTGCGATGGTGGTCTTATGGACTACGCCT
TTGAATTCGTCATTAAGAATGGAGGAATCGACACTGAAGAGGACTACCCTTACAAAGAACGCAATGGCGTATGTGAT
CAATATAGGAAAAATGCCAAGGTTGTTAAAATAGATAGCTATGAAGATGTTCCTGTTAATAACGAAAAGGCGTTGCA
AAAGGCTGTTGCACATCAACCTGTGAGCATTGCACTTGAAGCTGGTGGCAGAGACTTCCAGCACTACAAATCTGGTA
TCTTCACTGGAAAATGTGGTACTGCAGTGGATCATGGTGTAGTTATTGCTGGATATGGTACTGAGAATGGCATGGAT
TATTGGATCGTTAGGAACTCATGGGGAGCTAACTGGGGAGAGAACGGCTACCTCAGAGTCCAGCGTAACGTTGCCAG
CTCTAGTGGCTTGTGTGGTTTAGCCATAGAGCCTTCATATCCAGTAAAAACAGGACCAAATCCTCCTAAACCCGCTC
CATCTCCTCCATCTCCGGTCAAGCCACCTACAGAGTGTGATGAATATTCTCAATGCGCTGTCGGCACCACTTGCTGC
TGTATCCTTCAGTTCCGTAGGTCTTGCTTCTCTTGGGGATGCTGCCCACTTGAAGGAGCCACTTGCTGTGAGGACCA
CTACAGTTGCTGCCCACACGACTATCCTATCTGCAATGTTCGTCAAGGAACATGCTCAATGAGCAAGGGCAACCCAC
TGGGAGTGAAGGCAATGAAGCGCATTCTTGCACAACCTATTGGGGCCTTCGGAAATGGAGGAAAGAAGAGCAGTTCT
TGAGCTTCTCTAGCTAGAGTCGATCGACAAGCTCGAGTTTCTCCATAATAATGTGTGAGTAGTTCCCAGATAAGGGA
ATTAGGGTTCCTATAGGGTTTCGCTCATGTGTTGAGCATATAAGAAACCCTTAGTATGTATTTGTATTTGTAAAATA
CTTCTATCAATAAAATTTCTAATTCCTAAAACCAAAATCCAGTACTAAAATCCAGATCGCTACTAGAGGATGCACAT
GTGACCGAGGGACACGAAGTGATCCGTTTAAACTATCAGTGTTTGACAGGATATATTGGCGGGTAAACCTAAGAGAA
AAGAGCGTTTATTAGAATAATCGGATATTTAAAAGGGCGTGAAAAGGTTTATCCGTTCGTCCATTTGTATGTGCATG
CCAACCACAGGGTTCCCCTCGGGAGTCAGCCGTGCGGCTGCATGAAATCCTGGCCGGTTTGTCTGATGCCAAGCTGG
CGGCCTGGCCGGCCAGCTTGGCCGCTGAAGAAACCGAGCGCCGCCGTCTAAAAAGGTGATGTGTATTTGAGTAAAAC
AGCTTGCGTCATGCGGTCGCTGCGTATATGATGCGATGAGTAAATAAACAAATACGCAAGGGGAACGCATGAAGGTT
ATCGCTGTACTTAACCAGAAAGGCGGGTCAGGCAAGACGACCATCGCAACCCATCTAGCCCGCGCCCTGCAACTCGC
CGGGGCCGATGTTCTGTTAGTCGATTCCGATCCCCAGGGCAGTGCCCGCGATTGGGCGGCCGTGCGGGAAGATCAAC
CGCTAACCGTTGTCGGCATCGACCGCCCGACGATTGACCGCGACGTGAAGGCCATCGGCCGGCGCGACTTCGTAGTG
ATCGACGGAGCGCCCCAGGCGGCGGACTTGGCTGTGTCCGCGATCAAGGCAGCCGACTTCGTGCTGATTCCGGTGCA
GCCAAGCCCTTACGACATATGGGCCACCGCCGACCTGGTGGAGCTGGTTAAGCAGCGCATTGAGGTCACGGATGGAA
GGCTACAAGCGGCCTTTGTCGTGTCGCGGGCGATCAAAGGCACGCGCATCGGCGGTGAGGTTGCCGAGGCGCTGGCC
GGGTACGAGCTGCCCATTCTTGAGTCCCGTATCACGCAGCGCGTGAGCTACCCAGGCACTGCCGCCGCCGGCACAAC
CGTTCTTGAATCAGAACCCGAGGGCGACGCTGCCCGCGAGGTCCAGGCGCTGGCCGCTGAAATTAAATCAAAACTCA
TTTGAGTTAATGAGGTAAAGAGAAAATGAGCAAAAGCACAAACACGCTAAGTGCCGGCCGTCCGAGCGCACGCAGCA
GCAAGGCTGCAACGTTGGCCAGCCTGGCAGACACGCCAGCCATGAAGCGGGTCAACTTTCAGTTGCCGGCGGAGGAT
CACACCAAGCTGAAGATGTACGCGGTACGCCAAGGCAAGACCATTACCGAGCTGCTATCTGAATACATCGCGCAGCT
ACCAGAGTAAATGAGCAAATGAATAAATGAGTAGATGAATTTTAGCGGCTAAAGGAGGCGGCATGGAAAATCAAGAA
CAACCAGGCACCGACGCCGTGGAATGCCCCATGTGTGGAGGAACGGGCGGTTGGCCAGGCGTAAGCGGCTGGGTTGT
CTGCCGGCCCTGCAATGGCACTGGAACCCCCAAGCCCGAGGAATCGGCGTGACGGTCGCAAACCATCCGGCCCGGTA
CAAATCGGCGCGGCGCTGGGTGATGACCTGGTGGAGAAGTTGAAGGCCGCGCAGGCCGCCCAGCGGCAACGCATCGA
GGCAGAAGCACGCCCCGGTGAATCGTGGCAAGCGGCCGCTGATCGAATCCGCAAAGAATCCCGGCAACCGCCGGCAG
CCGGTGCGCCGTCGATTAGGAAGCCGCCCAAGGGCGACGAGCAACCAGATTTTTTCGTTCCGATGCTCTATGACGTG
GGCACCCGCGATAGTCGCAGCATCATGGACGTGGCCGTTTTCCGTCTGTCGAAGCGTGACCGACGAGCTGGCGAGGT
GATCCGCTACGAGCTTCCAGACGGGCACGTAGAGGTTTCCGCAGGGCCGGCCGGCATGGCCAGTGTGTGGGATTACG
ACCTGGTACTGATGGCGGTTTCCCATCTAACCGAATCCATGAACCGATACCGGGAAGGGAAGGGAGACAAGCCCGGC
CGCGTGTTCCGTCCACACGTTGCGGACGTACTCAAGTTCTGCCGGCGAGCCGATGGCGGAAAGCAGAAAGACGACCT
GGTAGAAACCTGCATTCGGTTAAACACCACGCACGTTGCCATGCAGCGTACGAAGAAGGCCAAGAACGGCCGCCTGG

TGACGGTATCCGAGGGTGAAGCCTTGATTAGCCGCTACAAGATCGTAAAGAGCGAAACCGGGCGGCCGGAGTACATC
GAGATCGAGCTAGCTGATTGGATGTACCGCGAGATCACAGAAGGCAAGAACCCGGACGTGCTGACGGTTCACCCCGA
TTACTTTTTGATCGATCCCGGCATCGGCCGTTTTCTCTACCGCCTGGCACGCCGCGCCGCAGGCAAGGCAGAAGCCA
GATGGTTGTTCAAGACGATCTACGAACGCAGTGGCAGCGCCGGAGAGTTCAAGAAGTTCTGTTTCACCGTGCGCAAG
CTGATCGGGTCAAATGACCTGCCGGAGTACGATTTGAAGGAGGAGGCGGGGCAGGCTGGCCCGATCCTAGTCATGCG
CTACCGCAACCTGATCGAGGGCGAAGCATCCGCCGGTTCCTAATGTACGGAGCAGATGCTAGGGCAAATTGCCCTAG
CAGGGGAAAAAGGTCGAAAAAGCTCTTTCCTGTGGATAGCACGTACATTGGGAACCCAAAGCCGTACATTGGGAAC
CGGAACCCGTACATTGGGAACCCAAAGCCGTACATTGGGAACCGGTCACACATGTAAGTGACTGATATAAAAGAGAA
AAAAGGCGATTTTTCCGCCTAAAACTCTTTAAAACTTATTAAAACTCTTAAAACCCGCCTGGCCTGTGCATAACTGT
CTGGCCAGCGCACAGCCGAACAGCTGCAAAAAGCGCCTACCCTTCGGTCGCTGCGCTCCCTACGCCCCGCCGCTTCG
CGTCGGCCTATCGCGGCCGCTGGCCGCTCAAAAATGGCTGGCCTACGGCCAGGCAATCTACCAGGGCGCGGACAAGC
CGCGCCGTCGCCACTCGACCGCCGGCGCCCACATCAAGGCTCCGAGTGCGCGGAACCCCTATTTGTTTATTTTTCTA
AATACATTCAAATATGTATCCGCTCATGAGACAATAACCCTGATAAATGCTTCAATAATATTGAAAAAGGAAGAGTA
TGGCTAAAATGAGAATATCACCGGAATTGAAAAAACTGATCGAAAAATACCGCTGCGTAAAAGATACGGAAGGAATG
TCTCCTGCTAAGGTATATAAGCTGGTGGGAGAAAATGAAAACCTATATTTAAAAATGACGGACAGCCGGTATAAAGG
GACCACCTATGATGTGGAACGGGAAAAGGACATGATGCTATGGCTGGAAGGAAAGCTGCCTGTTCCAAAGGTCCTGC
ACTTTGAACGGCATGATGGCTGGAGCAATCTGCTCATGAGTGAGGCCGATGGCGTCCTTTGCTCGGAAGAGTATGAA
GATGAACAAAGCCCTGAAAAGATTATCGAGCTGTATGCGGAGTGCATCAGGCTCTTTCACTCCATCGACATATCGGA
TTGTCCCTATACGAATAGCTTAGACAGCCGCTTAGCCGAATTGGATTACTTACTGAATAACGATCTGGCCGATGTGG
ATTGCGAAAACTGGGAAGAGGACACTCCATTTAAAGATCCGCGCGAGCTGTATGATTTTTTAAAGACGGAAAAGCCC
GAAGAGGAACTTGTCTTTTCCCACGGCGACCTGGGAGACAGCAACATCTTTGTGAAAGATGGCAAAGTAAGTGGCTT
TATTGATCTTGGGAGAAGCGGCAGGGCGGACAAGTGGTATGACATTGCCTTCTGCGTCCGGTCGCTCAGGGAGGATA
TCGGGGAAGAACAGTATGTCGAGCTATTTTTTGACTTACTGGGGATCAAGCCTGATTGGGAGAAAATAAAATATTAT
ATTTTACTGGATGAATTGTTTTAGCTGTCAGACCAAGTTTACTCATATATACTTTAGATTGATTTAAAACTTCATTT
TTAATTTAAAAGGATCTAGGTGAAGATCCTTTTTGATAATCTCATGACCAAAATCCCTTAACGTGAGTTTTCGTTCC
ACTGAGCGTCAGACCCCGTAGAAAAGATCAAAGGATCTTCTTGAGATCCTTTTTTTCTGCGCGTAATCTGCTGCTTG
CAAACAAAAAAACCACCGCTACCAGCGGTGGTTTGTTTGCCGGATCAAGAGCTACCAACTCTTTTTCCGAAGGTAAC
TGGCTTCAGCAGAGCGCAGATACCAAATACTGTTCTTCTAGTGTAGCCGTAGTTAGGCCACCACTTCAAGAACTCTG
TAGCACCGCCTACATACCTCGCTCTGCTAATCCTGTTACCAGTGGCTGCTGCCAGTGGCGATAAGTCGTGTCTTACC
GGGTTGGACTCAAGACGATAGTTACCGGATAAGGCGCAGCGGTCGGGCTGAACGGGGGGTTCGTGCACACAGCCCAG
CTTGGAGCGAACGACCTACACCGAACTGAGATACCTACAGCGTGAGCTATGAGAAAGCGCCACGCTTCCCGAAGGGA
GAAAGGCGGACAGGTATCCGGTAAGCGGCAGGGTCGGAACAGGAGAGCGCACGAGGGAGCTTCCAGGGGGAAACGCC
TGGTATCTTTATAGTCCTGTCGGGTTTCGCCACCTCTGACTTGAGCGTCGATTTTTGTGATGCTCGTCAGGGGGGCG
GAGCCTATGGAAAAACGCCAGCAACGCGGCCTTTTTACGGTTCCTGCTCGGATCTGTTGGACCGGACAGTAGTCATG
GTTGATGGGCTGCCTGTATCGAGTGGTGATTTTGTGCCGAGCTGCCGGTCGGGGAGCTGTTGGCTGGCTGGTGGCAG
GATATATTGTGGTGTAAACAAATTGACGCTTAGACAACTTAATAACACATTGCGGACGTTTTTAATGTACTGGGGTT

Highlighted is the sequence encoding C14 (yellow).

Supplementary Table S3 Used plasmids

Plasmid	Description	Reference
pJK187	Binary vector (pL0V2-2x35S::SC)	Homma et al., 2023
P19	Binary vector carrying p19 silencing inhibitor	Van der Hoorn et al., 2003
pAJVP001	Binary vector for Pain1 expression	This work
pHJ014	Binary vector for Pain2 expression	This work
pAJVP006	Binary vector for Pain1^{C157A} expression	This work
pAJVP007	Binary vector for Pain2^{C142A} expression	This work
pJK590#07_SP	Binary vector for C14 expression	Kourelis J, et al. 2020
pHJ143	Binary vector for eC14 expression	This work
pJK155	Bacterial expression EpiC1	Chen C, et al. 2024
pJK157	Bacterial expression EpiC2B	Schuster M, et al. 2024

Supplementary Table S4 Used primer sequences

Primers	Nucleotide sequence (5'-3')	Use
Pain1-C157A-His-F1	TTACAATTATCGATACAATGGGATTTGTTCTCTTTTC	Cloning
Pain1-C157A-His-R1	GCAACGGCCGAGAAGGCCCAGGCCGAGCCGCACTGGCCTT	Cloning
Pain1-C157A-His-F2	TGGGCCTTCTCGGCCGTTGC	Cloning
Pain1-C157A-His-R2	TCTAGCTAGAGAAGTCAATGATGGTGATGGTGATG	Cloning
Pain2-C142A-His-F1	TTACAATTATCGATACAATGGGATTTGTTCTCTTTTC	Cloning
Pain2-C142A-His-R1	CCCGTAGTGGAGAAGGCCCAGGCCGAGCCACACATTCCCT	Cloning
Pain2-C142A-His-F2	TGGGCCTTCTCCACTACGGG	Cloning
Pain2-C142A-His-R2	TCTAGCTAGAGAAGTCAATGATGGTGATGGTGATG	Cloning
pJK187-F	CTATCCTTCGCAAGACCCTTC	Sequencing
pJK187-R	CTCAACACATGAGCGAAACC	Sequencing
pJK590#07_SP-linearized-F	GGTGTAGTTATTGCTGGATATGG	Cloning
pJK590#07_SP-linearized-R	CAAGCACACCTTTTTCTCTCCA	Cloning

Supplementary Table S5 Average expression levels (in FPKM) of 20 *P. infestans* PLCPs during infection. Data extracted from (7), shown as mean of n=3 replicates.

Gene ID	1dpi	2dpi	3dpi
PITG_00245	8.440117	2.393031	1.520397
PITG_00395	44.86291	70.72413	85.67472
PITG_00588	30.60442	7.943414	56.42791
PITG_02423	65.9595	54.02947	134.3779
PITG_02474	0	0	0
PITG_03020	151.2096	105.8548	313.394
PITG_03414	0	5.918245	13.6343
PITG_03415	78.43348	58.50445	33.4248
PITG_03416	29.47411	30.07621	35.0264
PITG_06926	0	1.710194	0.373221
PITG_06927	0	0	0.368515
PITG_06928	0	0	1.729921
PITG_08784	0	0	0
PITG_12041	61.68263	111.4389	271.6172
PITG_12916	0	21.60548	7.568051
PITG_13074	3.965012	3.299544	41.28541
PITG_16276	0	1.56865	4.230191
PITG_17314	0	0	0
PITG_20589	21.03038	27.39053	33.87398
PITG_22022	0	0	0

Supplementary Table S6 Expression levels (FPKM) of Pains and EpiCs during infection. Data extracted from (7).

Gene	Pain1			
1 dpi	107.4663	81.42396	20.75791	37.08234
2 dpi	142.0263	93.51102	111.5987	98.61942
3 dpi	218.8857	293.1271	279.4435	295.0123
**Gene**	**Pain2**			
1 dpi	251.2738	151.7968	120.8436	80.92426
2 dpi	60.4524	132.1431	114.896	115.9277
3 dpi	318.8471	322.734	319.5267	292.4683
**Gene**	**EpiC1**			
1 dpi	29.28759	38.82264	0	39.04139
2 dpi	76.90038	86.99335	65.01476	66.12439
3 dpi	7.22654	24.86371	7.073118	18.75362
**Gene**	**EpiC2B**			
1 dpi	994.4756	938.3188	840.9429	976.0881
2 dpi	380.2146	690.4698	789.9913	731.1857
3 dpi	291.6396	243.5666	218.5434	308.5265

References

1. F. Homma, J. Huang, R. A. L. van der Hoorn, AlphaFold-multimer predicts cross-kingdom interactions at the plant-pathogen interface. Nat. Commun. 14, 6040 (2023)
2. K. H. Richau et al., Subclassification and biochemical analysis of plant papain-like cysteine proteases displays subfamily-specific characteristics. Plant Physiol. 158, 1583–1599 (2012).
3. M. Schuster et al., Enhanced late blight resistance by engineering an EpiC2B-insensitive immune protease. Plant Biotechnol. J. 22, 284–286 (2024).
4. R. A. Van Der Hoorn, S. Rivas, B. B. Wulff, J. D. Jones, M. H. Joosten, Rapid migration in gel filtration of the Cf-4 and Cf-9 resistance proteins is an intrinsic property of Cf proteins and not because of their association with high-molecular-weight proteins. Plant J. 35, 305–315 (2003)
5. J. Kourelis et al., Evolution of a guarded decoy protease and its receptor in solanaceous plants. Nat. Commun. 11, 4393 (2020)
6. C. Chen et al., Extracellular plant subtilases dampen cold-shock peptide elicitor levels. Nat. Plants 10, 1749–1760 (2024)
7. J. Huang et al., Phytophthora effectors modulate genome-wide alternative splicing of host mRNAs to reprogram plant immunity. Mol. Plant. 13, 1470–1484 (2020)
