# Supplementary material for: Pathogen-inspired engineering of plant protease enhances late blight resistance
Source: Proc Natl Acad Sci U S A. 2026 Jan 9;123(2):e2524700123. doi: 10.1073/pnas.2524700123 (PMC12799129; doi:10.1073/pnas.2524700123)
Supplement: Supplementary file 2 — Dataset S01 (PDF) [file pnas.2524700123.sd01.pdf]

Supplemental File S1: Protein sequences of 20 *P. infestans* PLCPs used for the Figure 1.

The predicted signal peptides by SignalP - 6.0 and TargetP - 2.0 are highlighted in red, and the catalytic domains are shown in green. The inhibitor domains of Pain1 and Pain2 are highlighted in yellow, and the C-terminal ML domain of Pain2 is highlighted in grey.

>PITG_00245

MAITDKSDLPLLQPRISPLLDVNSVSQSSTSPLQSIWENFHADRFKLRSIGSVLMWIALGS
FVSSAVLAPKKLVDYTHNGVSMHAVQDQLDLMAACPPDGKGEGDVDVVQTNKGAGWF
LGGNDPEHVLSPLPEVTSLDDFPERWDWRDYNKTGISLTTSVMNQMVPRACGSCWAF
ATVSALSDRIRIARFKTTGRLDTEVLLSPQVLLDCGMRSFGSCHGGDPRYAHKWIHENGI
VDLTCNPYIASHPSWMGRGDCAATQCHTCNLKGECFVLEDPIKYRISEYGTLNFATSEEF
QLQAMNEIYHRGPIVVSMYSLSPEYRQYKGGYILRDSTKYPGTTHVVSLVGWGTDVKTG
VKYWIVRNSDGTNWGDRGFFLAERGVNIYNMESHGAWAVPIV

>PITG_00395

MKGREHLHPKVHSLPTMSLDEAQELEQLPKHLDWCERGFCVPSWNQHIPQYCGSCFA
HGAMSSAQDRIKIANTRRKYTGADVMLGRQSFLNCAPGHGLSAGCDGGEASDVYEFM
RLYGLPDESCLPYNATDHTKYQATNGTCPPEGYCMNCMYTPESKKVPQCFPVTKMVRY
RAKSHGHLSGELAMMKEIMEDGPITCGIACSEEFTYKYKAGILEDKTGFMDIDHDVEIVG
WGEEDGVKYWHIRNSWGTYWGMNGFFKIVRGKNNLGIEADCAFMRPDISDEELVWEE
KAVYGGSIFGIVPFKKSAKDHPIKDTSEDVTRPDGEVLAAHYTESFATETEAHHQDRSFA
TLAVTFFVSGCVCAALAAVIVLKFRGHRYVYRTIA

>PITG_00588

MGKKKSKTTLPSEDHEASASESLLSSSDVRVHHLERHESAVIRRQRSKSRRIWCAIIALT
TGILGFIAVLAILRAVEDASHLAPTKRPVFPTQYEASVTFHMPYMDMVEPLYVHVDETKG
LQKLSYYGGTDVYIYNTSGTSYQIIPVIRERKCFKSGSESLQHIFPNMTLFEPQHGVFLVE
GRPCFSWKFVTKLHEPTEDGLLGEYTLYVDQKTERPVRFHYVGRNGMLGGSHIDEYFL
DYIYVREGPVDEDVFSFLPSSMNCTEMPGDDGGPSRIPKQDIHMLMPEGSTVKKEIFDN
FSATHEKEYNDDAEAVQRMATFHHNLRFINGENRKGLPYHLEVNKFADLSYEERRALHR
PSRVKRAKDNKAMAVHELSTFEDPGDVDWRTKGAVTPVKDQGACGSCWTFGTTGALE

GALFAQQKKLYNMSQQNLLDCSWDYGNNACNGGLDYQAYEWIIANGGLETTATYGAYR
NAPDYCHFNVDNAIGRMKGFVNVTSVQALNDALATIGPLSVSIDANLPSFYFYGGGYYD
DVECKSDLDSLDHSVLAVGVTTHNDQKYTLVKNSWSTHWGEDGYIKISQKNNLCGVATA
ATYPVLAD

>PITG_02423

MRASLIVALAAATASASPLSLPELTSSGGYGYVRSPDRSVSLTSPRPHDYIDVSKLPKNF
DWRNVNGTRYVSISRNQHIPHYCGSCWSFAATSALADRILIFKERNPGNKPSVEVHRGV
VLSPQVILNCDKKDNGCHGGDQLEAYRYIKEHGVPEEGCQRYAATGHDTGNTCTDMDV
CENCLPSKGCFPQKSYDKYYVSEVGTTLGEQQMMAEIYARGPIACSVAVTDGFLKYSG
GIFDDKTNATDVDHAISIVGWGEENGVPFWVLRNSWGSFWGESGWMRLVRGVNNVGV
EGECAFGVPRDDGWPTPTKIEEKEEDKVKEPQEETSVESTLGGCRQKLHFAGGERVIS
PLPHETMDVTDLPKSWDWRDVNGKNYVTWDKNQHIPKYCGSCWAQGTTSALSDRISIL
RNASWPEIALSPQVLINCHAGGTCNGGNPGLVYEYAHRHGIPDQTCQAYQAKNLQCDQ
FAICETCWPSKESFTPGVCEPIKKFAKYYVSEYGSVSGAERMKAEIYKRGPIGCGVHATS
KFESYTGGIYSEHVMFPLINHEISVAGWGYDEETDTEYWIGRNSWGTYWGENGWFRIQ
MHHNNLGIEQDCDWGVPLPDGSKPNDFVVDYQGNEAETMVVLHSSDDQCHLFTDVFG
RLLQLLLGCIAIFLLYIKRKLEFPIRPIKVWAMDVSKQSLGAFYIHCISVILSIVMIAASTENY
DEVRK

>PITG_02474

MLPVTVLSMGALARFTPSGSSQVMYGQAIQVVNTIGGTAAGSFTSSSKVGASGSASSF
NDVSVGSLESNETITFPPSSSSVGSAGSSTTVTTITESEEQQRFDQALADIAELQKLHPH
ATFSINSPFALLTSDEFLSYVNRFGIDPDSNPVKNSTSSTTGMFTMDAEDSGTTTPSSIGS
DIMTSAAAAGETVDWQEAGCVTAVKDQGECGACWAFSATAAMESGYCVATGSLPSLSD
QQLISCHTEDGNRGCGGGYAAYSLDWIANERRGKMCTLDTYPFTSENGNVAGCSMNS
CTEFNVGVTGYESVREDPGAIEDAVRKQPVSIFLYSGSTAFQYYSGGVLTGANCDKTGS
HSGLAVGFGETEDNILYWRIKNQWGTSWGEDGYVRVQRRFSGDSEGACGVELYATWP
TFDVSATPTSSPTPTTSAPSGTAATPSVTTATPPITTMAPSTNPVTDTPSATNAVVDQAAS
ASAASGSDSTITKVGVDQMTSATSGSTAHYETVKTDPVGGSIASTATPAATAIV

>PITG_03020 Pain2

MRIASTSLLLASLALADALKTPLEYEHEFSAWMKTHSVSFSDALEFAKRLENYIANDMYIM
EHNLENAWTGVKLDHNEFSSMSFEEFKFKMTGYVMPEGYLEQRLASRVDNLWSDVQV

PDSVDWQDKGGVTPVKNQGMCGSCWAFSTTGAVEGAAFVSSGKLVSLSEQELVDCDH
NGDMGCNGGLMDHAFAWIEDNGGICSEDDYEYKAKAQVCRDCEKVVKISGFQDVNPQ
DEHALKVAVAQQPVSVAIEADQKAFQFYKSGVFNLTCGTRLDHGVLAVGYGSENGQKF
WKVKNSWGSSWGEKGYIRLAREENGPAGQCGIASVPSYPFATLIKKDEETETQKIVEEP
RSVPAANAVESFPAEEARDFRPVNLADLFSSAKIKQCGDVGSAIIDFSDLEVTPSSPQRG
QPVSFFGNGNAKKDFDSANFKLGVKLAGTQVFGHSGKLCGDTHIPLPLGLGHIDVHGFA
CPMKKGKSSDLKVDVNLPIIAPAGNYEIQLTSDDDSNSSLFCVNVELDLTGGETAKKTHV
YEPISYM

>PITG_03414

MAPTWTTLLVLMAATVAPLITQAQDPSSFGTLQSCDDARCLWADRDGVAVSSDTMVTQF
LQDEGMDAGPSEFRRRMEDHVDYLEQVQKHAAGRDWAFSYAMGVNSRHLYHDGSRS
LSPADFVEQEHQASQRQQRRRLTEQRRLAIRETLDWCSKDNSHNQSICTDIKSQNQCG
SCWAFAAADAIETAVVVNAGTSPRSLSPQQFLECSSREMTATFDYCWADGGVDGSPWL
LTKMIWGSRNNACSGGMTHAAFADAAQLHWSLLSQLDLPYNEEDTSQASAATLANACD
NSSSDNAAASISGWEQVAGPSCDLSSDSTELLKLVLQQQPISVAINSGGSFDAYKGGIYT
CPNDGDFASSGDINHAVVLVGYGSDGSTDYWILKNSYGASWGEKGFLRLAMDSKINCG
LSVFPVIPTGAIAGAAHTAVDGGGEVEFVGMSPDSWIVCGIAVAVVTLFLTVIGVIYASRQR
NAFKETL

>PITG_03415

MQIRAITFVISTALVNGAAVQTTDRSVGTLVDCPSVRSQDSPCLWAGENGQVVDSRSLR
ELFIERNYVAYSDKESYGRNLQEHMTYIEDVSMYARQVGHDFSYHMGVNDRHLTSSST
RKLTPEQFVDQELTSANSRRLQEANSTNNSTISNSGSSEYWNWCDKDNSVGHSVCSPV
KSQKSCGSCWSFVAADAIETAVVITENASAAVSLSPQQFLTCSSLQTTQTFEYCWASDS
GVAGASWMQTEIKWESQNDGCNGGMTHGAFMDAAQNGWGLVTELTMPYDDSNSGS
SSATNLSSSCSVSADQAAASITGWEQIVGGDCTASKNCTLLLRSALEKQPIAVAITSNGG
FGEYAGGFYNCPNNGEMASKNDLNHALLLVGYGTDSSVGDYWILKNSYGSSWGDSGF
MKLVADAKINCGLNVFPVIPTGAKAGAQASTTVDSGGDKIFVGLSPTAWIGVAAATTIFTL
VTTAIGIGVSQRKLKVIRKQNSAMYAARTPTNTQNGF

>PITG_03416

MRVQATTEHSLGTLVDCPAFRSADSQCLWAGENGEVVDSKTLRDLLIRRNLVSFSERES
LTRNLQAHMTYIEDVHMYANSVGHDFSYHMGVNERHLTSSARRRLSPQQLVDQEVKSA

YSRRLKVEASGSTASATSTVSSSGSSEYWNWCDSDNSFGYSVCSSVKSQQNCGSCW
AFAAADAIETAVVIAENASAAVSLSPQQFLTCSTLETTQTFEYCWASDSGVDGASWMET
EIKWESQNNGCNGGMTHGAFIDAAQNGWSLVTELTMPYDDSSSSSTSSSNVSSACSVS
DNKTAASITGWEQVVGTDCTASNNCGTLLRTALEKQPIAVAINSEDPFGEYAGGFYSCPN
DGDLSSKDDVNHALVLVGYGTDASVGDYWILKNSYGSSWGASGFMKLVADSKVNCGLN
IFPVIPTGASAGAAASTSVDSGGDKVFVGLSPSTWIVVAAVTTIFTIVMTAIGMLFSQRRLK
AMRKQNSAMYATRPAANAEMAAH

>PITG_06926

MHSGIDYQRYLKEIDTAQADLDEWRSKFGDVAQKNGWMPVSEARSTDDQEEDLRQRIF
LTKQSIAKVQAANPNANFSIMSPFSAMTDEEFNKYVVNSYVSGNSTQNDNRTSARHLRS
AGSSDLTFDALSDSVDWSTSKCMAPIQSQGSCGSCWAFATVSAVESAQCIASGKELFVK
YSEQQLVSCNTKNWGCYGGSPFYAFDYVQQNGLCSEESYPYTFGGGYTRSCSRSCAA
QDTGLTGYAKVSGEDDLLRALDEHPVIVAVASANSVWKQYTGGVVSSCDSWQPDHAVV
AVGYDSSSIKIRNSWGPEWGEGGYIRLARSSSKAGTCGVLIDMSTPQM

>PITG_06927

MHAGINYERYLTEIDNIKAETDEWKAMFEKTCKENNWMPEYSTEERSSVDQDEDLRQRI
FMSKQDVLEAQASNPNAHFSIMTPFSALTKEEFASKVLNSYRSLRQEGTYTFTSMQDMI
NSLMQSLQQQMGGSWSTYAPVTPAPVVEPLVKKSSTPVRTEKAILKTANSVDWSASKC
MSPVQSQGQCGSCWAFASVAAVESLQCIKNGQSGINKYSEQQLVGCDSKNMGCGGGA
PVYAYEYIQKNGLCSESALPYTSSNGGAVSCSASCSKSQTGITGYERINEGDEAGLVEAL
KSQPVVVAVASGNAAWKQYTGGIMSTCETTQVDHAVLVVGYDDNTFKVRNSWGENWG
EAGYVRMARSSSGMGTCGMLTDMSRPKM

>PITG_06928

MHAGLDYHRYLAERAETRQELADWKANFGEMAQNNGWMPPSSGNSEERSTDDEDED
HLQRFYMTKQNISAIQALNPNANFSVNTPFTLLTNDEFAAYVGKSYRAYNASSVSTTRRL
RSWHRSSNSETTYSSSSPAKTTTTSTSSRTNSNPMLSGAANGGTTYDATNVKTVTSTG
ADGKSTTTTTTTTSSGPGRESTTVSTSSGSTPSNFGFGSDFSSLWQQWGNGFNFGGM
GRNDFQPETVKPAGSNSISTITDAPSTPLPTTAAPTPTPTETTVTPSPPKTSTSPATVTAT
DTSSTSSRVDWADSSCMSPIQSQGSCGDCWAFSTAAAIESGQCIHGGQKTLQKYSEQY
VQQNGLCTENDYPFTSSDGTAASCSTGCSAVDTGIKGYKTVDDASGLASAVAQQPVIIAV
ASGNNAWKQYTGGVISSCDTSELDHAVVVVGYTDSEWKIRNSWGDSWGEEGYIRLER

TSDTTGTCGMYGDMSYPTF

>PITG_08784

MNTALLLALAALVATADAAAPAVPTDRMEQFLEEKAVLHEELNEWKQSDAGLYAMEHGF
VPTASARNVSATTDEELRRFFLSKLLVEDAQAANPEAVFSTDTPFTLLTHDEFAKFIGESY
QRDSGPLKATSIADALPLNLTAASTEKDWTTSGCVAPVKNQGDCGSCWAFASVAALESA
ICLSGQPLTLLSEQQVVDCDKVSYACDRGWPSTALDYAKQSGGICTQEAYPYVSGDLGY
HQTCKSTCTRQKVTIRKVVAVPRSDAGLVQAIETQPVAVGVAADNPTWKQYKSGVVSSC
STSQLDHAVLAVGYSPSFFKIKSSWCPQWGENGFMRLKRGSGTSSSGTCGIIGSLSVYP

>PITG_12041 Pain1

MKVFGFLFLAATAAFAPAKALTTDLPSSLTASEQKTWEAFVDYALDYEKSYRNDANDHDV
VQLRFRSFATNLERIQTHNEAYERGEHSFTLGLNDLADLADAEYKQLLSYRTRDSKSSSA
SETFVKPENVEDLPATWDWREHSTVTPVKNQGQCGSCWAFSAVAAMECAYALSTGTLE
SLSEQELVDCTLNGIDTCNHGGEMSEGYEEIITNHKGKIDREEVYRYTAESKGVCNAKD
DKAIGHFTSYANVTSGDEAALQAAIATKGVQAVAIDASSFTFQLYRHGVYSWPLCGNAPD
ALDHGVAAAGYGVYKKKDYWLVKNSWGNSWGMKGYIMMSRNKDNQCGIATDATYPIM
TKEEVVEDRPIVLETTELASIM

>PITG_12916

MQTVVGYELRLHHAQTRTRRRALLGPTRSSDETRAEALANTERVVDEANAEHAAGQKS
FFMGYNELSDLTDEQYRAFLTSRPDRDSPRRKQRKKEPNRRKKGKQVTISFEIDDGSSE
SDDEEEDIEVPIELDWTTKDGGKYMTPIKNQGTCGSCWAFGVAAVESRYAIENNVQAS
PLSVEQVLSCSADLDHIRSKFEDNMTSSSEGCAGGMPFLTYMYLQLAQPHGISCESAYP
YVMATNETHPQCSSSLTSQVAVAWKSNGSAYKLVAASEKALLKAVTSGPVTANIDATGSG
FRHYAGGIYDAKDCLSDGDEVNHAVVVVGFGETEAGEKFWVIRNTWGTMWGEDGYMR
IARGSHVGSYGPCNLYVYADYPVNLTVGSNATSGEPSCPVAASKFESLPLMKLVGLSGN
QIVMLVLCTVLTVLAGVTLYHGTEFIQNRKEAAGELRYQDSYARWILPSRDQIAAALAQR
RQRPATEQ

>PITG_13074

MKFPLVLEGSCHGFTTKETCMKNHCAWCVCAAVPSSCYSPEEADQLPPAIFQCDKGQH
LQSWDLTTVPSTSLELNSLFEAWKGLHGKSYDSPIQNELRRGIFEVNARSVAAHNSKNH

KSFVMELNEFADTTWDEFQSWYLGAPQECSATETTDVVYGEVPVQKDWRADGAVSPV
KNQGKCGSCWTFSTTGCLESHVKLKHGEFTILSEQNLLDCAQNFDNHGCNGGLPSHAF
EYIKYNGGLDTEETYPYEAKEGKCKFNTYHVGVQVDQVVNITTRNENELRAAVGSTGPV
SIAFQVVSDFRFYESGVYESKECRSDEKDVNHAVLAVGYGVEDGKDHWIVKNSWGSQ
WGMDGFFQIARGSNMCGVAVCASYPVVV

>PITG_16276

MLLSICSGSRTSALIKVFGFLFLAATAAFAPAKALTTDLPSSPTASEQKTWEAFVDYALDY
EKSYRNDANDHDVVQLRFRAFATNLERIQTHNEAYDSKSSSASETFVKPENVEDLPATW
DWHEHSTATPVKNQGQCGSCWAFSAVAAMECAYALSTGTLESLSEQELVDCTLNGIDT
CNHGGEMSEGYEEIIINHKGKIDREEDYGYTAESKGVCNAKDDKAIGHFTSYANVTSDDE
AALQAAIATKGVQAVAIDDSSFTFQLYRHGVYSWPLCGNAPDALDHGVAAAGYGVYKKK
DYWLVKNSWGNSWGMKGYIMMSRNKDNQCGIATDATYPIMTKEEVVEDRPIVLETTKL
ASIM

>PITG_17314

MNTALLLLLASVAATDAAVPLVPTDRMAQFLQEKSGLESELNTWKQSDAGQYAKDHGFL
PIPTSRDVGGQADEELRRFFLTKLMIQEAQVTNPEAIFSTDTPFTLMTDQEFVNTDKDWT
TSECVVPVKNQGQCGSCWAFAAVAALESAICLSGQPLTPLSEQQVVDCDEASYACQGG
FPGDALTFIQQSGGVCTEEAYPYVSGDSGDRTCKSSCTREAVTIRKVVGVPESDAGLV
QAINTQPVAVGVAAGNPTWKQYKSGIVSSCTTSELDHAVLAVGYSPSYFKIKNSWSTQW
GEEGYMRLKRGAGTSSAGTCGIIGPKSVYPQL

>PITG_20589

MLGGSHIDEYFLDYIYVREGPVDEDVFSFLPSSMNCTEMPGDDGGPSRIPKQDIHMLMP
EGSTVKKEIFDNFSATHEKEYNDDAEVQRMATFHHNLRFINGENRKGLPYHLEVNKFA
DLSYEERRALHRPSRVKRAKDNKAMAVHELSTFEDPGDVDWRTKGAVTPVKDQGACG
SCWTFGTTGALEGALFAQQKKLYNMSQQNLLDCSWDYGNNACNGGLDYQAYEWIIAN
GGLETTATYGAYRNAPDYCHFNVDNAIGRMKGFVNVTSVQALNDALATIGPLSVSIDANL
PSFYFYGGGYYDDVECKSDLDSLDHSVLAVGVTTHNDQKYTLVKNSWSTHWGEDGYIK
ISQKNNLCGVATAATYPVLAD

>PITG_22022

MLPVTVLSMGALARFTPSGSSQVMYGQAVQVVNTIGGTAAGSFTSSSKVGASGSASSF
NDVSVGSLESNETITFPPSSSSVGSAGSSTTVTTITESEEQQRFDQALADIAELQKLHPH
ATFSINSPFALLTSDEFLSYVNRFGIDPDSNPVKNSTSSTAGMFTMDAEDSGTTTPSSIGS
DIMTSAAAAGETVDWQEAGCVTAVKDQGECGACWAFSATAAMESGYCVATGSLPSLSD
QQLISCHTEDGNRGCGGGYAAYSLDWIANERRGKMCTLDTYPLTSGYESVREDPGAIE
DAYYSGGVLTGANCDKTGSHSGLAVGFGETEDNILYWRIKNQWGTSWGEDGYVRVQR
RFSGDSEGACGVELYATWPTFDVSATPTSSPTPTTSAPSGTAATPSVTTATPPITTMAPS
TNPVTDTPSATNAVLPAQVPLRDLTRLSRRLALTK
