# Supplementary material for: Pathogen-inspired engineering of plant protease enhances late blight resistance
Source: Proc Natl Acad Sci U S A. 2026 Jan 9;123(2):e2524700123. doi: 10.1073/pnas.2524700123 (PMC12799129; doi:10.1073/pnas.2524700123)
Supplement: Supplementary file 3 — Dataset S02 (PDF) [file pnas.2524700123.sd02.pdf]

Supplemental File S2: Protein sequences of all PLCPs used for Figure 1C and 1D.

>Pain1

MKVFGFLFLAATAAFAPAKALTTDLPSSLTASEQKTWEAFVDYALDYEKSYRNDAND
HDVVQLRFRSFATNLERIQTHNEAYERGEHSFTLGLNDLADLADAEYKQLLSYRTRD
SKSSSASETFVKPENVEDLPATWDWREHSTVTPVKNQGQCGSCWAFSAVAAMEC
AYALSTGTLESLSEQELVDCTLNGIDTCNHGGEMSEGYEEIITNHKGKIDREEVYRYT
AESKGVCNAKDDKAIGHFTSYANVTSGDEAALQAAIATKGVQAVAIDASSFTFQLYR
HGVYSWPLCGNAPDALDHGVAAAGYGVYKKKDYWLVKNSWGNSWGMKGYIMMS
RNKDNQCGIATDATYPIMTKEEVVEDRPIVLETTELASIM

>*P. cactorum* Pain1

MKMFGSLFLAATAVFAPAKALTTDLPSSLSASELQTWEAFVDYALDYEKSYRYDAND
QDLVQLRFRAFATNLDRIQAHNGAYERGEHSFTLGLNELADLTDVEYKQRLSYRGR
DSKTSGAPETFVKPENVEDLPATWDWREHNTVTPVKNQGQCGSCWAFSAVAAME
SAYALSTGTLESFSEQELVDCTLNGIDTCNHGGEMSEGYEEIINNHKGKIDREKDYE
YTAESKGVCNAKDDKAIGHFTSYANVTSGDEGALQAAIATKGVQAVAIDASSFTFQLY
RHGVYSWPLCGNAPDALDHGVAAAGYGVYKKKDYWLVKNSWGDSWGMKGYIMM
SRNKDNQCGIATDATYPIMTKEEEIVEGRPIVLETTEVASIM

>*P. nicotianae* Pain1

MKVFTSLLLAATAAFAPASALTTDLPSSLSASEQQTWEAFVDYALDYEKSYRYDTND
QDLVQQRFRAFATNLERIQMHNAAYERGEHSFTLGLNELADLTDAEYKQLLSYRAS
DSKASRASETFVKPDNIEDLPATWDWREHNTVTPVKNQGQCGSCWAFSAVAAMEC
AYALSTGTLESFSEQELVDCTLNGIDTCNHGGEMSEGYEEIINNHKGKIDREEDYEYT
AESKGVCNAKDDKAIGHFTSYANVTSGDEAALQAAIATKGVQAVAIDASSFTFQLYR
HGVYSWPLCGNAPDALDHGVAAAGYGVYKKKDYWLVKNSWGDSWGMKGYIMMS
RNKNNQCGIATDATYPIMAKEEEIVVDRPIVLETTEVASIM

>*P. capsici* Pain1

MKVFGSLFAAAAALASTNALTTGLPSSLSSSQQQTWEAFVDYALDYEKDYRYYSND
QDLVQLRFQAFATNLERIQTHNEAFERGEHSFTLGLNDLADLTDSEYKQLLSYRANQ
AKTSGASETFVKPENVEDLPATWDWREHDAVTPVKNQGQCGSCWAFSAVAAMES

AYALTVGTLESFSEQELVDCTLNGIDTCNHGGEMSEGYEEIINNHKGKIDREEDYEYT
AESKGVCNAKDDKAIGHFTSYANITSGDEGALQAAIATKGVQAVAIDASSFTFQLYRH
GVYSWPLCGNAPDALDHGVAAAGYGVYKTKDYWLVKNSWGNSWGMKGYIMMSR
NKDNQCGIATDATYPIMTKEEEVFTETKDRAIVLESTEVASIM

>*P. sojae* Pain1

MKVLSSLFLAAVALAPCHALTSDPPASVSASERQTWEAFVDYALDYEKSYRSDAND
QALVQHRFRAFATNLQRIEAHNAAFERGEFSFTLGLNDLADLSDAEYKQLLSYRARD
SKGASETFSVSPEDVKDLPDSWDWRQHGAVTPVKNQGQCGSCWAFSAVAAMESA
YQLSTGKLESFSEQELVDCTLGGVDDCSHGGEMSEGYEEIIKHHGGKIDREEDYEY
TAESRGVCNAKDDKAIGHFTAYANVTSGDEAALQAAITTKGVQAVAIDASSFTFQLYR
HGVYSWPLCGNAPDALDHGVAAAGYGVYKKKDFWLVKNSWGDSWGMKGYIMMS
RNKDNQCGIATDASYPIMTKEDVAMEPRPVVVEETTEVASIM

>*P. ramorum* Pain1

MKVFGPLFLAAATAFAHANALTSDLPPSLTASEQQTWGAFVDYALDFQKDYRYFGN
EQDLVQHKFRAFATNLERINAHNEGYERGEFSFTLGLNELADLTDSEYKQLLSYRAS
ESEATETFSKPENVEDLPDIWDWREHDTVTPVKNQGQCGSCWAFSAVAAMECAYA
LSTGTLESFSEQELVDCTLDGIDSCNHGGEMSEGYEEIIKRHGGKIDREKDYEYTAE
SKGVCNAKDEKAIGHFTAYANVTSGDEGALQAAIATKGVQAVAIDASSFTFQLYRHG
VYSWPLCGNAPDALDHGVAAAGYGVYKKKDFWLVKNSWGDSWGMKGYIMMSRN
KDNQCGIATDASYPIMTKGEETALKMESVAMVEETTEVASIIYGKDVYDYGYGYPDY
SSYGGGFGYYSDSYSTYGYGYGYPGNFGYGYGYPGYFGYESSATDAGAVTKESA
TVAVTVASVKPMETKES

>*P. lilii* Pain1

MGISDSRLESYLLSLAAAAAVKCDLDAKGCVFEYMCLLKYKVVAMGKDRLIENPFPE
LQVSAHDQQRLKDLANTLIMTNLDKCNAFVSSDKGKVDPRRWKPITEREQLRVYAE
RRDSAQAAAGSNLTGSGLPMILCVGSVEGKLNDLMYGVMSEDLETMRIKASWRLH
QRSLLPQHFIENPCSASTHTNIKSSSSGGSPILSNPRSSAKNQVPTMKVSGPLLLAA
TVSSSLALTTEPPASLGAEQQQTWEAFVDYALDFQKDYRDAANDAALVQRRFRAFA
TNVERIRAHNEAAERGEFSFTLGLNDLADLADAEYRQLLSYRASSAKAAAVETFTRP
ESLDDLPDSWDWRKHDAVTPVKNQGQCGSCWAFSAVAAMECAYALSTGTLESFSE
QELVDCTLGGVDDCNHGGEMSEGYEEIIQHHGGKIDREADYEYTAESKGVCNAKD

DKAVGHFTAYANVTSGDELALQAAIATKGVQAVAIDASSFTFQLYRHGVYSWPLCGN
APDALDHGVAAAGYGVYKKKDFWLVKNSWGDSWGMKGYIMMSRNKDNQCGIATD
ASYPIMTKEELSVKTEARAVVQETTEVASVM

>*P. cinnamomi* Pain1

MKVLSSLLLSSVAFAPCSALTSEPPAAVGESQRQAWEAFVDYALDFSKSYQYQDNG
QALVLRRFRAFATSLERIEAHNAAYERGEFSFTLGLNELADLSDAEYRQLLSYRADS
KRSGGAAETFSVAPESVQSLPESWDWRQHGAVTPVKNQGQCGSCWAFSAVAAME
SAYQLSTGTLESFSEQELVDCTLGGVDDCNHGGEMSEGFEEIIQHHGGKIDREKDY
EYTAESRGVCNAKDDKALGHFSAYANVTSGDEAALQAAVATKGVQAVAIDASSFTFQ
LYRHGVYSWPLCGNAPDALDHGVAAAGYGVYKKKDFWLVKNSWGDSWGMKGYI
MMSRNKDNQCGIATDASYPIMTKEEDVPSEPRPVVVEETTEVASIMFEGHKFCIIMP
QTV

>Pain2

MRIASTSLLLASLALADALKTPLEYEHEFSAWMKTHSVSFSDALEFAKRLENYIANDM
YIMEHNLENAWTGVKLDHNEFSSMSFEEFKFKMTGYVMPEGYLEQRLASRVDNLW
SDVQVPDSVDWQDKGGVTPVKNQGMCGSCWAFSTTGAVEGAAFVSSGKLVSLSE
QELVDCDHNGDMGCNGGLMDHAFAWIEDNGGICSEDDYEYKAKAQVCRDCEKVV
KISGFQDVNPQDEHALKVAVAQQPVSVAIEADQKAFQFYKSGVFNLTCGTRLDHGVL
AVGYGSENGQKFWKVKNSWGSSWGEKGYIRLAREENGPAGQCGIASVPSYPFATL
IKKDEETETQKIVEEPRSVPAANAVESFPAEEARDFRPVNLADLFSSAKIKQCGDVG
SAIIDFSDLEVTPSSPQRGQPVSFFGNGNAKKDFDSANFKLGVKLAGTQVFGHSGK
LCGDTHIPLPLGLGHIDVHGFACPMKKGKSSDLKVDVNLPIIAPAGNYEIQLTSDDDS
NSSLFCVNVELDLTGGETAKKTHVYEPISYM

>*P. cactorum* Pain2

MRIASTSFLLASLALTDALKTPLEYEHEFSAWMKTHSISISDALEFAKRLENYIANDMY
ILEHNLENAWTGVKLGHNEFSHMSFDEFKFKMTGYVMPEGYLEQRLASRVDGLWS
DVQVAESVDWQDKGGVTPVKNQGMCGSCWAFSTTGAVEGATFVSSGKLVSLSEQ
ELVDCDHNGDMGCNGGLMDHAFAWIEDHGGICSEDDYEYKAKAQVCRDCEKVVK
VTGFQDVNPQDEHALKVAVAQQPVSVAIEADQKAFQFYKSGVFNLTCGTRLDHGVL
AVGYGSDNGQKFWKVKNSWGSSWGENGYIRLAREENGPAGQCGIASVPSYPFATL
ISKDEQTEKIEKVVEEPRSVPTDESVESFPAEEARDFRPMNLADLFSSAKITQCGDV

SSAIIDFSDLEVTPSSPQRGQPVSFFGNGNSKRDFASANFKLGVKLAGTQVFGHSG
KLCGDTHVPLPLGLGHIDVHGFACPMKKGKFSDLKVDVNLPIIAPAGNYEIQLTSDDD
SNSPLFCVNVELDLTGGEGSAKKTHVYEPISYM

>*P. nicotianae* Pain2

MRIASSSLLLASLALADALKTPLEYEHEFSAWMKTHSISFSDALEFAKRLENYIANDM
YILEHNLENAWTGVKLGHNEFSHMSFDEFKFKMTGFEMPDGYLEQRLASRVDGLW
TDVQVPESVDWQDKGGVTPVKNQGMCGSCWAFSTTGAVEGAAYVSSGKLVSLSE
QELVDCDHNGDMGCNGGLMDHAFAWIEDHGGICSEDDYEYKAKAQVCRDCEKVV
KVTGFQDVNPQDEHALKVAVSQQPVSVAIEADQKAFQFYKSGVFNLTCGTRLDHGV
LAVGYGSDNGQKFWKVKNSWGSSWGENGYIRLTREENGPAGQCGIASVPSYPFAT
LISKDEQTETEKVVEEPRSVPADNPVESFPAEERDFRPMNLADLFSSAKITQCGDVG
SAIIDFSDLEVTPSSPQRGQPVSFFGNGNAKRDFASANFKLGVKLAGTQVFGHSGK
LCGDTHVPLPLGLGHIDVHGFACPMKKGKFSDLKVDVNLPIIAPAGNYEIQLTSDDDS
NSPLFCVNVELDLTSGEGAAKKTHVYEPISYM

>*P. capsici* Pain2

MRIATASLLLASLALADALKTPLEYEHEFSAWMKAHAISFSDALEFAKRLENYIANDM
YIIEHNIENAWTGVKLGHNEFSHMSFDEFKFKMTGLALPEGYVEQRLASRVDGLWS
DVQVAEAVDWQDKGGVTPVKNQGMCGSCWAFSTTGAVEGAAFVSSGKLVSLSEQ
ELVDCDHNGDMGCNGGLMDHAFAWIEDNGGICSEDDYEYKAKAQVCRKCEKAVK
VTGFQDVNPQDEHALKVAVEQQPVSVAIEADQKAFQFYKSGVFNLTCGTRLDHGVL
AVGYGEDNGQKFWKVKNSWGSSWGEQGYIRLAREENGPAGQCGIASVPSYPFAT
LISKDEKTEVVEPRSVPADKPVDSFPAEEARDFRPTNLADLFSSAKISQCGDVSSAVI
NFSDLEVTPTSPQRGQPVSFFGNGDAKQDFASANFKLGVKLAGTQVFGHSGKLCG
DTHVPLPLGLGHIDVHGFACPMKKGKFSDLKVDVNLPIIAPAGNYEILLTSDDDSNSP
LFCVNVELDLTGDNEAAKKTHVYEPISYM

>*P. ramorum* Pain2

MRVLSGALLFASLALCDALKSPLEYEHEFSAWMQTHGVSFSDALEFARRLENYIAND
MYILEHNTEDAWTGVTLGHNEFSHLSFDEFKFKMTGFVMPDGYLEQRLASRVDGL
WGDVEVPDAVDWQDKGGVTPIKNQGMCGSCWAFSTTGAVEGATFVSSGKLPSLS
EQELVDCDHNGDMGCNGGLMDHAFAWIEDHGGLCGEDDYEYKGKAEVCRKCDN
VVKVTGFQDVNPQDEHALKVAVAQQPVSVAIEADQKAFQFYKAGVFNLTCGTRLDH

GVLAVGYGADNGQKFWKVKNSWGPSWGEHGYIRLAREENGPAGQCGIASVPSYP
FATLISKDELTEETEEVEEPRSVPADKPVDSFPADEARDFRPKNLADLFSSAKISQCG
DVGSAIIDFDHLEVTPTSPQRGQPVVFFGNGNSKQDFDSANFKLGVKLAGTQVFGH
SGKLCGDTHVPLPLGLGHIDVHGFACPMKKGKFSDLKVDVNLPIIAPAGNYEILLTSD
DDSNSPLFCVNVELDLTGADEATTKTHVYEPLSYM

>*P. sojae* Pain2

MRIVGGLLLASLALADALKSPLEYEHEFSAWMSAHGVTFSDALEFARRLENYIANDM
YILEHNAENAWTGVKLGHNAFSHMSFDEFKFKMTGLVLPEGYLEQRLASRVDGLWS
DVEVPSAVDWVDKGGVTPVKNQGMCGSCWAFSTTGAVEGATFVSSGKLLSLSEQ
ELVDCDHNGDMGCNGGLMDHAFQWIEDHGGICSEDDYEYKAKAQVCRKCDSVVK
VTGFQDVNPQDEHALKVAVAQQPVSVAIEADQKAFQFYKSGVFNLTCGTRLDHGVL
AVGYGNDNGQKFWKVKNSWGASWGEQGYIRLAREENGPAGQCGIASVPSYPFAT
LINKDEQETEKVVEEPRSVPADKPVDSFPAEPERDFRPKNLADLYSSAKITQCGDVS
SAIIDFDDLEVTPTSPQRGQPVSFFGNGNAKQDFSSANFKLGVKLAGTQVFGHSGK
LCGDTHVPLPLGLGHIDVHGFACPMKKGKFSDLKVDVNLPIIAPAGNYEIMLTSDDNS
NSQLFCVNVELDLTDSDATKKAHVYEPLSYM

>*P. lilii* Pain2

MRVVGSLLLASLALSDALKTPLEYEREFSSWMQTHGVTFSDALEFARRLENYIAND
MYILEHNAENAWTGVTLGHNEFSHMSFDEFKFKMTGLVLPDGYLEQRLAARVDGL
WTDVQVPDEVDWVEKGAVTHVKNQGMCGSCWAFSTTGAVEGATYVSSGKLLSLS
EQELVDCDHNGDMGCNGGLMDHAFAWIEDHGGLCSEDDYEYKGKAQVCRKCENV
VKVTGFQDVNPQDEHALKVAVAQQPVSVAIEADQKAFQFYKSGVFNLTCGTRLDHG
VLAVGYGVDNGQKFWKVKNSWGSSWGESGYIRLAREENGPAGQCGIASVPSYPFA
TLITKDEQVEEKTKVDEVVEEPRSVPADHPVDSFPAEAARDFRPKNLADLFSSAKIT
QCGDVSSAIIDFDHLEVTPASPQRGQPVVFFGNGNSKQDFASANFKLEVKLAGTQV
FGHTGELCGDTHVPLPLGLGHIDVHGFACPMKKGKFSDLKVDVNLPIIAPAGNYEILL
TSDDDSNSPLFCVNVELDLTSGADDAAKKTHVYEPLSYM

>*P. cinnamomi* Pain2

MRVAATVALLLASLAPAEGALKSPLEYEHEFSAWMAAHGVSFSNALEFARRLENYIA
NDMYIAEHNAENAWTGVTLGHNAFSHMSFDEFKFQMTGLALPAGYVEQRLAARVS
GLWSDVEAPKEVDWVAKGAVTPVKNQGMCGSCWAFSTTGAVEGAAFVSSGKLPS

LSEQELVDCDHNGDMGCNGGLMDHAFAWIEDHGGICSEEDYEYKAKAQVCRKCD
SVVKVTGFQDVNAQDEHALKLAVAQQPVSVAIEADQKAFQFYKSGVFNLTCGTRLD
HGVLAVGYGSVDGQKFWKVKNSWGASWGEHGYIRLAREENGPAGQCGIASVPSY
PFATLINKDEKTAEVVEEPRFVPADKPVDSFPAEPARDFRPKNLADLYSSAKITQCGD
VSSAIIDFDHLEVAPTSPQRGQPVSFFGNGNSKQDFDSANFKLGVKLAGTQVFGHT
GKLCGDTHVPLPLGLGHIDVHGFACPVKKGKFSDLKVDVNLPIIAPAGNYEILLTSDN
DSNSPLFCVNVELDLTDGDAAKKTHVYEPLSYM

>PITG_03414

MAPTWTTLLVLMAATVAPLITQAQDPSSFGTLQSCDDARCLWADRDGVAVSSDTMV
TQFLQDEGMDAGPSEFRRRMEDHVDYLEQVQKHAAGRDWAFSYAMGVNSRHLY
HDGSRSLSPADFVEQEHQASQRQQRRRLTEQRRLAIRETLDWCSKDNSHNQSICT
DIKSQNQCGSCWAFAAADAIETAVVVNAGTSPRSLSPQQFLECSSREMTATFDYCW
ADGGVDGSPWLLTKMIWGSRNNACSGGMTHAAFADAAQLHWSLLSQLDLPYNEE
DTSQASAATLANACDNSSSDNAAASISGWEQVAGPSCDLSSDSTELLKLVLQQQPI
SVAINSGGSFDAYKGGIYTCPNDGDFASSGDINHAVVLVGYGSDGSTDYWILKNSYG
ASWGEKGFLRLAMDSKINCGLSVFPVIPTGAIAGAAHTAVDGGGEVEFVGMSPDSW
IVCGIAVAVVTLFLTVIGVIYASRQRNAFKETL

>Papain

IPEYVDWRQKGAVTPVKNQGSCGSCWAFSAVVTIEGIIKIRTGNLNEYSEQELLDCD
RRSYGCNGGYPWSALQLVAQYGIHYRNTYPYEGVQRYCRSREKGPYAAKTDGVR
QVQPYNEGALLYSIANQPVSVVLEAAGKDFQLYRGGIFVGPCGNKVDHAVAAVGYG
PNYILIKNSWGTGWGENGYIRIKRGTGNSYGVCGLYTSSFYPVKN
