# Supplementary material for: Pathogen-inspired engineering of plant protease enhances late blight resistance
Source: Proc Natl Acad Sci U S A. 2026 Jan 9;123(2):e2524700123. doi: 10.1073/pnas.2524700123 (PMC12799129; doi:10.1073/pnas.2524700123)
Supplement: Supplementary file 4 — Dataset S03 (PDF) [file pnas.2524700123.sd03.pdf]

Supplemental File S3: Protein sequences of PLCPs used for Figure 2A and Figure S2.

>AT1G47128.1 RD21A

MGFLKPTMAILFLAMVAVSSAVDMSIISYDEKHGVSTTGGRSEAEVMSIYEAWLVKHGKAQSQNSLVEK
DRRFEIFKDNLRFVDEHNEKNLSYRLGLTRFADLTNDEYRSKYLGAKMEKKGERRTSLRYEARVGDELP
ESIDWRKKGAVAEVKDQGGCGSCWAFSTIGAVEGINQIVTGDLITLSEQELVDCDTSYNEGCNGGLMDY
AFEFIIKNGGIDTDKDYPYKGVDGTCDQIRKNAKVVTIDSYEDVPTYSEESLKKAVAHQPISIAIEAGGRAF
QLYDSGIFDGSCGTQLDHGVVAVGYGTENGKDYWIVRNSWGKSWGESGYLRMARNIASSSGKCGIAIE
PSYPIKNGENPPNPGPSPPSPIKPPTQCDSYYTCPESNTCCCLFEYGKYCFAWGCCPLEAATCCDDNY
SCCPHEYPVCDLDQGTCLLSKNSPFSVKALKRKPATPFWSQGRKNIA

>AT5G43060.1 RD21B

MGFLKLSPMILLLAMIGVSYAMDMSIISYDENHHITTETSRSDSEVERIYEAWMVEHGKKKMNQNGLGAE
KDQRFEIFKDNLRFIDEHNTKNLSYKLGLTRFADLTNEEYRSMYLGAKPTKRVLKTSDRYQARVGDALPD
SVDWRKEGAVADVKDQGSCGSCWAFSTIGAVEGINKIVTGDLISLSEQELVDCDTSYNQGCNGGLMDY
AFEFIIKNGGIDTEADYPYKAADGRCDQNRKNAKVVTIDSYEDVPENSEASLKKALAHQPISVAIEAGGRA
FQLYSSGVFDGLCGTELDHGVVAVGYGTENGKDYWIVRNSWGNRWGESGYIKMARNIEAPTGKCGIA
MEASYPIKKGQNPPNPGPSPPSPIKPPTTCDKYFSCPESNTCCCLYKYGKYCFGWGCCPLEAATCCDD
NSSCCPHEYPVCDVNRGTCLMSKNSPFSVKALKRTPAIPFWAKSRKHIA

>AT3G19390.1 RD21C

MATSIKSITLALLIFSVLLISLSLGSVTATETTRNEAEARRMYERWLVENRKNYNGLGEKERRFEIFKDNLK
FVEEHSSIPNRTYEVGLTRFADLTNDEFRAIYLRSKMERTRVPVKGEKYLYKVGDSLPDAIDWRAKGAVN
PVKDQGSCGSCWAFSAIGAVEGINQIKTGELISLSEQELVDCDTSYNDGCGGGLMDYAFKFIIENGGIDT
EEDYPYIATDVNVCNSDKKNTRVVTIDGYEDVPQNDEKSLKKALANQPISVAIEAGGRAFQLYTSGVFTG
TCGTSLDHGVVAVGYGSEGGQDYWIVRNSWGSNWGESGYFKLERNIKESSGKCGVAMMASYPTKSS
GSNPPKPPAPSPVVCDKSNTCPAKSTCCCLYEYNGKCYSWGCCPYESATCCDDGSSCCPQSYPVCDL
KANTCRMKGNSPLSIKALTRGPAIATTKSTNMLVGSA

>AT4G36880.1 RDL1

MAPSTKVLSLLLLYVVVSLASGDESIINDHLQLPSDGKWRTDEEVRSIYLQWSAEHGKTNNNNNGIINDQ
DKRFNIFKDNLRFIDLHNENNKNATYKLGLTKFTDLTNDEYRKLYLGARTEPARRIAKAKNVNQKYSAAVN

GKEVPETVDWRQKGAVNPIKDQGTCGSCWAFSTTAAVEGINKIVTGELISLSEQELVDCDKSYNQGCNG
GLMDYAFQFIMKNGGLNTEKDYPYRGFGGKCNSFLKNSRVVSIDGYEDVPTKDETALKKAISYQPVSVAI
EAGGRIFQHYQSGIFTGSCGTNLDHAVVAVGYGSENGVDYWIVRNSWGPRWGEEGYIRMERNLAASK
SGKCGIAVEASYPVKYSPNPVRGNTISSV

>AT3G19400.1 RDL2

MAATPIRVIVSALVILSVLLLSSSLGVATETEIERNETEVRLMYEQWLVENRKNYNGLGEKERRFKIFKDNL
KFVDEHNSVPDRTFEVGLTRFADLTNEEFRAIYLRKKMERTKDSVKTERYLYKEGDVLPDEVDWRANGA
VVSVKDQGNCGSCWAFSAVGAVEGINQITTGELISLSEQELVDCDRGFVNAGCDGGIMNYAFEFIMKNG
GIETDQDYPYNANDLGLCNADKNNNTRVVTIDGYEDVPRDDEKSLKKAVAHQPVSVAIEASSQAFQLYK
SGVMTGTCGISLDHGVVVVGYGSTSGEDYWIIRNSWGLNWGDSGYVKLQRNIDDPFGKCGIAMMPSY
PTKSSFPSSFDLLSEI

>AT3G43960.1 RDL3

MAISFRTLALLTLSVLLISISLGVVTATESQRNEGEVLTMYEQWLVENGKNYNGLGEKERRFKIFKDNLKRI
EEHNSDPNRSYERGLNKFSDLTADEFQASYLGGKMEKKSLSDVAERYQYKEGDVLPDEVDWRERGAV
VPRVKRQGECGSCWAFAATGAVEGINQITTGELVSLSEQELIDCDRGNDNFGCAGGGAVWAFEFIKEN
GGIVSDEVYGYTGEDTAACKAIEMKTTRVVTINGHEVVPVNDEMSLKKAVAYQPISVMISAANMSDYKSG
VYKGACSNLWGDHNVLIVGYGTSSDEGDYWLIRNSWGPEWGEGGYLRLQRNFHEPTGKCAVAVAPVY
PIKSNSSSHLLSPSVFKLVVLFVFQLISLALL

>AT4G11310.1 RDL4

MGSAKSAMLILLVAMVIASCATAIDMSVVSYDDNNRLHSVFDAEASLIFESWMVKHGKVYGSVAEKERRL
TIFEDNLRFINNRNAENLSYRLGLTGFADLSLHEYKEVCHGADPRPPRNHVFMTSSDRYKTSADDVLPK
SVDWRNEGAVTEVKDQGHCRSCWAFSTVGAVEGLNKIVTGELVTLSEQDLINCNKENNGCGGGKLETA
YEFIMKNGGLGTDNDYPYKAVNGVCDGRLKENNKNVMIDGYENLPANDESALMKAVAHQPVTAVIDSSS
REFQLYESGVFDGSCGTNLNHGVVVVGYGTENGRDYWLVKNSRGITWGEAGYMKMARNIANPRGLC
GIAMRASYPLKNSFSTDKSSIA

>AT4G11320.1 RDL5

MGYAKSAMLIFLLALVIASCATAMDMSVVSSNDNHHVTAGPGRRQGIFDAEATLMFESWMVKHGKVYDS
VAEKERRLTIFEDNLRFITNRNAENLSYRLGLNRFADLSLHEYGEICHGADPRPPRNHVFMTSSNRYKTS
DGDVLPKSVDWRNEGAVTEVKDQGLCRSCWAFSTVGAVEGLNKIVTGELVTLSEQDLINCNKENNGCG

GGKVETAYEFIMNNGGLGTDNDYPYKALNGVCEGRLKEDNKNVMIDGYENLPANDEAALMKAVAHQPV
TAVVDSSSREFQLYESGVFDGTCGTNLNHGVVVVGYGTENGRDYWIVKNSRGDTWGEAGYMKMARNI
ANPRGLCGIAMRASYPLKNSFSTDKVSVA

>AT4G23520.1 RDL6

MGFVRPVCMTILFLLIVFVLSAPSSAMDLPATSGGHNRSNEEVEFIFQMWMSKHGKTYTNALGEKERRF
QNFKDNLRFIDQHNAKNLSYQLGLTRFADLTVQEYRDLFPGSPKPKQRNLKTSRRYVPLAGDQLPESVD
WRQEGAVSEIKDQGTCNSCWAFSTVAAVEGLNKIVTGELISLSEQELVDCNLVNNGCYGSGLMDTAFQF
LINNNGLDSEKDYPYQGTQGSCNRKQSTSNKVITIDSYEDVPANDEISLQKAVAHQPVSVGVDKKSQEF
MLYRSCIYNGPCGTNLDHALVIVGYGSENGQDYWIVRNSWGTTWGDAGYIKIARNFEDPKGLCGIAMLA
SYPIKNSASNA

>AT3G48350.1 CEP3

MKLFFIVLISFLSLLQASKGFDFDEKELETEENVWKLYERWRGHHSVSRASHEAIKRFNVFRHNVLHVHR
TNKKNKPYKLKINRFADITHHEFRSSYAGSNVKHHRMLRGPKRGSGGFMYENVTRVPSSVDWREKGAV
TEVKNQQDCGSCWAFSTVAAVEGINKIRTNKLVSLSEQELVDCDTEENQGCAGGLMEPAFEFIKNNGGI
KTEETYPYDSSDVQFCRANSIGGETVTIDGHEHVPENDEEELLKAVAHQPVSVAIDAGSSDFQLYSEGVF
IGECGTQLNHGVVIVGYGETKNGTKYWIVRNSWGPEWGEGGYVRIERGISENEGRCGIAMEASYPTKL
SSTPSTHESVVRDDVKDEL

>AT3G48340.1 CEP2

MKKLLLIFLFSLVILQTACGFDYDDKEIESEEGLSTLYDRWRSHHSVPRSLNEREKRFNVFRHNVMHVHN
TNKKNRSYKLKLNKFADLTINEFKNAYTGSNIKHHRMLQGPKRGSKQFMYDHENLSKLPSSVDWRKKG
AVTEIKNQGKCGSCWAFSTVAAVEGINKIKTNKLVSLSEQELVDCDTKQNEGCNGGLMEIAFEFIKKNGGI
TTEDSYPYEGIDGKCDASKDNGVLVTIDGHEDVPENDENALLKAVANQPVSVAIDAGSSDFQFYSEGVF
TGSCGTELNHGVAAVGYGSERGKKYWIVRNSWGAEWGEGGYIKIEREIDEPEGRCGIAMEASYPIKLSS
SNPTPKDGDVKDEL

>AT5G50260.1 CEP1

MKRFIVLALCMLMVLETTKGLDFHNKDVESENSLWELYERWRSHHTVARSLEEKAKRFNVFKHNVKHIH
ETNKKDKSYKLKLNKFGDMTSEEFRRTYAGSNIKHHRMFQGEKKATKSFMYANVNTLPTSVDWRKNGA
VTPVKNQGQCGSCWAFSTVVAVEGINQIRTKKLTSLSEQELVDCDTNQNQGCNGGLMDLAFEFIKEKG
GLTSELVYPYKASDETCDTNKENAPVVSIDGHEDVPKNSEDDLMKAVANQPVSVAIDAGGSDFQFYSEG

VFTGRCGTELNHGVAVVGYGTTIDGTKYWIVKNSWGEEWGEKGYIRMQRGIRHKEGLCGIAMEASYPL
KNSNTNPSRLSLDSLKDEL

>AT4G35350.1 XCP1

MAFSAPSLSKFSLLVAISASALLCCAFARDFSIVGYTPEHLTNTDKLLELFESWMSEHSKAYKSVEEKVHR
FEVFRENLMHIDQRNNEINSYWLGLNEFADLTHEEFKGRYLGLAKPQFSRKRQPSANFRYRDITDLPKS
VDWRKKGAVAPVKDQGQCGSCWAFSTVAAVEGINQITTGNLSSLSEQELIDCDTTFNSGCNGGLMDYA
FQYIISTGGLHKEDDYPYLMEEGICQEQKEDVERVTISGYEDVPENDDESLVKALAHQPVSVAIEASGRD
FQFYKGGVFNGKCGTDLDHGVAAVGYGSSKGSDYVIVKNSWGPRWGEKGFIRMKRNTGKPEGLCGIN
KMASYPTKTK

>AT1G20850.1 XCP2

MALSSPSRILCFALALSAASLSLSFASSHDYSIVGYSPEDLESHDKLIELFENWISNFEKAYETVEEKFLRF
EVFKDNLKHIDETNKKGKSYWLGLNEFADLSHEEFKKMYLGLKTDIVRRDEERSYAEFAYRDVEAVPKS
VDWRKKGAVAEVKNQGSCGSCWAFSTVAAVEGINKIVTGNLTTLSEQELIDCDTTYNNGCNGGLMDYAF
EYIVKNGGLRKEEDYPYSMEEGTCEMQKDESETVTINGHQDVPTNDEKSLLKALAHQPLSVAIDASGRE
FQFYSGGVFDGRCGVDLDHGVAAVGYGSSKGSDYIIVKNSWGPKWGEKGYIRLKRNTGKPEGLCGINK
MASFPTKTK

>AT1G09850.1 XBCP3

MSMSSSSFISLTFFFLLLVSSSSSSDDISELFDDWCQKHGKTYGSEEERQQRIQIFKDNHDFVTQHNLIT
NATYSLSLNAFADLTHHEFKASRLGLSVSAPSVIMASKGQSLGGSVKVPDSVDWRKKGAVTNVKDQGS
CGACWSFSATGAMEGINQIVTGDLISLSEQELIDCDKSYNAGCNGGLMDYAFEFVIKNHGIDTEKDYPYQ
ERDGTCKKDKLKQKVVTIDSYAGVKSNDEKALMEAVAAQPVSVGICGSERAFQLYSSGIFSGPCSTSLD
HAVLIVGYGSQNGVDYWIVKNSWGKSWGMDGFMHMQRNTENSDGVCGINMLASYPIKTHPNPPPPSP
PGPTKCNLFTYCSSGETCCCARELFGLCFSWKCCEIESAVCCKDGRHCCPHDYPVCDTTRSLCLKKTG
NFTAIKPFWKKNSSKQLGRFEEWVM

>AT1G06260.1 THI1

MLNVLRNSNLTLAVLICFVLIASKLCSVDSSVYDPHKTLKQRFEKWLKTHSKLYGGRDEWMLRFGIYQSN
VQLIDYINSLHLPFKLTDNRFADMTNSEFKAHFLGLNTSSLRLHKKQRPVCDPAGNVPDAVDWRTQGAV
TPIRNQGKCGGCWAFSAVAAIEGINKIKTGNLVSLSEQQLIDCDVGTYNKGCSGGLMETAFEFIKTNGGL
ATETDYPYTGIEGTCDQEKSKNKVVTIQGYQKVAQNEASLQIAAAQQPVSVGIDAGGFIFQLYSSGVFTN

YCGTNLNHGVTVVGYGVEGDQKYWIVKNSWGTGWGEEGYIRMERGVSEDTGKCGIAMMASYPLQ

>AT5G45890.1 SAG12

MALKHMQIFLFVAIFSSFCFSITLSRPLDNELIMQKRHIEWMTKHGRVYADVKEENNRYVVFKNNVERIEH
LNSIPAGRTFKLAVNQFADLTNDEFRSMYTGFKGVSALSSQSQTKMSPFRYQNVSSGALPVSVDWRKK
GAVTPIKNQGSCGCCWAFSAVAAIEGATQIKKGKLISLSEQQLVDCDTNDFGCEGGLMDTAFEHIKATGG
LTTESNYPYKGEDATCNSKKTNPKATSITGYEDVPVNDEQALMKAVAHQPVSVGIEGGGFDFQFYSSGV
FTGECTTYLDHAVTAIGYGESTNGSKYWIIKNSWGTKWGESGYMRIQKDVKDKQGLCGLAMKASYPTI

>AT2G34080.1 PAP1

MASIMVLVTVLIILFTGFRISQATSRTVIFREQSMVDKHEQWMARFSREYRDELEKNMRRDVFKKNLKFIE
NFNKKGNKSYKLGVNEFADWTNEEFLAIHTGLKGLTEVSPSKVVAKTISSQTWNVSDMVVESKDWRAE
GAVTPVKYQGQCGCCWAFSAVAAVEGVAKIAGGNLVSLSEQQLLDCDREYDRGCDGGIMSDAFNYVV
QNRGIASENDYSYQGSDGGCRSNARPAARISGFQTVPSNNERALLEAVSRQPVSVSMDATGDGFMHY
SGGVYDGPCGTSSNHAVTFVGYGTSQDGTKYWLAKNSWGETWGEKGYIRIRRDVAWPQGMCGVAQY
AFYPVA

>AT1G29090.1 PAP2

MCDSFINNKMTSILFMLVSLTILSMNLKVSQATSRVTFHEPIVAEHHQQWMTRFSRVYSDELEKQMRFDV
FKKNLKFIEKFNKKGDRTYKLGVNEFADWTREEFIATHTGLKGVNGIPSSEFVDEMIPSWNWNVSDVAG
RETKDWRYEGAVTPVKYQGQCGCCWAFSSVAAVEGLTKIVGNNLVSLSEQQLLDCDRERDNGCNGGI
MSDAFSYIIKNRGIASEASYPYQAAEGTCRYNGKPSAWIRGFQTVPSNNERALLEAVSKQPVSVSIDAD
GPGFMHYSGGVYDEPYCGTNVNHAVTFVGYGTSPEGIKYWLAKNSWGETWGENGYIRIRRDVAWPQ
GMCGVAQYAFYPVA

>AT1G29080.1 PAP3

MDFVEFVCVVLTIFFMDLKISEATSRVALYKPSSIVDYHQQWMIQFSRVYDDEFEKQLRLQVLTENLKFIE
SFNNMGNQSYKLGVNEFTDWTKEEFLATYTGLRGVNVTSPFEVVNETKPAWNWTVSDVLGTNKDWRN
EGAVTPVKSQGECGGCWAFSAIAAVEGLTKIARGNLISLSEQQLLDCTREQNNGCKGGTFVNAFNYIIKH
RGISSENEYPYQVKEGPCRSNARPAILIRGFENVPSNNERALLEAVSRQPVAVAIDASEAGFVHYSGGVY
NARNCGTSVNHAVTLVGYGTSPEGMKYWLAKNSWGKTWGENGYIRIRRDVEWPQGMCGVAQYASYP
VA

>AT2G27420.1 PAP4

MASTIIFILTIFLSYRTSLATSRGSLFEASAIEKHEQWMARFNRVYSDETEKRNRFNIFKKNLEFVQNFNM
NNKITYKVDINEFSDLTDEEFRATHTGLVVPEAITRISTLSSGKNTVPFRYGNVSDNGESMDWRQEGAVT
PVKYQGRCGGCWAFSAVAAVEGITKITKGELVSLSEQQLLDCDRDYNQGCRGGIMSKAFEYIIKNQGITT
EDNYPYQESQQTCSSSTTLSSSFRAATISGYETVPMNNEEALLQAVSQQPVSVGIEGTGAAFRHYSGG
VFNGECGTDLHHAVTIVGYGMSEEGTKYWVVKNSWGETWGENGYMRIKRDVDAPQGMCGLAILAFYP
LA

>AT3G49340.1 PAP5

MTSIVFFLLAILLSSRTSGVTSRGGLFEASAVEKHEQWMSRFNRVYSDDSEKTSRFEIFTNNLKFVESIN
MNTNKTYTLDVNEFSDLTDEEFKARYTGLVVPEGMTRISTTDSHETVSFRYENVGETGESMDWIQEGAV
TSVKHQQQCGCCWAFSAVAAVEGMTKIANGELVSLSEQQLLDCSTENNGCGGGIMWKAFDYIKENQGI
TTEDNYPYQGAQQTCESNHLAAATISGYETVPQNDEEALLKAVSQQPVSVAIEGSGYEFIHYSGGIFNGE
CGTQLTHAVTIVGYGVSEEGIKYWLLKNSWGESWGENGYMRIMRDVDSPQGMCGLASLAYYPVA

>AT4G39090.1 RD19A

MDRLKLYFSVFVLSFFIVSVSSSDVNDGDDLVIRQVVGGAEPQVLTSEDHFSLFKRKFGKVYASNEEHDY
RFSVFKANLRRARRHQKLDPSATHGVTQFSDLTRSEFRKKHLGVRSGFKLPKDANKAPILPTENLPEDF
DWRDHGAVTPVKNQGSCGSCWSFSATGALEGANFLATGKLVSLSEQQLVDCDHECDPEEADSCDSGC
NGGLMNSAFEYTLKTGGLMKEEDYPYTGKDGKTCKLDKSKIVASVSNFSVISIDEEQIAANLVKNGPLAV
AINAGYMQTYIGGVSCPYICTRRLNHGVLLVGYGAAGYAPARFKEKPYWIIKNSWGETWGENGFYKICK
GRNICGVDSMVSTVAATVSTTAH

>AT2G21430.1 RD19B

MDYHLRVLFSVSLIFVFVSVSVCGDEDVLIRQVVDETEPKVLSSEDHFTLFKKKFGKVYGSIEEHYYRFS
VFKANLLRAMRHQKMDPSARHGVTQFSDLTRSEFRRKHLGVKGGFKLPKDANQAPILPTQNLPEEFDW
RDRGAVTPVKNQGSCGSCWSFSTTGALEGAHFLATGKLVSLSEQQLVDCDHECDPEEEGSCDSGCNG
GLMNSAFEYTLKTGGLMREKDYPYTGTDGGSCKLDRSKIVASVSNFSVVSINEDQIAANLIKNGPLAVAIN
AAYMQTYIGGVSCPYICSRRLNHGVLLVGYGSAGFSQARLKEKPYWIIKNSWGESWGENGFYKICKGR
NICGVDSLVSTVAATTS

>AT4G16190.1 RD19C

MDRVVFFFLIAATLLAGSLGSTVISGEVTDGFVNPIRQVVPEENDEQLLNAEHHFTLFKSKYEKTYATQVE
HDHRFRVFKANLRRARRNQLLDPSAVHGVTQFSDLTPKEFRRKFLGLKRRGFRLPTDTQTAPILPTSDL
PTEFDWREQGAVTPVKNQGMCGSCWSFSAIGALEGAHFLATKELVSLSEQQLVDCDHECDPAQANSC
DSGCSGGLMNNAFEYALKAGGLMKEEDYPYTGRDHTACKFDKSKIVASVSNFSVVSSDEDQIAANLVQ
HGPLAIAINAMWMQTYIGGVSCPYVCSKSQDHGVLLVGFGSSGYAPIRLKEKPYWIIKNSWGAMWGEH
GYYKICRGPHNMCGMDTMVSTVAAVHTSPK

>AT3G54940.2 RD19D

MVAKALAQLITCIILFCHVVASVEDLTIRQVTADNRRIRPNLLGTHTESKFRLFMSDYGKNYSTREEYIHRL
GIFAKNVLKAAEHQMMDPSAVHGVTQFSDLTEEEFKRMYTGVADVGGSRGGTVGAEAPMVEVDGLPE
DFDWREKGGVTEVKNQGACGSCWAFSTTGAAEGAHFVSTGKLLSLSEQQLVDCDQACDPKDKKACD
NGCGGGLMTNAYEYLMEAGGLEEERSYPYTGKRGHCKFDPEKVAVRVLNFTTIPLDENQIAANLVRHG
PLAVGLNAVFMQTYIGGVSCPLICSKRNVNHGVLLVGYGSKGFSILRLSNKPYWIIKNSWGKKWGENGY
YKLCRGHDICGINSMVSAVATQVSS

>AT5G60360.1 AALP

MSAKTILSSVVLVVLVAASAAANIGFDESNPIRMVSDGLREVEESVSQILGQSRHVLSFARFTHRYGKKY
QNVEEMKLRFSIFKENLDLIRSTNKKGLSYKLGVNQFADLTWQEFQRTKLGAAQNCSATLKGSHKVTEA
ALPETKDWREDGIVSPVKDQGGCGSCWTFSTTGALEAAYHQAFGKGISLSEQQLVDCAGAFNNYGCN
GGLPSQAFEYIKSNGGLDTEKAYPYTGKDETCKFSAENVGVQVLNSVNITLGAEDELKHAVGLVRPVSIA
FEVIHSFRLYKSGVYTDSHCGSTPMDVNHAVLAVGYGVEDGVPYWLIKNSWGADWGDKGYFKMEMGK
NMCGIATCASYPVVA

>AT3G45310.1 ALP2

MSVKLNLSSSILLILFAAAASKEIGFDESNPIKMVSDNLHELEDTVVQILGQSRHVLSFSRFTHRYGKKYQ
SVEEMKLRFSVFKENLDLIRSTNKKGLSYKLSLNQFADLTWQEFQRYKLGAAQNCSATLKGSHKITEATV
PDTKDWREDGIVSPVKEQGHCGSCWTFSTTGALEAAYHQAFGKGISLSEQQLVDCAGTFNNFGCHGG
LPSQAFEYIKYNGGLDTEEAYPYTGKDGGCKFSAKNIGVQVRDSVNITLGAEDELKHAVGLVRPVSVAFE
VVHEFRFYKKGVFTSNTCGNTPMDVNHAVLAVGYGVEDDVPYWLIKNSWGGEWGDNGYFKMEMGKN
MCGVATCSSYPVVA

>AT1G02300.1 CTB1

MADSCCIRLHLLASVFLLLFSSFNLQGIAAENLSKQKLTSLILQNEIVKEVNENPNAGWKAAFNDRFANAT
VAEFKRLLGVIQTPKTAYLGVPIVRHDLSLKLPKEFDARTAWSHCTSIRRILVGYILNNVLLWSTITLWFWFL
LGHCGSCWAFGAVESLSDRFCIKYNLNVSLSANDVIACCGLLCGFGCNGGFPMGAWLYFKYHGVVTQE
CDPYFDNTGCSHPGCEPTYPTPKCERKCVSRNQLWGESKHYGVGAYRINPDPQDIMAEVYKNGPVEV
AFTVYEDFAHYKSGVYKYITGTKIGGHAVKLIGWGTSDDGEDYWLLANQWNRSWGDDGYFKIRRGTNE
CGIEQSVVAGLPSEKNVFKGITTSDDLLVSSV

>AT1G02305.1 CTB2

MADNCIRLLHSASVFFCLGLLISSFNLLQGIAAENLSKQKLTSWILQNEIVKEVNENPNAGWKASFNDRFA
NATVAEFKRLLGVKPTPKTEFLGVPIVSHDISLKLPKEFDARTAWSQCTSIGRILDQGHCGSCWAFGAVE
SLSDRFCIKYNMNVSLSVNDLLACCGFLCGQGCNGGYPIAAWRYFKHHGVVTEECDPYFDNTGCSHP
GCEPAYPTPKCARKCVSGNQLWRESKHYGVSAYKVRSHPDDIMAEVYKNGPVEVAFTVYEDFAHYKSG
VYKHITGTNIGGHAVKLIGWGTSDDGEDYWLLANQWNRSWGDDGYFKIRRGTNECGIEHGVVAGLPSD
RNVVKGITTSDDLLVSSF

>AT4G01610.1 CTB3

MAVYNTKLCLASVFLLLGLLLAFDLKGIEAESLTKQKLDSKILQDEIVKKVNENPNAGWKAAINDRFSNAT
VAEFKRLLGVKPTPKKHFLGVPIVSHDPSLKLPKAFDARTAWPQCTSIGNILDQGHCGSCWAFGAVESLS
DRFCIQFGMNISLSVNDLLACCGFRCGDGCDGGYPIAAWQYFSYSGVVTEECDPYFDNTGCSHPGCEP
AYPTPKCSRKCVSDNKLWSESKHYSVSTYTVKSNPQDIMAEVYKNGPVEVSFTVYEDFAHYKSGVYKHI
TGSNIGGHAVKLIGWGTSSEGEDYWLMANQWNRGWGDDGYFMIRRGTNECGIEDEPVAGLPSSKNVF
RVDTGSNDLPVASV

>Solyc02g076730.4.1

MRVKGTYLLFFLFLILAGLSSQVYSISSDFSILNRSNEFISDESVFQLFQEWKQKHGKVYKDEKEEEMRL
EKFRWNVKYIVEKNSERKSASEHFVGLTNFADMSNEEFREVHGSKIKIPFNKRNIIQMKNVEEKPTSISC
DAPRSRDWRKHGAVTEVKNQERCGACWAFSACGAVEGINAIITGELISLSVQELVNCDNSTNTGCYGG
YMDHAFEWVISNGGIASELDYPYTSSQGACKITKVNHKVVTIDGYRDVPQEETALLCAVAQQPVSVGIDG
TNVDFQLYRGGIYDGSCSSSPDDLNHGVLIVGYGSEGEDDYWIIKNSWGTSWGVEGYGYIRRNSDLPY
GVCAINSLASYPTKELSFELSPYPSPAVQPPPPPPFPLPSPPSSAVPPPSPSPYPPPPPSASSPSPSPSPP
SEPSPYPSPAIPPSTPPSSPPPSAPFPYPSPIVPPPPPPSPPLSPDVPLFPPPPSPQPPFAPYPYPSPTVP
PSPPPSPPSHPPPSPYPFPAVPPPPSPLPPLPPSPSPPPPPPPPPPPSPPPPSPSPPPPPPSPPPPSSSP
PPSPPLPSHSPPPPPPSPPPPSPSPPPPPPPPPPSPPPPSPSPPPPSPPPPSPSPPPPPPPPSPPPPPPSP

PPPSPSPPPPPPSPPPPPPPSPPPPPPSPPPPSPPPPSPPPPPPPPPPSPPPSPPPPSPLHPPHHLLHH
PLHHLLRHPLPPLHHLILSQVNVETGIIVLNMKHVVVSCISLAYASVISVVLMRMVFAVMDQTIAAHLNFPF
VMFMKASVSRGLMMLWELQQRREEWPNTSYHGVQAVKKQRRWAKL

>Solyc04g082710.5.1

MALFSFTKFSLLVLFSISLLACTSLARDHSIVGYSSDDLTCIDKLINLFDKWMDKHGKIYKSIEEKLHRFEIF
KENLKHIDERNKMVTNYWLGLNEFSDLSHDEFKEMYLGLKVDQELLNKREKLSQEEFAYRDFVDLPKSV
DWRKKGAVTPVKNQGQCGSCWAFSTVAAVEGINQIKTGNLTSLSEQELIDCDTTYNNGCNGGLMDYAF
QFIVSNGGLHKEDDYPYLMEEGTCDEKRDESEVVTIDGYHDVPANDEQSLLKALANQPLSVAIEASTRD
FQFYSGVNILIHSFSL

>Solyc07g041900.4.1

MSRLSLVLILVAGLFATALAGPATFADKNPIRQVVFPDELENGILQVVGQTRSALSFARFAIRHRKRYDSVE
EIKQRFEIFLDNLKMIRSHNRKGLSYKLGINEFTDLTWDEFRKHKLGASQNCSATTKGNLKLTNVVLPETK
DWRKDGIVSPVKAQGKCGSCWTFSTTGALEAAYAQAFGKGISLSEQQLVDCAGAFNNFGCNGGLPSQ
AFEYIKFNGGLDTEEAYPYTGKNGICKFSQANIGVKVISSVNITLGAEYELKYAVALVRPVSVAFEVVKGFK
QYKSGVYASTECGDTPMDVNHAVLAVGYGVENGTPYWLIKNSWGADWGEDGYFKMEMGKNMCGVAT
CASYPIVA

>Solyc07g041920.5.1

MSRSSVLLVLIAGLFAATIAGAATFADENPIRQVVVSEEVESGILQVVGESDDALSFARFVNKYGKKYDSV
EETKLRFEIFVENLKMIRSHNNKRSLVSYKLGVNKFTDLTWDEFRRVKLQGTPQNCSATTKTNLKFTNGL
LPEKKDWRETGIVSPVKAQGDCGACWTFSTTGALEAAYAQAFGKGISLSEQQLVDCAGAFDNHGCNG
GLPSHAFEYIKYNGGLETEESYPYTAKDGICKFKPQNVAVKVIDSVNITEGAEDELKIAVAFIRPVSVAFEVI
DGFKQYESGVYTSTACGNTSMDVNHAVLAVGYGVENGVPYWLIKNSWGADWGDNGYFKMEMGKNM
CGVATCASYPIVA

>Solyc07g053460.5.1

MKPSYITFSNFMLLIFLTLCYVTNASQDDPIILNRYQNWVKKYRRKYQNEAEWNMRFGIYQSNIQFIDFFN
SLNLSYSLTDNAFADMTNREFNSIYLGYEKPIQEQKDINNVTSYDISTLPIGVDWRKDGVVTPIKDQKSCG
SCWAFSAVAAIEGINKIKTGKLVSLSEQQLMDCDVYSDNQGCNGGFMESAFDYIMENGGITTSKNYPYIG
KEQKCNTKKAKQHEVRISNYEKVPPNEESLQVAINKQPISVAIDASGYNFQLYSRGVFSGYCGNMLNHA
VTLIGYDVEENNGEKYWIVKNSWGTMWGESGYMKIKRGSSDNGGMCGIAMQASYPLMEEYV

>Solyc02g069090.4.1

MKHIATFLLLVSVSTLVLQVVAENPISQAKAESAILQDSIVKQVNENEKAGWRAALNPQFSNFTVSQFKRL
LGVKPTRKGDLKGIPILTHPKLLKLPQEFDARVAWPQCSTIGRILDQGHCGSCWAFGAAESLSDRFCIHY
GLNISLSANDIIACCGYLCGDGCDGGYPLEAWKYFVRKGVVTEECDPYFDNKGCSHPGCEPGYPTPQC
KRKCVKENLLWSKSKHFGINAYLINSDPYSIMTEVYKNGPVEVSFTVYEDFAHYKSGVYKHINGEEMGG
HAVKLIGWGTSEDGEDYWLLANQWNRGWGDDGYFKIRRGTNECGIEEEVVAGMPSAKNLNVELDVSD
ALLDASM

>Solyc02g069100.5.1

MALTLKSLITPLLFGAFFILILQQVAAEKPITEAKLESAILQDSIVKQVNENAEAGWKAAFNPQLSNFTVSQF
KRLLGVKPAREGDLEGIPVLTHPKLKELPKEFDARKAWPQCSTIGRILGQDIAVLVGLLVLLSRCLIVSVSIII
CISLSVNDLLACCGFLCGSGCDGGYPIAAWRYFKRRGVVTEECDPYFDTTGCSHPGCEPLYPTPKCHR
KCVKGNVLWRKSKHYGVNAYRVSHDPQSIMAEVYKNGPVEVSFTVYEDFAHYKSGVYKHVTGGNMGG
HAVKLIGWGTSEQGEDYWLIANSWNRGWGEDGYFKIRRGTNECGIEHSVVAGLPSARNLNVELGDAVL
DASM

>Solyc02g069110.5.1

MGMNKMFLPTPLLLCAFFIFILQVVAEKPISEAKGESVILRESIIKEVNENGKAGWKAAFNPRFSNFTVSQ
FKRLLGVKPPREGDLKSIPILTHPKLKNLPKEFDARTAWSECSTIGRILGQFLHLIGHCGSCWAFGAVESL
SDRFCIHYGLNISLSVNDVIACCGFHCGNGCDGGSPIAAWHYFIRKGVVSEKCDPYFDNIGCSHPGCEP
TYPTPQCNRKCVNENLLWSKSKHFGVNAYMISSNPYSIMTEVYKNGPVEVALNVYEDFAHYKSGVYKH
VTGEYIGGHAVKLIGWGTSEEGEDYWLLVNSWNKGWGNDGYFKIRRGTNECDIESNVVAGLPSARNLN
VELDDDFLDTSM

>Solyc01g107760.5.1

MTYFRPLFLFLLTFFVLSSALDMSIISYDEKHADLGATNHRTDDEVKGLYESWIVKHGKNYNAIGEKEKRF
EIFKDNLRFIDEQNAETRPYKLGLNRFSDLTNDEYRALFVGGRFDKKTRLLKNPKSERYAFKAGEKLPES
VDWRQKGAVAPVKDQGQCGSCWAFSTVGAVEGINQIVTGELISLSEQELVDCDKSYNQGCNGGLMDY
AFEFIKNNGGIDTEADYPYRAKDGTCDSNRKNARVVSIDGYEDVPINDEKSLMKALSNQPVSVAIEAGGR
AFQHYSSGVFTGYCGTQLDHGVVAIGYGTDNGSDYWIVRNSWGPNWGESGYIRLERNLANSTSGKCG
IAMEPSYPLKNGANPPNPGPSPPSPVAPSTVCDEYYSCTAGTTCCCIYKYGDYCFGWGCCPYESATCC

DDNNSCCPHDYPVCDVNSGTCQMSKDSPLSVKALKRGPATARVNWSGMKSNRKVSYD

>Solyc04g078540.5.1

MAIHLSALTISILVMVVSSAAVTSAAAEDMSIISYNEKHHTIGAGRTDDEVMSMYESWLVEHKKVYNALGE
KDKRFQIFKDNLKYIDEHNAMPEKSYKLGLTKFADLTNEEYRSVYLGTKPDASRRLSSRQSDRYAPKVG
DRLPESVDWVKKGVLVGVKDQGQCGSCWAFSAVAAIEAVNKIKTGDSISLSEQELVDCDTSSNNGCDG
GLMDYAFEFVIKNGGLDTEEDYPYTGEDGRCDLTRKNAKVVTIDGYEDVPANDENAMKKAIASQPVSVAI
EAGGKDFQHYKSGIFTGKCGAAVDHGVVAVGYGSENGMDYWIVRNSWGASWGEHGYLRMQRNIANP
KGLCGIATVVSYPVKTGQNPPKPAPSPPSPVKPPTTCDDMYSCPSGTTCCCVYEYYHMCFAWGCCPM
EGATCCKDHNSCCPHDYPVCNVKAGTCSISENNPLSVKAMSHILAKPIGSFSNQGMKNTIS

>Solyc04g080873.2.1

MKWLLPSLVLVLLIFQQPLCTCSSISDLFETWCQQNGKKYSSEQERMYRFKVFEENYAYITEHNSKGNS
SYTLGLNAYSDLTHHEFRNSFLGLSSSANDFIRLKGRGSGSSAAGVLSDVDAPSSLDWRDKGAVTNVK
NQGSCGACWSFSATGAIEGINKITTGSLVSLSEQELIDCDRSYNQGCGGGLMDYAFEFVIKNGGIDTEKD
YPFREKEGTCNKNKLQRRVVTIDGYTDIPQNDEDKLLKAVATQPVSVGICGSARAFQSYSKGIFTGPCPT
DLDHAVLIVGYGSENGFDYWIIKNSWGTSWGINGYIHMQRNSGNQEGICGVNKLASYPTKTSPNPPNPP
APGPSKCSTFTSCGQGETCCCGLKFLGICLSWKCCGLDSAVCCKDGRHCCPWDYPICDTSRNLCLKR
MSNATIVQQPQKEPFTGKFGGLIYPF

>Solyc01g110110.3.1

MAHRFSLVFVLSILLTTSFLLAVNGEIKGGDDDILIRQVVGDEDHHMLNAEHHFTLFKKRFGKTYASDEEH
HYRFSVFKANLRRAMRHQKLDPSAVHGVTQFSDMTPDEFSQKFLGVNRRLRFPSDANKAPILPTEDLP
SDFDWREHGAVTPVKNQGSCGSCWSFSTTGALEGANFLATGKLVSLSEQQLVDCDHECDPEEKDSCD
SGCSGGLMNSAFEYTLKAGGLMREEDYPYTGTDKATCKFDNTKVAAKVANFSVVSLDEEQIAANLVKN
GPLAVAINAVFMQTYVGGVSCPYICSKQLDHGVLLVGYGTGFSPIRMKEKPYWIIKNSWGEKWGESGYY
KICRGRNVCGVDSMVSTVAAVSTSSW

>Solyc02g076690.3.1

MENTQKSYLFLLLSFPLFLILATLSSQVSAFTTYFPELDKPQELLTEERVFQLFQEWKQKHGKIYKNEKEE
ERRLENFKRNVKYIVDKNSKRRSESDHLVGLNNFADMSNEEFSQVHTSKIKMPFKQQNKTTISANSCDA
PPAKDWRKHGVVTEVKNQGACGCCWAFSACGAIEGINALVTGELISLSTQELVNCDTSNKGCEGGLMD
PAFKFVINNRGIDSAADYPYTKSRGSCSYNKLNKKAVTIDGYQDVAQEESALLCAVARQPVSVGIDGKSL

DFQLYAGGIYDGECSSNPDDLSHAVLIVGYGSEGGVDYWIIKNSWGKSWGMEGYAYIKRNTVLPYGICGI
NSLASYPMKESSSAPPSPPKPNICEDGLHYCPEGQTCCCGLDFFGKCLVHGCCPIENGVCCENSRLCC
PQDFPYCDVLQGLCHKDYGDKIGVAARKRTMAKLKLTWSSATKEIDEMDQTFQWNRRNQFAVMR

>Solyc02g076710.3.1

MGSKQSPPLFLFLIPLFLIFGALSSQVSSFTTDFPILERPTEPLSEETVFQLFQEWKQKHGKVYMNKKEE
EMKLENFKRNVKYIVEKNSKRKSDSDYLVGLTKFADMSNEEFRQVHTSKIKIPFNKRKTIRMKVAEKETT
SFSCDAPPSMDWRKHGAVTKVKDQGQCGACWAFSASGAMEGINAIVAGELISLSEQELIDCDTSNNSG
CKGGLMDPAFEWVINNGGIDSAADYAYTAHSQGHCNYNKVNHKVVTIDGYRDVPKEESALLCAAAQQP
VSVAIDGSSPDFQLYLGGIYDGECSDDPNKVSHGVVIVGYDSDGYDDYWIIKNSWGTEWGMEGYGYIR
RNTNLPYGVCAINSLASYPTKESSPSPYPSPAMPPPPPPSPPPPTPKPSECGDFSYCPGDQTCCCVLE
FSGLCLEQGCCPYENGVCCDGSNYCCPADYPICDVYDGLCLKDHGDKIGVEARKRRMAKYNLPWRITE
ATEEMSQTLKWKRNHVAAMR

>Solyc02g076910.3.1

MTIFTNRSKYICLALFFIVFGLWSSQLASSRPINNETTMRVRHEQWIAHHDKIYNDLKEKEIRFKIFKENVE
RIETFNAGEDKGYKLGVNKFADLTNEEFRVLHTGYKSSSHPKIMSSSKPKTHFRYANITDVPPIMDWRRK
GAVTPIKDQMECGCCWAFSAVAAMEGLHQLKTGKLIPLSEQELVDCDVEGEDLGCTGGLLDTAFQFIIKN
KGLTTEANYPYQAADGVCNKKKSALSVAKITGYEDVPANNEKALLQAVANQPVSVAIDGSSFDFQFYSS
GVFSGSCSTWLNHAVTAVGYGAASDGTKYWIIKNSWGSKWGENGYAHMKRDIDDKKGLCGLAMKASY
PTA

>Solyc02g076970.2.1

MALTINWKSAFLALAVLTMWTIEVTSRELNEASMVQKHEKWMARFGRVYRDDAEKAKRYEDVTAPATM
DWRKKGAVTGVKDQGQCGCCWAFSAVAATEGINEIKTGKLISLSEQELVDCDTSSDMGCEGGLMDDAF
KFIIKNHGLTTESNYPYEGTDGTCKTGKKSNGAAKITGYEDVPANSESSLLSAVANQPVSVAIDASGSDF
QFYSSGVFTGECGTELDHGVTAVGYGITSDGTKYWLVKNSWGTSWGENGYIRMQRGIDAKEGICGIAM
QASYPTA

>Solyc02g076980.5.1.1 RCR3

MQASYNNILFPSTLTMAMKVDLMNILITLFFVISMFNTQTRGRSQPKLSVSERHELWMSRHGRVYKDEV
EKGERFMIFKENMKFIESVNKAGNLSYKLGMNEFADITSQEFLAKFTGLNIPNSYLSPSPMSSTEFKKIND
LSDDYMPSNLDWRESGAVTQVKHQGRCGCCWAFSAVGSLEGAYKIATGNLMEFSEQELLDCTTNNYG

CNGGFMTNAFDFIIENGGISRESDYEYLGQQYTCRSQEKTAAVQISSYQVVPEGETSLLQAVTKQPVSIG
IAASQDLQFYAGGTYDGNCADRINHAVTAIGYGTDEEGQKYWLLKNSWGTSWGENGYMKIIRDSGDPS
GLCDIAKMSSYPNIA

>Solyc02g077040.4.1 Pip1

MASNFFLKNITVVLLLFSILSLYPFIVTSRNLKELSMLERHENWMVHHGRVYKDDIEKEHRFKTFKENVEFI
ESFNKNGTQRYKLAINKYADLTTEEFTTSFMGLDTSLLSQQESTATTTSFKYDSVTEVPNSMDWRKRGS
VTGVKDQGVCGCCWAFSAAAAIEGAYQIANNELISLSEQQLLDCSTQNKGCEGGLMTVAYDFLLQNNG
GGITTETNYPYEEAQNVCKTEQPAAVTINGYEVVPSDESSLLKAVVNQPISVGIAANDEFHMYGSGIYDG
SCNSRLNHAVTVIGYGTSEEDGTKYWIVKNSWGSDWGEEGYMRIARDVGVDGGHCGIAKVASFPTV

>Solyc02g093040.4.1

MAKTLITTLLLALFSSLSYAIDMSIIDHKNKEGILYEKWLAEHGKIYNALGEKEKRFEIFKDNLRFIEEYNASE
NRTFKVGLNQFADLTNDEYRAVYLGTKSDARRRLVKSKNTSQRYASPPNELLPHSVDWRKKGAVAPIKN
QGSCGSCWAFSTVAAVEGINQIVTGEMITLSEQELVDCDRSQNGGCNGGLMDYAFEFIISNGGMDTEN
HYPYRGVDGRCDPIRKNSKVVSIDDYEDVPRNEKALQKAVAHQPVSVAIEASGKAFQLYTSGVFTGDCG
EKVDHGVVVVGYGSEDGNDYWLVRNSWGTKWGESGYVKMERNVKNGHLGKCGIMTEASYPIKEAINK
RIITTSNSNEEMISSI

>Solyc03g006200.3.1

MAYMFNSSLIFATLLVLNFLIIQATSRTLYESSMVEKHEQWMAKYGREYKDEIEKAERFKVFKQNFEYIESI
NKNGTRSYKLGINEFADRRKEEFKSVRNGYKIPSKQRIKTKSFRYENASSPITMDWRKKGAVTRIKDQG
QCGCCWAFSAVAAIEGLNMIKTGKLISLSEQELVDCDIGLNEGCEGGLMDNAFKFIIKNNGITSENSYPYK
GIDSSCNKNKLLNHMVKISRYEDIPSNSEWALLKAIANQPVSVAIDAGGSDFQFYSSGVFTGHCGNQLD
HGVTAVGYGVTKGGTKYWLVKNS

>Solyc03g006210.2.1

MALVLEWKTQFTLLFMIVGMYASQVTCSQDSTSMVEKHESWMARHGRTYKNDIEKAKRLNIFTKNVKFI
ESFNNNDSKSYKLGINKFTDLTSEEFMRYYTTNHGLNSKFSSIKSQKLSPTTISSFKYENISDVPSEMDW
RKSGVVTSIKDQGQCGCCWAFSAVAALEGANKLSTGKLISLSEQQLLDCSTENNGCNGGLITMAYDYIV
KNNGIAEESSYPYEENQDSCKIQDSIVKMSSYETLPPSNEPMLLTAVARQPISVGIAVNEEFKLYKSGVYD
GNCGDEVNHAVTIIGYGTSNENGTKYWLIKNSWGSSWGENGYMKIARDIGNNDGLCRIATMASYPIV

>Solyc03g044230.1.1

MSIVVITTFLLIFFVYLINANDMFYNNNNNNNNDTDEEVKRIYEIWLAEHGKIYNGLGEQGKRFEIFKDNLRF
IEEHNNSKNQTYVLGLNRFADLTNEEYRTIFLGTKSDARRRLVESKNASHHYDFRASDSVPKSVDWRKK
GAVAPIKDQGTCGSCWAFSTVAAVEGINQIATGEMITLSEQELIDCDRMYNDGCNGGLMDYAFQFIISNG
GINTESHYPYNGIDHICDLVQKNAKVVSIDDYEVVAANEKALKKAVAHQPISVAIEASGRAFQLYSSGIFTG
NCGTQLDHGVVVVGYGTENGVDYWIVRNSWGTNWGEDGYIKMERNIEDTNSGKCGIAMEGSYPIKNAI
NKITMGEEGLQEVSKEITWHGSCLFSEF

>Solyc03g111730.3.1

MKKLFLVLFTLALVLRLGESFDFHEKELETEEKFWELYERWRSHHTVSRSLDEKHKRFNVFKANVHYVH
NFNKKDKPYKLKLNKFADMTNHEFRQHYAGSKIKHHRTLLGASRANGTFMYANEDNVPPSIDWRKKGA
VTPVKDQGQCGSCWAFSTVVAVEGINQIKTKKLVSLSEQELVDCDTTENQGCNGGLMDPAFDFIKKRG
GITTEERYPYKAEDDKCDIQKRNTPVVSIDGHEDVPPNDEDALLKAVANQPISVAIDASGSQFQFYSEGV
FTGECGTELDHGVAIVGYGTTVDGTKYWIVKNSWGAEWGEKGYIRMQRKVDAEEGLCGIAMQPSYPIK
TSSNPTGSPAATPKDEL

>Solyc04g080960.4.1

MLNAEHHFSLFKAKFGKIYASQEEHDHRLKVFKANLHRAKRHQLLDPSAEHGITQFSDLTPSEFRRTYLG
LNKPRPNLNAEKAPILPTKDLPSDFDWREKGAVTDVKNQGSCGSCWSFSTTGAVEGAHFLATGELVSL
SEQQLVDCDHECDPVEKNDCDAGCNGGLMTTAFEYTLKAGGLQLEKDYPYTGRNGKCHFDKSRIAAS
VSNFSVVGLDEDQIAANLLKHGPLAVGINAAWMQTYVRGVSCPLICFKRQDHGVLLVGYGSEGFAPIRL
KNKPYWIIKNSWGKTWGEHGYYKICRGHNICGVDAMVSTVTATHTTNPNL

>Solyc05g013920.4.1

MEAWKKIFLISLSLAMIFGLVNSLEFTEKDIASEENLWDLYQKWRSHHTVSRDLTEKQKRFNVFKANVMHI
HNVNKMDRPYKLKLNKFADMTNHEFRNFYSSKIKHFRMLHGPRPTTGFMHDKADNLPASVDWRKKGA
VTGVKDQGKCGSCWAFSTIVGVEGINKIKTGKLVSLSEQELVDCEKDNEGCNGGLMENAYEFIKKNGGI
TTERIYPYKASDSRCDSLKRNSPVVNIDGHEMVPEKDEDALMKAVANQPISVAIDASGSDLQFYSEGVFT
GNCGTELDHGVAVVGYGSTHDGTKYWIVKNSWGTEWGEQGYFRMQRGIDAEEGLCGITMEASYPVKL
SPDNPKPAPSKDEL

>Solyc08g005180.2.1

MAGDFGFSLRNEPTQILLPYIHNNRNGMHICYAYSTTEAVSALFAVDYNSTPVELSTQQIADQMPRSFNY

AQQGRKRNATLGCYFGSHVDALYYARNFGLYEATTYPKRNTSWDINFPDPLPNEVKYKIGEVVRVRTEN
IAAKWQRVGFEDLVTDEQINQVLRHQPMVGAIRVPWISKERYIDEATVENPTDVEDGAAVRIHSEAHSVLI
TGWGIKNGVEYYEVKNHGAMNGVIMAMLK

>Solyc11g008260.2.1

MAKGGGLTYALSVTILTCAFSLLPFHHTSAAAAVPEEFKIRQVTDGRNPTTTAHGGSNHHLLGTPAEHRF
KSFIQEYNKEYSTREEYVHRLGVFVKNLLRAAEHQALDPTAVHGVTQFSDLTSEEFERMYMGVKGGDR
TSLLREFGSHAPPMEVKDLPNSFDWREKGAVTDVKMQGSCGSCWAFSTTGSIEGANFIATGKLLNLSE
QQLVDCDNTCDKKDRKACDSGCRGGLMTNAYKYLIEAGGIEEEDSYPYTGKRGECKFSPDKVAVKVSN
FTNIPIDEQQIAAYLVNHGPLAVGLNAVFMQTYIGGVSCPLICGKRWVNHGVLLVGYGSKGFSILRLSNQP
YWIIKNSWGKRWGENGYYKLCRGHGMCGMNTMVSAVMTQTS

>Solyc12g056000.1.1

MALAFNWKFAFAALLVLVMQAYQATSRDLYEASIVKRHEQWMARFGRVYKDDAEKAKRFKIFKDNTEYI
DSVNMAGIKPYKLDVNEFADLTNDEFRVTRNGYRMPSHKKSPEITSFKYENVTAPATMDWRKKGAVTGI
KDQGQCGCCWAFSAVAATEGINKIKTGKLISLSEQELVDCDTSSDMGCEGGLMDDAFKFIIKNHGLTTES
NYPYEGTDSTCKTGKESNHAAKITSYEDVPANSESALLKAVANQPVSVAIDASGSDFQFYSSGVFTGEC
GTELDHGVTAVGYGEASDGTKYWLVKNSWGTSWGENGYIRMQRNVDTEEGLCGIAMEASYPTA

>Solyc12g056010.2.1

MAMTFNWKLAFIALLVLVMQASQATSRGLYETSMVQKHDQWMTRFGRVYKDDVEKAKRFKIFKDNTEYI
DSFNKAGTKPYKLDINEFADLTNEEFRATHNGYRMPSQKKSPEITSFKYENVTAPATMDWRKKGAVTGIK
DQGQCGCCWAFSAVAATEGINKIKTGKLISLSEQELVDCDTSSDMGCEGGLMDDAFKFIIKNHGLTTESN
YPYEGTDSTCKTGKESNHAAKITSYEDVPANSESDLLKAVANQPVSVAIDASASDFQFYSSGVFTGECG
TELDHGVTAVGYGKASDGTKYWLVKNSWGTSWGENGYIRMQRNVDTEEGLCGIAMEASYPTA

>Solyc12g056020.1.1

MAMTFDWKLAFVAALLVFGMQDFQATSRGLYETSMVQKHEQWMTRFGRVYKNDVEKAKRFKIFKDNID
YIDSFNKAGTKSYKLDINEFADLTNDEFRATHNGYKMPFQKKSPEITSFKYENVTAPATMDWRLKGAVTG
IKNQGQCGCCWAFSAVAATEGINKIKTGKLISLSEQELVDCDTSSDMGCEGGLMDDAFKFIIKNHGLTTE
SNYPYEGTDSTCKTGKESNHAAKITSYEDVPANSESSLLKAVANQPVSVAIDASGSDFQFYSSGVFTGE
CGTELDHGVTAVGYGEASDGTKYWLVKNSWGTSWGENGYIRMQRNVDAEEGLCGIAMEASYPTA

>Solyc12g088670.2.1 C14

MAAHSSTLTISILLMLIFSTLSSASDMSIISYDETHIHRRTDDEVSALYESWLIEHGKSYNALGEKDKRFQIF
KDNLRYIDEQNSVPNQSYKLGLTKFADLTNEEYRSIYLGTKSSGDRKKLSKNKSDRYLPKVGDSLPESID
WREKGVLVGVKDQGSCGSCWAFSAVAAMESINAIVTGNLISLSEQELVDCDRSYNEGCDGGLMDYAFE
FVIKNGGIDTEEDYPYKERNGVCDQYRKNAKVVKIDSYEDVPVNNEKALQKAVAHQPVSIALEAGGRDF
QHYKSGIFTGKCGTAVDHGVVIAGYGTENGMDYWIVRNSWGANWGENGYLRVQRNVASSSGLCGLAI
EPSYPVKTGPNPPKPAPSPPSPVKPPTECDEYSQCAVGTTCCCILQFRRSCFSWGCCPLEGATCCEDH
YSCCPHDYPICNVRQGTCSMSKGNPLGVKAMKRILAQPIGAFGNGGKKSSS

>Solyc12g094700.3.1

MSRATFARDFSIVGYSPDDLNSIDNIINLFETWMERHNKIYKSIEEKLHRFEIFRDNLKHINERNNIVSNYSL
GLNDFADLSHDEFKKMYLGLKVQNERRSNDEFIYSDFVDLPKSVDWRKKGAVTDVKNQGSCGSCWAF
STVAAVEGINQIRTGNLTSLSEQELIDCDTKYNSGCNGGLMDYAFQFIVSQGGLHKEDDYPYLMEEGTC
DEKRDESEVVTIDGYHDVPVNDEQSLLKALANQPLSVAIEASGRDFQFYKGGVFDGHCGTALDHGVAAV
GYGSTKGLDYIIVKNSWGAKWGEKGYIKMKRNTGKASGLCGINKMASFPTKNK

>PITG_00245

MAITDKSDLPLLQPRISPLLDVNSVSQSSTSPLQSIWENFHADRFKLRSIGSVLMWIALGSFVSSAVLAPK
KLVDYTHNGVSMHAVQDQLDLMAACPPDGKGEGDVDVVQTNKGAGWFLGGNDPEHVLSPLPEVTSL
DDFPERWDWRDYNKTGISLTTSVMNQMVPRACGSCWAFATVSALSDRIRIARFKTTGRLDTEVLLSPQV
LLDCGMRSFGSCHGGDPRYAHKWIHENGIVDLTCNPYIASHPSWMGRGDCAATQCHTCNLKGECFVL
EDPIKYRISEYGTLNFATSEEFQLQAMNEIYHRGPIVVSMYSLSPEYRQYKGGYILRDSTKYPGTTHVVSL
VGWGTDVKTGVKYWIVRNSDGTNWGDRGFFLAERGVNIYNMESHGAWAVPIV

>PITG_00395

MKGREHLHPKVHSLPTMSLDEAQELEQLPKHLDWCERGFCVPSWNQHIPQYCGSCFAHGAMSSAQD
RIKIANTRRKYTGADVMLGRQSFLNCAPGHGLSAGCDGGEASDVYEFMRLYGLPDESCLPYNATDHTK
YQATNGTCPPEGYCMNCMYTPESKKVPQCFPVTKMVRYRAKSHGHLSGELAMMKEIMEDGPITCGIAC
SEEFTYKYKAGILEDKTGFMDIDHDVEIVGWGEEDGVKYWHIRNSWGTYWGMNGFFKIVRGKNNLGIE
ADCAFMRPDISDEELVWEEKAVYGGSIFGIVPFKKSAKDHPIKDTSEDVTRPDGEVLAAHYTESFATETE
AHHQDRSFATLAVTFFVSGCVCAALAAVIVLKFRGHRYVYRTIA

>PITG_00588

MGKKKSKTTLPSEDHEASASESLLSSSDVRVHHLERHESAVIRRQRSKSRRIWCAIIALTTGILGFIAVLAI
LRAVEDASHLAPTKRPVFPTQYEASVTFHMPYMDMVEPLYVHVDETKGLQKLSYYGGTDVYIYNTSGTS
YQIIPVIRERKCFKSGSESLQHIFPNMTLFEPQHGVFLVEGRPCFSWKFVTKLHEPTEDGLLGEYTLYVD
QKTERPVRFHYVGRNGMLGGSHIDEYFLDYIYVREGPVDEDVFSFLPSSMNCTEMPGDDGGPSRIPKQ
DIHMLMPEGSTVKKEIFDNFSATHEKEYNDDAEAVQRMATFHHNLRFINGENRKGLPYHLEVNKFADLS
YEERRALHRPSRVKRAKDNKAMAVHELSTFEDPGDVDWRTKGAVTPVKDQGACGSCWTFGTTGALE
GALFAQQKKLYNMSQQNLLDCSWDYGNNACNGGLDYQAYEWIIANGGLETTATYGAYRNAPDYCHFN
VDNAIGRMKGFVNVTSVQALNDALATIGPLSVSIDANLPSFYFYGGGYYDDVECKSDLDSLDHSVLAVG
VTTHNDQKYTLVKNSWSTHWGEDGYIKISQKNNLCGVATAATYPVLAD

>PITG_02423

MRASLIVALAAATASASPLSLPELTSSGGYGYVRSPDRSVSLTSPRPHDYIDVSKLPKNFDWRNVNGTRY
VSISRNQHIPHYCGSCWSFAATSALADRILIFKERNPGNKPSVEVHRGVVLSPQVILNCDKKDNGCHGG
DQLEAYRYIKEHGVPEEGCQRYAATGHDTGNTCTDMDVCENCLPSKGCFPQKSYDKYYVSEVGTTLGE
QQMMAEIYARGPIACSVAVTDGFLKYSGGIFDDKTNATDVDHAISIVGWGEENGVPFWVLRNSWGSFW
GESGWMRLVRGVNNVGVEGECAFGVPRDDGWPTPTKIEEKEEDKVKEPQEETSVESTLGGCRQKLHF
AGGERVISPLPHETMDVTDLPKSWDWRDVNGKNYVTWDKNQHIPKYCGSCWAQGTTSALSDRISILRN
ASWPEIALSPQVLINCHAGGTCNGGNPGLVYEYAHRHGIPDQTCQAYQAKNLQCDQFAICETCWPSKE
SFTPGVCEPIKKFAKYYVSEYGSVSGAERMKAEIYKRGPIGCGVHATSKFESYTGGIYSEHVMFPLINHEI
SVAGWGYDEETDTEYWIGRNSWGTYWGENGWFRIQMHHNNLGIEQDCDWGVPLPDGSKPNDFVVD
YQGNEAETMVVLHSSDDQCHLFTDVFGRLLQLLLGCIAIFLLYIKRKLEFPIRPIKVWAMDVSKQSLGAFYI
HCISVILSIVMIAASTENYDEVRK

>PITG_02474

MLPVTVLSMGALARFTPSGSSQVMYGQAIQVVNTIGGTAAGSFTSSSKVGASGSASSFNDVSVGSLES
NETITFPPSSSSVGSAGSSTTVTTITESEEQQRFDQALADIAELQKLHPHATFSINSPFALLTSDEFLSYVN
RFGIDPDSNPVKNSTSSTTGMFTMDAEDSGTTTPSSIGSDIMTSAAAAGETVDWQEAGCVTAVKDQGE
CGACWAFSATAAMESGYCVATGSLPSLSDQQLISCHTEDGNRGCGGGYAAYSLDWIANERRGKMCTL
DTYPFTSENGNVAGCSMNSCTEFNVGVTGYESVREDPGAIEDAVRKQPVSIFLYSGSTAFQYYSGGVLT
GANCDKTGSHSGLAVGFGETEDNILYWRIKNQWGTSWGEDGYVRVQRRFSGDSEGACGVELYATWPT
FDVSATPTSSPTPTTSAPSGTAATPSVTTATPPITTMAPSTNPVTDTPSATNAVVDQAASASAASGSDSTI
TKVGVDQMTSATSGSTAHYETVKTDPVGGSIASTATPAATAIV

>PITG_03020 Pain2

MRIASTSLLLASLALADALKTPLEYEHEFSAWMKTHSVSFSDALEFAKRLENYIANDMYIMEHNLENAWT
GVKLDHNEFSSMSFEEFKFKMTGYVMPEGYLEQRLASRVDNLWSDVQVPDSVDWQDKGGVTPVKNQ
GMCGSCWAFSTTGAVEGAAFVSSGKLVSLSEQELVDCDHNGDMGCNGGLMDHAFAWIEDNGGICSED
DYEYKAKAQVCRDCEKVVKISGFQDVNPQDEHALKVAVAQQPVSVAIEADQKAFQFYKSGVFNLTCGTR
LDHGVLAVGYGSENGQKFWKVKNSWGSSWGEKGYIRLAREENGPAGQCGIASVPSYPFATLIKKDEET
ETQKIVEEPRSVPAANAVESFPAEEARDFRPVNLADLFSSAKIKQCGDVGSAIIDFSDLEVTPSSPQRGQ
PVSFFGNGNAKKDFDSANFKLGVKLAGTQVFGHSGKLCGDTHIPLPLGLGHIDVHGFACPMKKGKSSD
LKVDVNLPIIAPAGNYEIQLTSDDDSNSSLFCVNVELDLTGGETAKKTHVYEPISYM

>PITG_03414

MAPTWTTLLVLMAATVAPLITQAQDPSSFGTLQSCDDARCLWADRDGVAVSSDTMVTQFLQDEGMDAG
PSEFRRRMEDHVDYLEQVQKHAAGRDWAFSYAMGVNSRHLYHDGSRSLSPADFVEQEHQASQRQQR
RRLTEQRRLAIRETLDWCSKDNSHNQSICTDIKSQNQCGSCWAFAAADAIETAVVVNAGTSPRSLSPQQ
FLECSSREMTATFDYCWADGGVDGSPWLLTKMIWGSRNNACSGGMTHAAFADAAQLHWSLLSQLDLP
YNEEDTSQASAATLANACDNSSSDNAAASISGWEQVAGPSCDLSSDSTELLKLVLQQQPISVAINSGGS
FDAYKGGIYTCPNDGDFASSGDINHAVVLVGYGSDGSTDYWILKNSYGASWGEKGFLRLAMDSKINCGL
SVFPVIPTGAIAGAAHTAVDGGGEVEFVGMSPDSWIVCGIAVAVVTLFLTVIGVIYASRQRNAFKETL

>PITG_03415

MQIRAITFVISTALVNGAAVQTTDRSVGTLVDCPSVRSQDSPCLWAGENGQVVDSRSLRELFIERNYVAY
SDKESYGRNLQEHMTYIEDVSMYARQVGHDFSYHMGVNDRHLTSSSTRKLTPEQFVDQELTSANSRRL
QEANSTNNSTISNSGSSEYWNWCDKDNSVGHSVCSPVKSQKSCGSCWSFVAADAIETAVVITENASAA
VSLSPQQFLTCSSLQTTQTFEYCWASDSGVAGASWMQTEIKWESQNDGCNGGMTHGAFMDAAQNG
WGLVTELTMPYDDSNSGSSSATNLSSSCSVSADQAAASITGWEQIVGGDCTASKNCTLLLRSALEKQPI
AVAITSNGGFGEYAGGFYNCPNNGEMASKNDLNHALLLVGYGTDSSVGDYWILKNSYGSSWGDSGFM
KLVADAKINCGLNVFPVIPTGAKAGAQASTTVDSGGDKIFVGLSPTAWIGVAAATTIFTLVTTAIGIGVSQR
KLKVIRKQNSAMYAARTPTNTQNGF

>PITG_03416

MRVQATTEHSLGTLVDCPAFRSADSQCLWAGENGEVVDSKTLRDLLIRRNLVSFSERESLTRNLQAHMT
YIEDVHMYANSVGHDFSYHMGVNERHLTSSARRRLSPQQLVDQEVKSAYSRRLKVEASGSTASATSTV
SSSGSSEYWNWCDSDNSFGYSVCSSVKSQQNCGSCWAFAAADAIETAVVIAENASAAVSLSPQQFLTC
STLETTQTFEYCWASDSGVDGASWMETEIKWESQNNGCNGGMTHGAFIDAAQNGWSLVTELTMPYD

DSSSSSTSSSNVSSACSVSDNKTAASITGWEQVVGTDCTASNNCGTLLRTALEKQPIAVAINSEDPFGEY
AGGFYSCPNDGDLSSKDDVNHALVLVGYGTDASVGDYWILKNSYGSSWGASGFMKLVADSKVNCGLNI
FPVIPTGASAGAAASTSVDSGGDKVFVGLSPSTWIVVAAVTTIFTIVMTAIGMLFSQRRLKAMRKQNSAM
YATRPAANAEMAAH

>PITG_06926

MHSGIDYQRYLKEIDTAQADLDEWRSKFGDVAQKNGWMPVSEARSTDDQEEDLRQRIFLTKQSIAKVQ
AANPNANFSIMSPFSAMTDEEFNKYVVNSYVSGNSTQNDNRTSARHLRSAGSSDLTFDALSDSVDWST
SKCMAPIQSQGSCGSCWAFATVSAVESAQCIASGKELFVKYSEQQLVSCNTKNWGCYGGSPFYAFDYV
QQNGLCSEESYPYTFGGGYTRSCSRSCAAQDTGLTGYAKVSGEDDLLRALDEHPVIVAVASANSVWKQ
YTGGVVSSCDSWQPDHAVVAVGYDSSSIKIRNSWGPEWGEGGYIRLARSSSKAGTCGVLIDMSTPQM

>PITG_06927

MHAGINYERYLTEIDNIKAETDEWKAMFEKTCKENNWMPEYSTEERSSVDQDEDLRQRIFMSKQDVLE
AQASNPNAHFSIMTPFSALTKEEFASKVLNSYRSLRQEGTYTFTSMQDMINSLMQSLQQQMGGSWSTY
APVTPAPVVEPLVKKSSTPVRTEKAILKTANSVDWSASKCMSPVQSQGQCGSCWAFASVAAVESLQCIK
NGQSGINKYSEQQLVGCDSKNMGCGGGAPVYAYEYIQKNGLCSESALPYTSSNGGAVSCSASCSKSQ
TGITGYERINEGDEAGLVEALKSQPVVVAVASGNAAWKQYTGGIMSTCETTQVDHAVLVVGYDDNTFKV
RNSWGENWGEAGYVRMARSSSGMGTCGMLTDMSRPKM

>PITG_06928

MHAGLDYHRYLAERAETRQELADWKANFGEMAQNNGWMPPSSGNSEERSTDDEDEDHLQRFYMTK
QNISAIQALNPNANFSVNTPFTLLTNDEFAAYVGKSYRAYNASSVSTTRRLRSWHRSSNSETTYSSSSPA
KTTTTSTSSRTNSNPMLSGAANGGTTYDATNVKTVTSTGADGKSTTTTTTTTSSGPGRESTTVSTSSGS
TPSNFGFGSDFSSLWQQWGNGFNFGGMGRNDFQPETVKPAGSNSISTITDAPSTPLPTTAAPTPTPTE
TTVTPSPPKTSTSPATVTATDTSSTSSRVDWADSSCMSPIQSQGSCGDCWAFSTAAAIESGQCIHGGQK
TLQKYSEQYVQQNGLCTENDYPFTSSDGTAASCSTGCSAVDTGIKGYKTVDDASGLASAVAQQPVIIAV
ASGNNAWKQYTGGVISSCDTSELDHAVVVVGYTDSEWKIRNSWGDSWGEEGYIRLERTSDTTGTCGM
YGDMSYPTF

>PITG_08784

MNTALLLALAALVATADAAAPAVPTDRMEQFLEEKAVLHEELNEWKQSDAGLYAMEHGFVPTASARNVS
ATTDEELRRFFLSKLLVEDAQAANPEAVFSTDTPFTLLTHDEFAKFIGESYQRDSGPLKATSIADALPLNLT

AASTEKDWTTSGCVAPVKNQGDCGSCWAFASVAALESAICLSGQPLTLLSEQQVVDCDKVSYACDRG
WPSTALDYAKQSGGICTQEAYPYVSGDLGYHQTCKSTCTRQKVTIRKVVAVPRSDAGLVQAIETQPVAV
GVAADNPTWKQYKSGVVSSCSTSQLDHAVLAVGYSPSFFKIKSSWCPQWGENGFMRLKRGSGTSSSG
TCGIIGSLSVYP

>PITG_12041 Pain1

MKVFGFLFLAATAAFAPAKALTTDLPSSLTASEQKTWEAFVDYALDYEKSYRNDANDHDVVQLRFRSFAT
NLERIQTHNEAYERGEHSFTLGLNDLADLADAEYKQLLSYRTRDSKSSSASETFVKPENVEDLPATWDW
REHSTVTPVKNQGQCGSCWAFSAVAAMECAYALSTGTLESLSEQELVDCTLNGIDTCNHGGEMSEGYE
EIITNHKGKIDREEVYRYTAESKGVCNAKDDKAIGHFTSYANVTSGDEAALQAAIATKGVQAVAIDASSFT
FQLYRHGVYSWPLCGNAPDALDHGVAAAGYGVYKKKDYWLVKNSWGNSWGMKGYIMMSRNKDNQC
GIATDATYPIMTKEEVVEDRPIVLETTELASIM

>PITG_12916

MQTVVGYELRLHHAQTRTRRRALLGPTRSSDETRAEALANTERVVDEANAEHAAGQKSFFMGYNELS
DLTDEQYRAFLTSRPDRDSPRRKQRKKEPNRRKKGKQVTISFEIDDGSSESDDEEEDIEVPIELDWTTK
DGGKYMTPIKNQGTCGSCWAFAGVAAVESRYAIENNVQASPLSVEQVLSCSADLDHIRSKFEDNMTSS
SEGCAGGMPFLTYMYLQLAQPHGISCESAYPYVMATNETHPQCSSSLTSQVAVAWKSNGSAYKLVAAS
EKALLKAVTSGPVTANIDATGSGFRHYAGGIYDAKDCLSDGDEVNHAVVVVGFGETEAGEKFWVIRNTW
GTMWGEDGYMRIARGSHVGSYGPCNLYVYADYPVNLTVGSNATSGEPSCPVAASKFESLPLMKLVGLS
GNQIVMLVLCTVLTVLAGVTLYHGTEFIQNRKEAAGELRYQDSYARWILPSRDQIAAALAQRRQRPATEQ

>PITG_13074

MKFPLVLEGSCHGFTTKETCMKNHCAWCVCAAVPSSCYSPEEADQLPPAIFQCDKGQHLQSWDLTTVP
STSLELNSLFEAWKGLHGKSYDSPIQNELRRGIFEVNARSVAAHNSKNHKSFVMELNEFADTTWDEFQS
WYLGAPQECSATETTDVVYGEVPVQKDWRADGAVSPVKNQGKCGSCWTFSTTGCLESHVKLKHGEF
TILSEQNLLDCAQNFDNHGCNGGLPSHAFEYIKYNGGLDTEETYPYEAKEGKCKFNTYHVGVQVDQVV
NITTRNENELRAAVGSTGPVSIAFQVVSDFRFYESGVYESKECRSDEKDVNHAVLAVGYGVEDGKDHWI
VKNSWGSQWGMDGFFQIARGSNMCGVAVCASYPVVV

>PITG_16276

MLLSICSGSRTSALIKVFGFLFLAATAAFAPAKALTTDLPSSPTASEQKTWEAFVDYALDYEKSYRNDAND
HDVVQLRFRAFATNLERIQTHNEAYDSKSSSASETFVKPENVEDLPATWDWHEHSTATPVKNQGQCGS

CWAFSAVAAMECAYALSTGTLESLSEQELVDCTLNGIDTCNHGGEMSEGYEEIIINHKGKIDREEDYGYT
AESKGVCNAKDDKAIGHFTSYANVTSDDEAALQAAIATKGVQAVAIDDSSFTFQLYRHGVYSWPLCGNA
PDALDHGVAAAGYGVYKKKDYWLVKNSWGNSWGMKGYIMMSRNKDNQCGIATDATYPIMTKEEVVED
RPIVLETTKLASIM

>PITG_17314

MNTALLLLLASVAATDAAVPLVPTDRMAQFLQEKSGLESELNTWKQSDAGQYAKDHGFLPIPTSRDVGG
QADEELRRFFLTKLMIQEAQVTNPEAIFSTDTPFTLMTDQEFVNTDKDWTTSECVVPVKNQGQCGSCW
AFAAVAALESAICLSGQPLTPLSEQQVVDCDEASYACQGGFPGDALTFIQQSGGVCTEEAYPYVSGDSG
DRDTCKSSCTREAVTIRKVVGVPESDAGLVQAINTQPVAVGVAAGNPTWKQYKSGIVSSCTTSELDHAV
LAVGYSPSYFKIKNSWSTQWGEEGYMRLKRGAGTSSAGTCGIIGPKSVYPQL

>PITG_20589

MLGGSHIDEYFLDYIYVREGPVDEDVFSFLPSSMNCTEMPGDDGGPSRIPKQDIHMLMPEGSTVKKEIF
DNFSATHEKEYNDDAEAVQRMATFHHNLRFINGENRKGLPYHLEVNKFADLSYEERRALHRPSRVKRA
KDNKAMAVHELSTFEDPGDVDWRTKGAVTPVKDQGACGSCWTFGTTGALEGALFAQQKKLYNMSQQ
NLLDCSWDYGNNACNGGLDYQAYEWIIANGGLETTATYGAYRNAPDYCHFNVDNAIGRMKGFVNVTSV
QALNDALATIGPLSVSIDANLPSFYFYGGGYYDDVECKSDLDSLDHSVLAVGVTTHNDQKYTLVKNSWS
THWGEDGYIKISQKNNLCGVATAATYPVLAD

>PITG_22022

MLPVTVLSMGALARFTPSGSSQVMYGQAVQVVNTIGGTAAGSFTSSSKVGASGSASSFNDVSVGSLES
NETITFPPSSSSVGSAGSSTTVTTITESEEQQRFDQALADIAELQKLHPHATFSINSPFALLTSDEFLSYVN
RFGIDPDSNPVKNSTSSTAGMFTMDAEDSGTTTPSSIGSDIMTSAAAAGETVDWQEAGCVTAVKDQGE
CGACWAFSATAAMESGYCVATGSLPSLSDQQLISCHTEDGNRGCGGGYAAYSLDWIANERRGKMCTL
DTYPLTSGYESVREDPGAIEDAYYSGGVLTGANCDKTGSHSGLAVGFGETEDNILYWRIKNQWGTSWG
EDGYVRVQRRFSGDSEGACGVELYATWPTFDVSATPTSSPTPTTSAPSGTAATPSVTTATPPITTMAPST
NPVTDTPSATNAVLPAQVPLRDLTRLSRRLALTK

>PPTG_18745

MRILRVFLLFCALHDAAAVPMQVVEDFELRLHHAQTRTRRRALLGPTRDSEATRAEALADTERVVDEGN
AEHAAGKKSYFMGYNGLSDLTDEQYRAFLTSRPDRDSPRRKQRKKQPTMNRRKRGKQVTISFDLDDG
SSESDDDEEDIEVPTELDWTTKEGGKYMTPIKNQGTCGSCWAFAGVAAVESRYAIENDVQASPLSVEQ

VLSCSADLDHIRSKFEDNMTSSSEGCAGGMPFLTYAYLQLAQPHGISCGSAYPYVMATNETHPQCSSSL
TADVAVTWKSSVSDYKVVAASEKALLRAVTSGPVTANIDATGDGFRHYAGGIYDAKDCLSDGDEVNHAV
VVVGFGETDAGEKFWIIRNTWGTMWGEDGYMRIARGSGVGNYGPCNLYVYADYPVNLTAGSNVTSGE
PSCPVPVLKFESLSLMKLIGLSGNQIAMLVLCIVLTVIAGVALYHGTEFIQNRKEAAGQLRYQDSYGRWILP
SRDQIAAALAQRNSQRPTTGQ

>PPTG_03239 PpPain1

MKVFTSLLLAATAAFAPASALTTDLPSSLSASEQQTWEAFVDYALDYEKSYRYDTYDQDLVQQRFRAFAT
NLERIQMHNAAYERGEHSFTLGLNELADLTDAEYKQLLSYRASVSKASRASETFVKPDNIEDLPATWDW
REHNTVTPVKNQGQCGSCWAFSAVAAMECAYALSTGTLESFSEQELVDCTLNGIDTCNHGGEMSEGY
EEIINNHKGKIDREEDYEYTAESKGVCNAKDDKAIGHFTSYANVTSGDEAALQAAIATKGVQAVAIDASSF
TFQLYRHGVYSWPLCGNAPDALDHGVAAAGYGVYKKKDYWLVKNSWGDSWGMKGYIMMSRNKNNQ
CGIATDATYPIMTKEEEIVVDRPIVLETTEVASIM

>PPTG_05143

MRASLIVALVAATVSASPLSLLERTSSGGFGYVRSPDRSVSLTSPRPHDYIDVSKLPKNFDWRNINGTRY
VSISRNQHIPHYCGSCWSFAATSALADRILIAKERNPGNKPSVEVHREVVLSPQVLLNCDKKDNGCHGG
DQLEAYRYIKEHGVPEEGYQRYAATGHDTGNTCTDMDVCENCLPSKGCFPQKAYDKYYVSEVGTTLGE
QQMMAEIYARGPIACSVAVTDGFLKYSGGIFDDKTNATDVDHAISIVGWGEEDGVPYWVLRNSWGSFW
GEDGWMRVIRGVNNVGVEGECAFGVPRDDGWPTPTKIEEKEEEKLEEPQEETSVESTLGGCRQKLHF
AGGERVISPLPHETMDVQDLPKKWDWRDVNGKNYVTWDKNQHIPKYCGSCWAQGTTSALSDRISILR
NASWPEIALSPQVLINCHAGGTCNGGNPGLVYEYAHRHGIPDQTCQAYQAKNLQCDQFAICETCWPSK
ESFTPGVCEPIKKFAKYYVSEYGSVSGADRMKAEIYKRGPIGCGVHATSKFESYTGGIYSEHVMFPLINH
EISVAGWGYDEDTDTEYWIGRNSWGTYWGENGWFRIQMHHNNLGIEQDCDWGVPLPDGSKPNDFVV
DYQGNQATDRNFLYVGDAH

>PPTG_05196

MLPVSVLSLGALALSTHFFSPGVALSEDAQQIYSEWKQSSYYGQAVQVVNTIGGTAAGSFSSSSKTDVS
GSASSSGAVGVGSLESNETITFPPTSGSTGSSATTSSASGTTTTVSSITESEEQQRFNQALADIAELQKL
HPHANFSINSPFVLLTSDEFLSYVNRYAIDPDSNPVKASTSSTAGMFTMDAEGSNTMTLSSSGGEIMTSS
AAAGETVDWQEAGCVTAVKDQGECGACWAFSATAAMESGYCVATGSLPSLSDQQLISCNTEDGNSGC
GGGYAAYSMDWVANERSGKMCTLDTYPFTSENGNVASCSMSSCTEFDVGVTGYESVHKDPGAIEDAV
RNQPVSIFLYSGSTAFQYYSGGVLTGENCDKTGSHSGLAVGFGETDDNILYRRIKNQWGTSWGEEGYV
RVQRRYSGDSEGACGVELYATWPTFDVSASPTPAPRPTTSAPSATTATPSVTTATPSATPLTTVMPSATP

VTNTPSAIETVVDQAASASTASSSDSTTQKIGVDQVASSASASSGSTSAYEAVNTDAGSGNAVDDSTAP
TKAPATPSVVQSDSVADDASYATVPSTDKRDCAM

>PPTG_10776

MWGGRFGSICKATLLATVLSTLLLNPVDAEEEGCRCKRTMKGREHLHPKVHPLPTMSLDEVQELEQLP
KHLDWCERGFCVPSWNQHIPQYCGSCFAHGAMSSAQDRIKIANTRRKYTGADVMLGRQSFLNCAPGH
GLSAGCDGGEASDVYEFMRLYGLPDESCLPYNATDHTKFHGTNGTCPPHGYCMNCMYTPESKKVPQC
FPVTKMVRYRAKSHGHLSGELAMMKEIMKDGPITCGVACSDEFTFKYKAGIIDDKTGFMDIDHDVEIVG
WGEEDGVKYWHVRNSWGTYWGMNGFFKIVRGKNNLGIEADCAYMMPDISDEELVWEEKSVYGGSIF
GIVPFKDSAKDHPIKDTSEDITSPEERLAAHYLPETESYATEPKTHHEDNSFAALAIMFFVSGCVCAALTAL
IVLKFRGHRYVYRTIP

>PPTG_11309

MNTALLLLLAAVAATDAAVPVVPTDRMVQFLQEKPDLESELNAWKQSDAGQYAQQNGFVPASSSRDVA
AAADEELRRFFMTKLMIEEAEATNPEAVFSTNTPFTLMTEDEFVKFIGESYQRGSGALAATSFVDAAPSN
STDPLSTDKDWTTSGCVVPVKNQGQCGSCWAFAAVAALESAICLAGQPLTPLSEQQVVDCDKTSYACQ
GGFPGDALAYIQQSGGACTEKAYPYVSGDSGDRDTCKSSCTREAVTIREVVAVPESDAGLVQAISTQPV
AVGVAAGNPTWKQYKGGIVSSCTSSELDHAVLAVGYSPSYFKIKNSWSTQWGEEGYMRLKRGAGTSS
SGTCGIIGPKSVYPQL

>PPTG_11311

MNTVLLLALAAIVTTADAAAPAVPTDRMDQFLQEGAALRSELNAWKQSEAGQYAKEHGFLPTPSSKNTT
DEELRRFFLSKLLVEDAQAANPDAVFSTDTPFTLLTHEEFAKFIGESYQRDSGALKATSFADEVPLNLTAS
PTDKDWTTSGCVAPVKNQGDCGSCWAFAAVAALESAICLSGKPLTLLSDQQVVDCDKTSYACDGGWP
STALTYIQQSGGVCTQEAYPYVSGDLGYHQTCKSSCSREPVNIRKVVAVPRTDAGLVQAISAQPVAVGVA
ADNPTWKQYKSGIVSYCSTTQLDHAVLAVGYSPSFFKIKNSWGTQWGEDGYMRLKRGAGTRSTGTCGI
IGPLSVYPQL

>PPTG_11313

MNTVLLLALAAIVTTADAAAPAVPTDRMDQFLQEGAALRSELNAWKQSEAGQYAKEHGFLPTPSSKNTT
DEELRRFFLSKLLVEDAQAANPDAVFSTDTPFTLLTHEEFAKFIGESYQRDSGALKATSFADEVPLNLTAS
PTDKDWTTSGCVAPVKNQGDCGSCWAFAAVAALESAICLSGKPLTLLSDQQVVDCDKTSYACDGGWP
STALTYIQQSGGVCTQEAYPYVSGDLGYHQTCKSSCSREPVNIRKVVAVPRTDAGLVQAISAQPVAVGVA

ADNPTWKQYKSGIVSYCSTTQLDHAVLAVGYSPSFFKIKNSWGTQWGEDGYMRLKRGAGTRSTGTCGI
IGPLSVYPQL

>PPTG_13332 PpPain2

MRIASSSLLLASLALADALKTPLEYEHEFSAWMKTHSISFSDALEFAKRLENYIANDMYILEHNLENAWTG
VKLGHNEFSHMSFDEFKFKMTGFEMPDGYLEQRLASRVDGLWTDVQVPESVDWQDKGGVTPVKNQG
MCGSCWAFSTTGAVEGAAYVSSGKLVSLSEQELVDCDHNGDMGCNGGLMDHAFAWIEDHGGICSEDD
YEYKAKAQVCRDCEKVVKVTGFQDVNPQDEHALKVAVSQQPVSVAIEADQKAFQFYKSGVFNLTCGTR
LDHGVLAVGYGSDNGQKFWKVKNSWGSSWGENGYIRLTREENGPAGQCGIASVPSYPFATLISKDEQT
ETEKVVEEPRSVPADNPVESFPAEERDFRPMNLADLFSSAKITQCGDVGSAIIDFSDLEVTPSSPQRGQ
PVSFFGNGNAKRDFASANFKLGVKLAGTQVFGHSGKLCGDTHVPLPLGLGHIDVHGFACPMKKGKFSD
LKVDVNLPIIAPAGNYEIQLTSDDDSNSPLFCVNVELDLTSGEGAAKKTHVYEPISYM

>PPTG_14796

MQIRAITFVLSTALVNGAAVQTTGRSQGTLVDCPTVRSAESPCLWAGENGQVVDSRSLRELFIERNYVAY
SDRESYERNLQEHMTYIEDVNMYAKEVGHEFSYHMGVNERHLTSSSNRKLTPEQFVDQELISAHSRRL
QEANTTNSSTISSSGSSEYWNWCDKDNSVGHSVCSPVKSQKSCGSCWSFVAADAIETAVVIAENASAA
VSLSPQQFLTCSTLQTTQTFEYCWASDSGVNGASWMLTEIKWESQNDGCNGGMTHGAFMDAAQNGW
GLVTELTMPYDDSNSGSSSATNSSASCTVSADQAAASITGWEQIVGGDCTASSNCTLLLRSALEKQPIAV
AITSNGGFGEYAGGFYNCPNNGEIASKNDLNHALLLVGYGTDSTVGDYWILKNSYGSSWGDSGFMKLV
ADSNINCGLNVFPVIPTGAKAGAQASTAVDSGGDKIFVGLSPTGWIGVAAATTIFTLVTTAIGIGVSQRKLK
VIRKQNSAMYAAQTPTNAQNGY

>PPTG_14797

MVPAPWLTLLALTAAAAAPSMTQAQDPSTFGTLLSCDDARCLWADRDGIAVPSDTMVTQFLQDEGMDA
GRSEFRRRMEARVDYLEQVQKHAADRDWAFSYTMGVNSRHLYHDGSRMLSPADFVQQEHRAAQRQ
QQRRLTEQRRLTNSTLDVRETLDWCSTDNPQNQSICTDVKSQNQCGSCWAFAAADAIETAVVVNTGTS
PRSLSPQQFLECSSREMTATFDYCWADEGVDGSSWLLTKMIWGSRNNACNGGMTHAAFADAAQLHW
SLLSQLDLPYNEEDTSQASAATLANACDNSSTDNAAASIAGWEQVAGPSCDESSDSTELLKLALQQQPI
SVAINSGGSFDAYKGGIYTCPNDGDFASSGDINHAIVLVGYGSDGTTDYWILKNSYGASWGEKGFLRLA
MDSKINCGLSVFAVIPTGAIAGAAHTAVDGGGAVEFVGMSPDSWIVCGIAVAVVTLFLTVIGVIYASRQRNA
FKETL

>PPTG_16596

MQPRVSTATVALAVAFVAAIISDNVKARPMHAEIVPKYHRYLADKETIKAELNEWLKTYSDSGPKHGYIPV
TESRSSDDDLEDKLQRFYLTKEQIEEARELNPMAEFSTDGPFTLMTMDEFKQFLSNSHLNETGKTEPPA
VEKPKTLSEKEKKTRPSEEVKNKEDNDTKQSKSDDKNDDEEKKTWAPATEGPVVVPMVRRLRNAGKS
QYAFNGGDYSNTGEGFQSTSDTYVKSSQETYESQSAPQPNTNNGGNTNQGWNFGDVSTGDSAQIAIV
SGGTGSGNNGNWWGTNGWGTTWGGNTGNTWSWNQQWTQPTGSNNQWTPPSNNQWTPAPNNQ
WTPPPNNQWTPPPNNQPPSANNPPAPAPVTPQTPPTLAPAPPPAPPAPAPPAPAPPAPAPPAPATKRPA
TKRPATKAPPTKAPATKAPAPAPAPAPVTKQKATTTASTSDTARDSDSDSVDWSKSGCVNPPGLQGQCG
SCWAFASLGALEAAQCIANGDKKAPSYSEQHLVSCDTKDFGCNGGAPVYAMQYLRDNGVCTESSYPY
TSVEGGTAAACVKTCTPVKSGITDIAQLKSGDESALLGALKKQPVVVSVISNNPMWKQYKSGVITSCNTA
TVDHAVLAVGYDATTIKIKNSWGTEWGEDGYVRISRSSQGMGTCAVLTDMSYPKV

>PPTG_16597

MPSQDRSTTVSVLRFIGGVAIAAVAVSGVDGRQMHAGLDYHRYLAERAETRQELADWKANFGEMAKNN
GWMPPPTSNSEERSADDDEEDHLQRFYMTKQNISAIQALNPNANFSVNTPFTLLTNDEFAAYVGKAYRT
YNGSSVPTNRRLRSWHRSSSTKTTYNSGTPGAISKITSTSTSSGTNSNTQASVLGGTVNGGTNYETTS
VKTITTTGADGKSTTTTTTTTTSSGPGTESTTVTTSSGSASTMSNFGFGSGFSSLWQQWGNGFNFGGM
GRNDFHPETVKPAGSASSSPTTNAPSTPTPITAAPTPTPTQTTATPSATAPTKSFTTTVTATDTSTASTSN
EVDWTDSSCMSPIQNQGSCGDCWAFSTAAAIESGQCINGGQKTLNKYSEQQLTSCDSQNYGCSGGAP
IYAMEYVQQNGLCTEDDYPFTSSDGTAASCSNGCSAVDTGITGYETVDDASGLATAVAQQPVIIAVASGN
NAWKQYTGGVISSCDTSELDHAVVVVGYTDSEWKIRNSWGDSWGEEGYIRLERTSDSTGTCGMYGD
MSYPIF

>PPTG_16598

MKPITLITTLVFATSMLAVVPVNARPMHAGINYQRYLTEIDTIKAETDEWKAMFEKTCKENNWMPEYSTEE
RSSVDQDEDLRQRIFMSKQDVLEAQAGNPNANFSIMTPFSALTKEEFAAKVLNSYVRGNTTRQTPTPSP
APPKRSLRQQETYTFTSMQDMINSLMQSLQQQMGGSWSIATVKPATDNTDTSTAKQWHWTIPATFPTV
APTPVAPAPAPNTPPPAPVTPAPAPVTPAPTPAPTPAPTPAPTPAPTPAPIPVTAKPKTAAPRTYAPVTPAP
VVEPLTKKPSTPVRTEKAVLNSANSVDWSATKCMSPVQSQGQCGSCWAFASVAAVESLQCIKNGQSEI
NKYSEQQLVGCDSQNMGCGGGAPVYAYEYIQKNGLCSESAYPYTSSNGGGVSCSASCSKSQTGITGY
ERINEGDEAGLVDALKSQPVVVAVASGNAAWKQYTGGVMTTCETTQVDHAVLVVGYDDNTFKVRNSW
GENWGEAGYVRMARSSSGMGTCGMLTDMSRPKM

>PPTG_16600

MHVPTLCLVIAFTILTIPTIARPMHSGIDYQRYLEEIDTTQADLDEWRSKFGDVAQKNGWMPVSEARSAD
DQEEDLRQRIFLTKQSIAKVQAANPSANFSIMSPFSAMTDEEFNKYVLNSYVRGNSTQSTNRTNSRQLR
SAGNSDSNIDAGSDNASIIKTFESLIKTFFNGLNTATIQPTSDSSANVKRSGSQASYKFSDFWDWRPLQP
TTNAPATTSSPTVAEASTDSVDWSTSKCMAPIQSQGSCGSCWAFATVSAVESAQCIASGKQSLTKYSEQ
QLVSCNTQNWGCDGGSPVYAFDYVQQNGLCKEDSYPYTSKGGYADSCSRSCDAQDTGLTGYSQVTG
EDELLNALDKHPVIVAVASGNNVWKQYTGGVVSSCDSWQPDHAVVAVGYDSSSLKIRNSWGTYWGEE
GYIRLARSSSREGTCGVMMDMSTPQM

>PPTG_08165

MGKKKSKTTLPSDDLEASASESLLPSSDAEYHRLEKHESAVLRRQRSKSRRIWCAIIALTTGILGFIAVLAI
LRAVEDASHLSPTKRPMFPTQYEASVNFNMPYMNMVEPLYVHVDEAKGLQKLSYYGGTDVYIFNTSGT
SYQIIPVIRERKCFKFGSESLQHIFPNMTLFEPQHGVFLVEGRPCFSWKFVTKLHEPTQDGLLGEYTLYV
DQKTERPVRFHYVGRNGMLGGSHIDEYYLDYIYVREGPVDEDIFSFLPASMNCTERPGDDGGPSRNPK
QDIHMLLPEGSTVKKELFDNFSASHDKEYNDDAEAVQRMATFHHNLRFINGENRKGLPYHLEVNKFADL
SHEERLALHRPSRVKRAKDNGAMAVHELTSFEDPGDMDWRTKGAVTPVKDQGACGSCWTFGTTGAL
EGALFAQQKKLYNMSQQNLLDCSWDYGNNACDGGLDYQAYEWIMANGGLETTATYGAYRNAPDYCHF
NVDNAIGRMKGFVNVTSVQALNDALATIGPLSVSIDASLPSFYFYGGGYYDDVECKSDLDSLDHSVLAV
GVTTHNGQKYTLVKNSWSTHWGEDGYIKITQKDNLCGVATAATYPVLAD

>PPTG_10928

MARTDELKASNVEVEVSKQHSSWQAAGNNSRFVCQSIGSMLFWVAVGGAITSMLLTTQKEQNSNNLLP
STKIQTNKGSGWYLGGDDPEHVLSSLPEIRSLDELPENWDWRDYNGTGISLTTSVLNQMVPRACGSCW
AFATISALSDRIRIAKFKKTGRLDAEVILSPQVLLDCGMRSFGSCRGGDPRYAHKWIYENGIVDSTCSPYI
AAHPSWIGSGECAATQCHTCTMNGDCFVVENPTKYYISEYGTLNFTTSEEFQLQAMNEIYHRGPIVVSL
YSLTPEFKHYTGGYILRDFEKCPGTTHVVSIVGWGKDAKTGVKFWAVRNSMGTHWGENGYFLIERGNN
TYNIENKGAWAVPIV

>PPTG_10929

MAIIDKSDLPLLQPRTSTLLNTNNVSEQSLTSPLQSIRESFHADRFKLRSIGSVLGWIALGSFVSSAVLAPK
QLGDYTHNGMSMHAVQHQLDLMAACTSTGSAKGDGDVEEVQTNKGVGWFLGGNDPEHVLTSLPEVT
SFDNLPENWDWRDYNGTGVSLTTSVMNQMVPRACGSCWAFATVSALSDRIRIARFRKTGRLDTEVLLS
PQVLLDCGMRSFGSCHGGDPRYAHKWIHENGIVDLTCNPYIASHPSWMGGGDCAATQCHTCNLKGEC
FVLEDPVKYRISEYGTLNFTTPEEFQLQAMSEIYHRGPIVVSMYSLSPEYRQFKGGYILRDSTKYPGTTH

VVSLVGWGTDAKTGVKYWIVRNSDGTNWGDRGFFLAERGVNIYNMESHGAWAVPIV

>PPTG_12287

MELNEFADTTWDEFQSWYLGAPQQCSATERNGVVYGEVPVEKDWRADGAVSPVKNQGKCGSCWTF
STTGCLESHVKLKHGEFTILSEQNLLDCAQNFDNHGCNGGLPSHAFEYVKYNGGLDTEETYPYEAKEG
KCKFNTYHVGAQVDQVVNITARNENELKAAVGSTGPVSIAFQVVSDFRFYKSGVYESKECHSGEKDVN
HAVLAVGYGVEDGKDHWIVKNSWGTKWGMDGFFQIARGSNMCGLADCASYPIVV

>PPTG_14795

MRVQALIALSTAVASASAARTDRSLGAFVDCPSVRSDGSPCLWAGQNGEIVDSKTLRDLLVQRNLVSFS
ERDGLTRNLQAHMTYIEDVHMYAKSVGHEFSYHMGVNERHLTSSPKRRLSPQGLVDQEVKSAHSRRLK
AEASGSTASTTSTVSSSGSSEYWNWCDTDNSFGYSVCSSVKSQQNCGSCWAFAAADAIETAVVIAENA
SAAVSLSPQQFLTCSTLETTQTFDYCWASDSGVDGASWMETEIKWESQNNGCNGGMTHGAFIDAAQN
GWGLVTELTMPYDDSSSGSTSSNNVSSTCTVSDNETAASITGWEQVVGTDCTASNNCTTLLRTALEKQ
PIAVAINSEDPFGEYAGGFYSCPNDGDLSSKDDVNHALVLVGYGTDASVGDYWILKNSYGSSWGASGF
MKLVADSKVNCGLNIFPVIPTGASAGAAATTSVDSGGDKVFVGLSPTNWIVVAAVTTIFTIVMTAIGMIISQ
RKLKSIRKQNSVMYAGRAPVNAQQTMAH

>G2342

MHAGIDYQRYLEEKDSAQADLDEWRTAFGDVAQKNGWMPVSEDRSADDQEEDLRQRIFLTKQNIASV
QAANPDANFSIMSPFSAMTNDEFTTYVMNSYIRDNSTSSSGAATRQLRSDTGSGFGKQPQERKFRRHL
TTYQFSNIWDWRPHQKPTPTPTTSARATTSSPIVTEKATVSSGSVDWSTTKCMAPIQSQGSCGSCWAF
ATVSVVESAQCIANGKTSLTKYSEQQLVSCNSQNWGCGGGAPEYALESVQQNGLCTEDSYPYTSDVGY
AYSCSSSCNAHDTGLSGYSQLSGEDELLSTIDEHPVVVAVASGNNVWKQYTGGVVSSCDSWELDHAVV
AVGYDSSSIKIRNSWGTYWGENGYIRLARSSSSEGTCGVTSDMSTPRM

>G2343

MHANINYERYLAERDEADDDLKSWKQQFFATSTKNNWMPDFSEERSLDDIDEDLRQRIFMSKQDVLEA
QASNPNANFSIMTPFSALTKEEFASKVLNSYVRANHTRTPPPPPRATTAPPATTTTKRSLRQQQEAYTFT
SMQDMINALMKSLQQQMGGTWTIGTVKPATDNVAKDASTSTNTHWVQPANNQWYKPATVAPAPATAAP
IPVTAAPVPVTQAPTPAPTPPPTPAPTPAPTPPTPAPTPAPIPVTAAPKTSAPKPQTRAPVVEPVVKKPST
PVRTEKATLSTANSVDWSASPCMSPVQSQGQCGSCWAFASVAAVESLQCIKGSRSGVNKYSEQQLVG
CDRQNMGCGGGAPVYAFEYIQQNGLCAESAYPYVSSNGNSPSCSASCAKSKTGITGYEHLKDGDEAG

LIEALKSQPVVTAVASGNAAWKQYTGGIMSSCQTTDLDHAVLIVGYDDTSFKVRNSWGADWGEAGYVR
MARSSSGTGTCGMLTDMSRPKM

>G2344

MQSRDRSTSARALRYIGVAVIAAAAVSGVSGRQMHAGIDYHRYLAERDVTKQELADWKSNFGDMAKQN
GWIPPSSSNSEERSMDDEEEDHLQRFFMTKQNISAIQALNPNANFSTNTPFTLLTNDEFAAYVGKAYRA
YNGSAVGSSSSASRRLRSWHRSSSTKTTYSRGTTPSIKTVTTSASSGSPTSVLGGTSNSDTSYSVKSVT
TTGADGKSTTTTTTTTSSGPGTESTTVTTSSGSAANAASNFGFSSGFSSLWQQWGNGFNFGGMGRND
FQPETVKPAGSDSSSTTPTPTPTTPTPTTPITAAPAAAAPTTTPTPTTAAPSATSSAKSSASTATVTATDTS
TTSSSNEVDWTDSSCMSPIQSQGSCGDCWAFSTAAAIESGQCINGGQKSLTKYSEQQLTSCDTQNYGC
NGGAPIYAMEYVQQNGLCTEDSYPYTSSDGTAASCSTGCSAVDTGITGYQTVDDASGLATAVAQQPVIV
AVASGNNAWKQYTGGVISSCDTTELDHAVVVVGYTDSEWKIRNSWGESWGEEGYIRLERTSDSTGTC
GMYGDMSYPTF

>G2345

MQPRVSTATVALAVAVMAAGLPDSSTLVDARPMHAAIVSKYHRYLEEKDEISAKLDAWLEVWGPKGPKN
GYIPVTESRSLDDELEDQRQRFYLTQEQISEAREANPMAEFGTDGPFTLMTMDEFKQFLSNTHVNEAEK
TEAPEIQKPKTMSEKQDDNSESEIKKSTSDGDDKKDADDKKKTFAPATEGPTVIAAVRRLRTSGKSQFAF
NGGDYSADAEKGYQSTTDSYTQTTQPMYQGNTGSNAGNTNANTNGGWNFGDVSTGDSAQVGTVST
GSNNQAWNWWGNNGWENNGTWWNTNNWWSGNTTWWNNWGGSNTGNSQWTPAPNSQWTPPLS
GWTPPSGSGNSQWIPAPASSDNQWSAGSSNNNQWTAPNTDETPAAQTPTTPVPTSVPETDPPAAPPV
TDPPATEAPVTAPPATDPPATKAPKPAAPAPAPAPVPATKKKSTTTDLKTSDTTTAGDSDSDSVDWSKSG
CVNPPGLQGQCGSCWAFVSIGALEAAQCIANGDKKAPTYSEQQLVSCDTKDYGCNGGAPVYAMEYLR
DNGVCTESSYPYTSMEGGTAAACAKTCTPTKSGITKIVQLKAGDEAALLSAVKTQPVIASVASNNVAWK
QYMGGVITSCDTATVDHAVLIVGYDATTIKVKNSWGTDWGEDGYVRISRSPKNMGTCAVLTDMSYPKL

>G2499

MHAGIDYQRYLAEKEATHAEWKDWEKKHGAKAIQLGLMPTSEERSSVDYEEDMRQRIFLSKQDVERAR
AANPHANFSIDVVYSIMTKEEFATKIMNSFAKGNSTITTRLPTPKPRNLRAQYNFTSSTNNEAVPLGESMA
AAKFPVTAMSATSATRSPSFGHHGSPAASTKKQSVSTKPTSKPITASNEAATVTQTVDWSRSACMSPIQ
NQGQCGDCWAFAATAAVESAQCIAGGQKSLHKYSEQQLVSCNTQNHGCNGGAPQYAFDYILKNGGFC
SETAFPYASSEGHVPSCARCNNANTGIKDYKVLDKGDEAGLIQAINQRPTVVTVAAGNEAWKQYTGGVL
SSCDTSALDHLVVAVGYDATSLKIRNSWGDNWGEDGYIRLKRKASGSGTCSVLQQMAYDDGIAAGKPL
CPSTFTPQSLWPGLPSLSVPSTLLVLATCGGASSRRLTRQCTCAAETSDSDNESDAGLSLADAGRTASA

PPSLRGATNEDDDQVATGDNENGNGSTGSTNGAGSHATWAKSLSLLELTKAVHDPLKASQALHNAEAL
RQAPALDNVFCSCYDVDTVLTTVQRRERGHFSPVQKLLLEPESIKQVFAYATSFSDEHHHLSENGVDGE
ATDPNSLKPHPDRYRQSYVATEIILRFYLKAMAWYGEPKQDPAEENGIKSANEEENPTKNGQPRATQDR
DSGSAPTVSLEGLAVSSSEAPTNMPTMVDFRSNASTRKMARIQRLRASMRFESSGSSISEFSVNGDED
EELLEDPATRGYLLRLEDLTANEWKRIFGGLFRFLWPLRIQNGGDNGENTVVDDEVEVDSVLAANMCRIT
KNFVTFPAVHRLICDENDDTEREWLLSRLAAHAYNPDIASLLHGLIHLSIRRGFEYFPIIRCLLVRIVEQVPT
RLTRSMSAVSSTSTVSSTSSASPRSSLGGSPPSVSPSSFFSSKTAPLSPSLFTTHTTSTVHARISGCAEI
MTKILKDEFPNTFRYYIQTKIQLASFESVETFERELFPPRMAPSDPAMHHKLKLAVLAALIENSTILARLAE
LGMAELRFLDAHHINGVCIPRVLVIDILRHAIEFSLHDSEQLEVFIAPVHLVLDTICASINYHQHLSTISEFAA
RDCFSDSDSDDDDTEDGEDFDSVAGVSGKDSPSKSVKRPGAVPARTTLYMGITPPALSYSPRSGKAVV
NHRPLASTLLVMHVVELLDDVIRMSNDRIDSRLSRLDLATSLIDIFEKFPKASILHCRLVKLYLNLLNRPTTN
GRVNNPLLRSVFRSPDSILEFILQKLHANSSGHIYDAHLAIIGVKIAKICSSPTLQQELIRQFCNNVKGWND
FASSLVASHYQQMDALDDSLLGLQVVTGGGRSGTPRKRNASEDEVDADFPLARPSSSASEYLSRELEP
FRRLPMEKEGFGSSHNLARGNEAVHPSDMFQSRSQSKFPESIIDILQSDESTSFDVEEDDFFVSGYAYQ
KRSKWAKVHLKFEKATCQLTLQDAAAAACGSPRNGASKATSPSKASMLKQFLLAHKQTWTSRPKKLVV
CNARKWIAFGRSLKKPNRGAFGFQVEVFDGHREEDETLTFVTRSDETRMRWFEAMQCAVTRTRTARY
SFSDIDEAANTELVECVAKNRGGSYLMVPDVNLLGPMTSGSFFIKSEVPEEMPFWGTYHGDQGIIKYVS
LFKQCLDVVSVEEKSIQAIGYSVIVEFDATFRRSESTADFDDSEPPAVKCACTDTYLISGNQIIGLTRTIADS
EKLLQLLCDDE

>G2500

MHAGIDYQRYLAEKEATHAEWKDWEKKHGAKAIQLGLMPTSEERSSVDYEEDMRQRIFLSKQDVERAR
AANPHANFSIDVVYSIMTKEEFATKIMNSFAKGNSTITTRLPTPKPRNLRAQYNFTSSTNNEAVPLGESMA
AAKFPVTAMSATSATRSPSFGHHGSPAASTKKQSVSTKPTSKPITASNEAATVTQTVDWSRSACMSPIQ
NQGQCGDCWAFAATAAVESAQCIAGGQKSLHKYSEQQLVSCNTQNHGCNGGAPQYAFDYILKNGGFC
SETAFPYASSEGHVPSCARCNNANTGIKDYKVLDKGDEAGLIQAINQRPTVVTVAAGNEAWKQYTGGVL
SSCDTSALDHLVVAVGYDATSLKIRNSWGDNWGEDGYIRLKRKASGSGTCSVLQQMVPLEV

>G2503

MHAGIDYQRYLTEKEATHAEWKDWEKKHGAAATKLGIMPTTEERSSDDYEEDMRQRIFLSKQDVKQAQ
AANPEAHFSIDVVYSIMTKEEFADKIRNSFAKGNTTIGHQPSRISRSTKKRSVSTPSTRTTNIITRSLNEAA
TMAQSVDWSRSACMSPIQNQGQCGDCWAFAAAAAVESAQCIAGGGRSLHKYSEQQLVSCNTKNHGC
NGGAPQYAFDYILKNGGLCSETAFPYASAKGYIPSCARCTKAYTGIKGYKVLNKGDEAGLIKAINKRPTVV
TVAAGNVAWKQYKGGVLSGCDTSALDHIVVAVGYDATSLKIRNSWGDNWGEDGYIRLKRRSSGAGTCS
VLQQMVPLEL

>G5836

MFVFVGRALVRVADTDLVPVCAIEAGILGQEVIKAISPKDEPICSYFCFDGVTCSRRRGTIGETNSSPGAL
WINRGPNMSTALATARAKTKGKRMLRNALDSYGDEDTSLSKWNARKKKAVEEGKWVWKESVANANAA
IKSRFDKEEVQSSLKARFVDVQAAMEEEMSSAIHVDQHGGQLLGPSNPQDNRCSWRERIGGKMHHCT
NLPPQEKKKKTDLQETPSTAPLAASKRFCLWHAKECRYDDHLMEKSRVIEIPNEFGMCLSCYEVTAGTL
RATLQKVPPRIGALKIPGVSETSARKELKREAMLKLPGAKSSLDGEAGRKLGPTSVCTWQKEHSEVNYV
WRCSNRVLMHPVLRGSYLPFCGFHAPRCIQEYGNKGKKEQACPLIDRKNRYGMCRNHLEAHLSTLSLE
ERGGVLLIDSDFDVPGIKECRKEEVVIPLVRHPLAPKYPPPPAFDPRDSPNLPMETTHAVVILPSRSPRSP
LDKIVKKDVIWRVQFLRRAEVVATRIQRIFRGNRARRRVRLLLYEQAAMDRMKACRVLQRFVRGFLGRR
RFEHEHENVHNAVPHIQRLLRGALERKHFRELLAAIRLQRNYRQYRQRLLARAFREEIAYMQALQRQAD
ANYLEMEKQMNTFRRLRARRVLRAHIVRWKRRQEMHEQEVAERLRNLLGTVKIQRQWRRYHRYMIIKK
RYGSAQMIQKRVRGWLTRHFWRGDPALHFVRNFVNPRSGFQYGKVVLEPQPSRSYSYPSWKIRAQYG
ALAIQRVFRGHLGRLEANECWAAMLKRWEWLGITPTDSSGQLSDTMTVGHQRYGFVLPSFAYHKDRR
QHMRPIANEPVPNRGHAYKYQCILDLISDRDGKRGWSLAKEEIYARQLKEEQEWLRAEEARRDAREAEI
ATKALRRHIASIRDPLEQSMPVSKAIFPLGCLVDVVGKVEGKTTLRRAKITAIHKEGGVNQRLSATFDVEYI
KPLRNSYGRLEESVEYRVDVARLRHIALVSTELTAKQNAGEVIQAAIDGLRREIELSKRNTSTTSVNGEGE
SAMTCVDAIAERLRDCREGHDLLYDQREFVDFVFRNVKLLKMKWLEVISQIRYGTRTIKEAAPATTQKVL
SMLHPEFGGDRAREEVAAIHPMPERAQVIEERMMKLGFEHDSKQADPYTDSSKPEKKEVATAETTRPE
TTPVQREAAPGPGSRSTVDDMMLFREDPTPQNLQDMQRLIYELKTIPREARREQIIHIASRQAHAYVCG
HPACGKCFSSRKVARLHQSKSHEGRERLASANPLVDQYLHSYWPQGAPWTEAEHQRMIGYFGCSHR
GCEKLQFRSQRELNRHHHREHGIDDDYESGSSCTTKSPLPALTEEVTTKRSTISRSAIWLGTYVVCRNL
EAKLGLAAATLTNPKACAVHDKPVQLCTACFLQQRRPAFPFRLYSAMAVRKQIDPGNGPLRIEAEIPLEAS
EDEEFMIFRDDDEEFCPAVNIWGVYSLGAPPKRKGKDKVILPDPLSSVLYLKVSTICRDAVGEAWMFGH
VLAHRKRAGPREAGREGDENEVVPDKARGLVFALLSQIVGSASIHYCSKNVFYRKYYSKPALAAPVFGP
PRSKLQCCSSFSLANLLTSSNTISPPPSFLLVTMKIALLLALAALVTATHAAVPAVPTDRMAEFLQEKAAIES
ELSQWKQSDAGAFAKQHGFMPSTSSKSAGGAEDEELRRFFLTKLMIEEAQATNPEAVFSTDTPFSLMT
EDEFVKFVGASYQRGSGLLTATASADVASNSTEAPSTEKDWTKSGCVAAVKNQGQCGSCWAFAAIASL
ESAVCISGGDLTTLSEQQVLDCDTASYACQGGFPGDALEFIQRSGGVCTEDAYPYVSGDSGDRDICQKT
SCKPEAVAIRKVVSVPESESGLIQAISGRPVAVGVAAGNPTWKQYKGGVVSSCTSTDLDHAVLAVGYGG
GSDGSTPYFKIKNSWGTQWGEAGFIRLKRGSGTGSSGTCGVIGPKSVYPQL

>G6772 PsPain2

MRIVGGLLLASLALADALKSPLEYEHEFSAWMSAHGVTFSDALEFARRLENYIANDMYILEHNAENAWT
GVKLGHNAFSHMSFDEFKFKMTGLVLPEGYLEQRLASRVDGLWSDVEVPSAVDWVDKGGVTPVKNQG
MCGSCWAFSTTGAVEGATFVSSGKLLSLSEQELVDCDHNGDMGCNGGLMDHAFQWIEDHGGICSEDD
YEYKAKAQVCRKCDSVVKVTGFQDVNPQDEHALKVAVAQQPVSVAIEADQKAFQFYKSGVFNLTCGTR
LDHGVLAVGYGNDNGQKFWKVKNSWGASWGEQGYIRLAREENGPAGQCGIASVPSYPFATLINKDEQ

ETEKVVEEPRSVPADKPVDSFPAEPERDFRPKNLADLYSSAKITQCGDVSSAIIDFDDLEVTPTSPQRGQ
PVSFFGNGNAKQDFSSANFKLGVKLAGTQVFGHSGKLCGDTHVPLPLGLGHIDVHGFACPMKKGKFSD
LKVDVNLPIIAPAGNYEIMLTSDDNSNSQLFCVNVELDLTDSDATKKAHVYEPLSYM

>G7168

MARSWTKWLALATSATAAQALDPSTFGTLLSCEGSRCLWADRDGVAVSSHAMVTQFLQDEGMNAGPS
EFRRRMEAHVDYLEQVEQHAAARGWAFSYAMGVNSRHLYHDGDRRLSPSDFVEQEHQAALRQRQRR
LTEQRRLANSTLDLRETLDWCSTDNTHNTSICTDVKSQNQCGSCWAFAAADSIETAVAVSAGTAPQSLS
PQQFLECSSREMTATFDYCWAEGGVDGSSWLQSKMIWGSKNNGCNGGMTHAAFADASQLHWSLLSQ
LDLPYNEEDTSQASAATLATACDNSSADDAAASISGWEQVVGTSCDISSDPTELLKLALQKQPISVAINS
GGSFDAYKGGVYTCPNDGEFASSGDIDHALVLVGYGSSGGTDYWILKNSYGSSWGEKGFVRLAMDSKI
NCGLSVFPVIPTGALAGAAHTVVDGGGAVEFVGLSPDNWIILGIAVAVATVFFTVIGVIYASRQRNAFKEM
L

>G7169

MRIHAAVLALFAAVAAAVQSTDRSLGALVDCPAVRSADSSCLWAGVNGDVVDSRTLRELLIEHNYVSYSD
REAYRRNLQEHMTYIEDVSMYAKNIGHEFSYHMGVNERHLTSSTKRKLTPEEFVDQEVFSAYSRRLQAT
TNSTVSSSGSSEYWNWCDSDNSVGHSVCSPVKSQKSCGSCWSFVAADAIETAVVIAENASAAVSLSPQ
QFLTCSTLQTTQTFEYCWASDSGVDGATWMQSEIKWESQNDGCNGGMTHGAFIDAAQNGWGLVTELT
MPYDDSSSGSSSASNTSSVCTVSADEAAASITGWEQIVGADCTTSSNCTTLLRSALEKQPIAVAITSNGG
FGEYAGGFYNCPNNGEMASKNDLNHALLLVGYGSDSSVGDYWILKNSYGSSWGDSGFIKLVADAKINC
GLNVFPVIPTGAKAGAQASTSVDSGGDKIFVGLSPAAWIAVAAVTTIFTLVTTAIGIGVSNRKLKTIREQNS
AMYAASNTPAAT

>G7170

MRVQASLFAFSAASAAASGVQTTERSLGALVNCPAVRSAASPCLWAGENGEVVASETLRELLVQRNLVS
FGDREPMHRNLQAHMTYIEDVFMYSKSVGHDFSYRMGVNERHLTSSTRRRLSPQQLVDQEVKSARSR
RLMTEASGSTATGTITVSSSGSSEYWNWCDTDNSFGYSVCSSVKSQQNCGSCWAFAAADAIETAVVIA
ENASAAVSLSPQQFLTCSTLETTQTFDYCWASDNGVDGATWMETEIKWESQNNGCNGGMTHGAFIDA
AQNGWGLVTELTMPYDDSGSSSTSSNNASSACSVSANETAASITGWEQVVGADCTASSNCTILLRSAL
EKQPIAVAINSEDPFGDYAGGFYSCPNNGDLSSKDDVNHALLLVGYGTDATEGDYWILKNSYGSSWGAS
GFMKLVADAKVNCGLNIFPVIPTGASAGAAASTSVDSGGDKVFVGLSPTAWIAVAAVTTIFTVVTTAIGML
VSNRKLKTIRKQNSAMYAARNQNNAQAAAR

>G8851

MQAVEQFELRLYHAQTRTQRRALLGPTRASDETRAEALANTARVVDEANAEHDAGKKSYFMGYNELSD
LTDEQYRAFLTSRPDRDSPRRKQRKKQPTRGAGGLKRGKQVTISFDLDASDGDDDEEEEEEDEDDIEV
PTSLDWTTKDGGKYMTPIKNQGTCGSCWAFAGVSVVESRYAIENDVQATALSVEQVLSCSASLDHIRSK
FPDNMTSSSEGCAGGMPFLTYTYLSLAKPHGLSCGSVIPYVMATNETDSACPSKLTDEVAVAWEQNVS
DYKVVATSEKALLRAVTRGPVTANIDATGDGFRYYAGGIYDAQDCLSDGEEVNHAVVVVGFGETDAGEK
YWTIRNTWGTMWGEDGYMRIARGGNVSEYGPCNLYLYADYPVNLTVGSNATSGEPSCVLPPSKFEALS
AMKLVGLSGNQIVMLVLCTVFSLAAGVALYHGSEFIQNRKEAAGELSYQDSYARWIMPSREQIAVALARR
NNQRQQRTNAAQ

>G9031

MKAPYLLLLAALSAAAASSPLAPDAGSCHGFTSKDTCVKNHCAWCECAAVPSSCYTPEEADQLPPAIFQ
CEKGALQQTLPWDLTSVPSTSAELRGLFAAWKSAHGKSYDSPAQDELRRGIFEVNARSVAAHNSKQSK
SFTMELNQFADTTWDEFQSWYLGAPQQCSATTEESGLEYGEVPTEKDWRADGAVSPVKNQGKCGSC
WTFSTTGCLESHLKLKHGQFKILSEQNLLDCAQAFDNHGCNGGLPSHAFEYVKYNGGLDTEETYPYEA
KEGKCKFNTYHVGAQVEQVVNITSRNEKELKAAVGSTGPVSIAFQVVSDFRFYKSGVYESTECHSGEK
DVNHAVLAVGYGVEDGKKHWIVKNSWGAEWGMDGFFQIARGSNMCGLADCASYPVVA

>G9362

MKRSFSFPLLIAALATLSAPSSAAPMCSRTWKQREHLHPKAHSLPTLTEAQVLELRALPKQLDWCERGM
CTPSWNQHIPQYCGSCFAHGALSAAQDRIKMMHRALGRVGPDVQLGRQTFLNCGPAHGLSDGCGGG
EPSDVFEFMHHYGLPDETCVPYSATDYRKYTDTNGTCPPEGYCMNCITTPDHPQGPHCFPVKTVVRYR
AKEYGRVAGEHAMMKELLKGPITCGIACSDEFTFNYSAGVFHDKTGFLDIDHDVEIVGWGEEADGTKFW
NVRNSWGTYWGMKGFFKIVRGVNNLGIESDCHWVNPDISDEELVFSQTTPLYGGSLWGIVPFAKGAAT
SHPINSTADVVLSNLPLQSEELPLETTTGLSENSEEIETVALRTAEEKEVEAAGVQNEGFSFSLFALFAALI
ALGTVISNVVSRFSRSEAYNRLI

>G12276

MRASLVLAFAAAAVASAAPFSLPVLTSAGGYGYVRSPDRSVELTSPRPHDVLDVAKLPKNFDWRNVNGT
NYVTISRNQHIPHYCGSCWSFAATSALADRIMIAKERSPSNKPSVEVHREVVLSPQVILNCDKKDNGCH
GGDQLEAYRYIKKNGVPEEGCQRYAATGHDTGNTCTDMDICENCLPSKGCFPQKTYDKYYVSEYGTTL
GEQQMMAEIYARGPIACSVAVTDGFLKYSGGIFDDKTNATETDHAISIVGWGEEDGVPFWVLRNSWGSF
WGEDGWMRLVRGVNNVGVEGECAFGVPKDDGWPTPTKIEEEEPVQEEEEKKDVVENTEEDTSVESK
LGGCRQKLHFAGGERVISPLPHETIDVKDLPKAWDWRDVNGRNFVTWDKNQHIPQYCGSCWAQGTTS

ALSDRISILRNASWPEIALSPQVLINCHAGGTCNGGNPGLVYEYAHRHGIPDQTCQAYQAKNLQCDQFAI
CETCWPSKESFTPGVCEPIKKFAKYYVSEYGSVSGADRMKAEIYKRGPIGCGVHATEKFEAYTGGIYSE
HVMFPLINHEISVAGWGYDEETDTEYWIGRNSWGTYWGENGWFRIQMHHNNLGIEQDCDWGVPLPD
GSKPDDFVITVDYEGNEEGQATARNFLHVSGGAN

>G12321

MLPVSALSLGVLALSAPVVVVSESSQQIYTEWKQSSYYNQAVQVVNTVGGTAAGSFTSAKNVASISGSG
SSAVDVSVGSLESNETMTFPPPTGSAGSSSSSVVTTITETEEQQRFDQALAAIAELQSLHPHANFSINTP
FALLTSEEFLAYVNRYAVDPASNPVKAGTSSTAGMFTADAGNSSSAITSSSSGDIRTSSAGSGETVDWQE
AGCVTAVKNQGECGACWAFSATAAMESGYCVATGGSLPSLSDQQLISCDGEDGNSGCGGGYAAYTMD
WVANERSGKMCTLDSYPFTSDDGNVPSCSMSSCTEFDVGVTGYDSVREDAGAIEDAVRKQPVSIFLYS
GSSAFQYYSGGVLTGENCDKTGSHSALAVGFGETDNNMLYWRIKNQWGTSWGEDGYVRVQRRYSGD
SEGACGVEMYATWPTFDVSASSAPSVTTPAPSVTTSAPSVTTPAPAVTTSAPSVTTATPTSTPSATHAAT
TPSPSVTKAATVTNTPSTTKTALNTEDHTEPVASSASGSDLVIQNETVDQVSASSGSTRLYEAVTSDAST
VEDASNYSTTPSLAQSDSVAGDASYASDTTPSPTTPSVVVNGAGASTPSTGKRDCVM

>G16966 PsPain1

MKVLSSLFLAAVALAPCHALTSDPPASVSASERQTWEAFVDYALDYEKSYRSDANDQALVQHRFRAFAT
NLQRIEAHNAAFERGEFSFTLGLNDLADLSDAEYKQLLSYRARDSKGASETFSVSPEDVKDLPDSWDW
RQHGAVTPVKNQGQCGSCWAFSAVAAMESAYQLSTGKLESFSEQELVDCTLGGVDDCSHGGEMSEG
YEEIIKHHGGKIDREEDYEYTAESRGVCNAKDDKAIGHFTAYANVTSGDEAALQAAITTKGVQAVAIDASS
FTFQLYRHGVYSWPLCGNAPDALDHGVAAAGYGVYKKKDFWLVKNSWGDSWGMKGYIMMSRNKDN
QCGIATDASYPIMTKEDVAMEPRPVVVEETTEVASIM

>G19311

MGITDKSDLPLLQPRTSTLLNSDNVSSTSTTSSTLLQSTWTNIRADRFKLRSIGSVIGWIAVGSFVSSVAF
APKQLTDFTNGGVSMHAVQGQLDLMAACTSTGSANGEGDVAEIQTNKGAGWFLGGNDPEHVVTSLPE
VASFDDLPENWDWRDYNGTGISLTTSVLNQMVPRACGSCWAFATISALSDRIRIARFKKTGRLDTEVLLS
PQVLLDCGMRSFGSCHGGDPRYAHKWIHENGIVDLTCNPYIASHPSWMGGGDCAATQCHTCNLKGEC
FVLEDPVKYRISEYGTLNFTTSEEFQLQAMNEIYHRGPIVVSMYSLSPEYRQFKGGYILRDSTKYPGTTH
VVSLVGWGTDAETGVKYWVVRNSDGTNWGDRGFFLAERGVNIYNMEGVSCRSLSVLGGISEDLLVST
SIGHQIGGNSHSELVDSLVRTNVVAAGSRLEAALRRVDRGDFVAPAFRDTTERYANRPLKIGTVATISTPQ
QHAQVLGLLEPHLQLGMTAVDVGCGSGILVAAMAHLVGPTGFVMGVDIVPELVEFSKENLQRSLGNEAA
DKQTKVIVSAGKKDLGLPEDGRYDCIHVGVAVETKAEAESFLEYLKPGGGLLIPLGGAGAEQKLVKMTKL

ADGTVDKRDVMSVLCQPMLDSIPVEVVRDTRAEKLARVEKALTQWREEFEYKNGRKPTRDDLMGNSE
SKKLFQEFAALRK

>G19312

MAQSRPGASLNDLELTEDEGQIPSTPQSNGPGSLTPEQQLLARFRLHKQHRKEKERRLLQTRLAAASR
ASRSAAAAAAAAGAPRSPSSRKQKPPKPGAVDLTRVDHNNHPHRWKSAEELLQEQERRAQQHQLTAA
APVRPYPGRLRGQALEEVPRQTAAPAAKGESADTAREDEGILQLDQSQFPLHLFDSDEFEAHTPQEWL
EKERVGASPYFFQGEWRWRSCAVLSYDTAKAQYLVQFQGSDRQKWVRRINLRFESESPVTFERRVAA
ARERREQVKAMLRFDDFLARQDASQLRAMGRLTLERVHARVVAGLPERVALLDSQPAAFLLRQLTDTAI
QEYMRSMKKAALLKQLRNDPELQVRFQGLDLIPGPTKPGAITYGKVEIPEHDFERNRRRVSTVSYTSSP
QLLAVLRRMYAGWEKTFQKLLLVQVDKSLATTASTENHREGVSVAANGAGASPTTTTLASQMPFRVLDF
QALQASHANKVAEILLVDWRRALVENVIDNLQDHFDLFLSDRVAYDASRLKRVLTGLELRLAAQLREVVH
RSVDEWVRFVRHHAERGSLVTTRPGTSMKPDDAQGDVAASSRTNGGDGNDEEEEDAPVRPSRKRFT
QMLDQKAIRAPHSSLFSVQLGFVNDEVVVEPSTQEVTAVLLEPLDAIVSAVQEIDRLDCDIMGLLSLDRRP
LLDFAVGHEHESERQKASRRAVVAECLDALQAAKVEVRDSVDHAMQAAHALAARFAAYTDFVHFDATAF
IAELQPLQLQEQQLLKEGKTPPTADEQAYLPQLCAQIRRFHELAFCVDVIAFDFVALPLVCVHTSALKKQL
RTRSLELRDTLISSLVRDARAQNLAITARYAAILARINEKPTNEAQLAKLKQFVGESKAVIAGIQREVAAIHI
RLDALNEFSHKLSAEDFTLAHSTKEWPLKVAHAADSCDSALEEDKVRMMDRLALEKEAFELDLEHFEG
DVQAFTRYGEVEHTDKYVELAITLYDALQDARAKALDFNAREAVFGFPPTEYTPLLGKLESDFAPYYKLW
TMSSEFHASRQAWLNGPFLELKGGTIEGLVTEWWKASYKLSKSLVDDAPGSAEVALILRERTEEFKAYL
PVIQSLASPALQERHWEKLRHTIGFEESEEELTLQLLLDRGITQHLETIQEIGTFAEKEYSLQKNLSAMIGE
WEKVEFQTAPYRETGTYLLRSTDDIVALLDDHLVKTQTMRGSPYIKSIEKDCKAWEKKLQYSQQLLDEW
MACQRTWLYLEAIFSSEDIMRQMPTEARRFASVDALWRKTMEDTVADPTFLTVIAMDKLLAKFQRANEK
LDEIQKGLNDYLEMKRLHFPRFFFLSNDELLEILSQTKEPRAVQPHLGKCFEGVFNVTFQNGPPLMITEM
RSAEGEVVPLRLPVSPESNKNKGNVEMWLLEVEQSQWDSVRDQTERSMAAYPLEDRETWMLKWPAQ
VVLAVSQVYWTQDVTRALNLGDGANGIKAYVEVLNSQLDKIVMLVRGNLTKLERTTIGALVVIDVHARDTI
SHMIEKDVESDQDFEWISQLRYYWAEGVKSAGVFDLQARIVNARVRYGYEYLGNTMRLVITPLTDRCYR
TMMGAVDLMYGGAPEGPAGTGKTETVKDLSKAIAIQCVVFNCSDGLDYLAMAKFFKGLAGCGSWCCF
DEFNRINIEVLSVIAQQILTINEGKKAGVDKFLFEGTFIKLNASANVFITMNPGYAGRAELPDNLKALFRPC
AMMVPDYALISEIRLYSFGFAQARSNARKLTQVLQLASEQLSSQKHYDYGMRAVNSILVATGALRQQLGN
DPFWTEDKIVLRSVQDVNLPKFTSDDLPLFRGITSDLFPDVLLPLPDHGALLRHIDETCVRGISIVPDVNIP
LECKPEFKMKVVQFYETVQVRHGLMIVGTTGSGKTCVVHSLATAMTSCYIEEIELQEENGTKGAPTLQQ
RVNIHTMNPKAITSGQLYGNFDENTHEWSDGVLACTYRNCARDTSPELQWVMFDGPVDAVWIENMNT
VLDDNKKLCLMSGEIVKMTDRMRMVFETEDLEEASPATVSRVGMVFLEAKVLGWEVLVRTWLNTRLPV
AFASHVEYLEESFRWIVPPMLYFVDKHCTVPTPVTFLEHAASLLRLFECALRDGFPDPPADGTTPAAASS
SVPTGVDSQRILECVLVKAVIWSIGACIDTKSRRIFDRYLRDFLSTELAVAVVTADMHLQDGEEGVDDELG
VPPSPSLLYFKDFVAKSPSYVLLPDRAALLAIPEEGLVYDYRFDTRRSVWVNWMEASGGGAFVIPRDAQ

FTQVLVPTIDSERNAWLLDTLIRHHFHVLCTGDTGTGKSVSIKKKLLSGLNDPPGASDSPPKFAPSIFLNF
SAQTSANQTQDLIEAKLDKRRKGVLGPPLGQSCVIFVDDLNMPAKETYGAQPPIELLRQWMGHGGWYN
RKDNSFTQLVDIQFIAAMGPPGGGRTRITQRYVRYFNLINFVPFDNDSLRAIFTRITDWFLLNFPQAVKQL
GGAVVSATIDIYNTISQALLPTPAKSHYTFNLRDLSKVFQGVAQASSDTIKDGKDFVRLWSHECLRVFSDR
LIDDKDRAWFADILSKTVKLHFDLQYASTDVRGPNATLIYGNFGGAGDSKNGSKNYAELRDREKLQNAM
QVFLEDYNNMSAAPMRLVLFQNAIEHVARISRVIHQPLGNALLVGVGGSGRKSLTTLAVFMAEYKLFQIEI
SKSYSRTEWRNDIKKVLQLSGLNNQPTVFLFSDTQIVEEAYLEDINGLLNTGEVANLWANDELVQMNEAL
EPAATASGVNAGNSAELYNFFVGRCRTNLHIVLALSPIGEAFRRRLRMFPSLVNCCTIDWFAEWPDEALR
SVADYFLVDIELPAQVKVGIVDVCVGMQESVSALTRDFLQSLRRYYYVTPTSYLELLNTFKKLLNNKRVEV
MTMKQRYDNGLTKLMETAEQVEKMQVELEALQPLLKVATIETDALLETISREQKEANATKDIVGAEEKLC
NEQAADANAIKESCEAELAEAIPALENAVKALQTLTKGDITEIKAMKKPPDGVKLVMEAVCIMMRVPPVKV
KDPAGGTKKVDDYWGPAQKTLLGDTRFLQNLLEYDKDNIPVEAMDKVRPYAANPDFQADKIRKASVAA
SGLCSWVHAMVVYDRVAKVVAPKREALKAATLALDKAQSELKVKQDALQVVLDKVARLEEDLAAAYKKK
SDLEFQVDDCSKKLTRATQLIGGLGGEKARWSDMSAQLQVVYDNVVGDIMLASGVIAYLGAFTSIYRER
AVDLWCTELTKQAITCSKTFTLTETLGEAVQIRAWTIAKLPNDSFSIDNAIMLQRSNRWPLMIDPQGQANR
WVKNMEESNNLKVVKQSQAGFVRMLENSIMIGAAVLIENMPEEIDPMLEPILLKQVVKTGGVATIRLGDN
TVEYDPNFRLYMTTKLRNPHYPPETCVKVNLLNFMATEEGLQDQMLGIVVAKEEPVLEQQREKLVLEDA
ANKKTLKEIEDQILYLLQTAKGNILDDERLIETLGASKITANKIEEKVREAAVTQQMIAEKRQGYLPVAFRAS
QLFFCIADLTVIDPMYQYALEWFINLFVFSISRAESSSVLATRLDNLNDAFTFILYQNVCRSLFEKDKLLFAF
LLAIKILVGNGTIDGGELRYFFTGNTQMDVQKPKPAGSEGWLNDKTWANIVGLDALPSFIEFSDAFATELS
LWEVSYNSTDPAETLTNISALATLDAFQRIIVLRCLRPDKVIPAVMSFVATQMGQRFIDPQPFDLKAGFDD
SNCSTPLIFVLTPGADPMSELLKLAAELGFNKKFVAISLGQGQGPLAENAIAEAIDNGTWVCLQNCHLSV
SWLPTLEKICEEITPDRVHASFRLWLTSEPTRAFPSYILQHGVKMTNEPPKGMRANLKGSYLTIDEGWVA
SCRRPREFKKLLFGLCFFHAVVRERTKFGPLGWNISYVFSSSDLAISKDQLKISLDDLQPDDPIPYAALAY
LAGECNYGGRVTDDKDRRCLITILSDFYTRDILSDSYTFSPSGLYYAPSADGSLSVFLNYIDQLPMNEGP
EVFGLHDNANISTAIAETNLLLESALSLQPRGASGGGGGAVKSWDEVLDETARDIAAKLPPLYDLEKAEL
AFPVSYSESMNTVLTQELGRFNRLLALLQISLVEIQKAIKGLVVMSAELEAMGNSMVNGHVPARWSAVAY
PSLKPLGSWVTDFLARLAFLQNWLTRGAAPPVYWISGFFFTQAFITGTQQNYARKHKLPIDQVGYDMVV
LAQPASELTTPAEDGAYVDGLFLEGARWDATTHTLAESKPRELYVPLPVLHLLPKARDQIEPIEDTDPKGT
AHVYLCPVYKTSKRQGTLSTTGHSTNFVMSVRLPMSAQHRQKHWIRRGVALLTQLDTAIELMERRSIPIL
TISRVRYRYPKSLFRNSQTMATTDNLRRKAATSQLVQKLVVWFAAGIAITSALLASRHTNDDPHSSTVTV
PTNKGSGWFLGGIDPEYVLTSLPEIHSLDDLPGHWDWRDYNGTGFSLTTSVLNQMVPRACGSCWAFAT
VSALSDRIRISKFKKTGRLDAEVLLSPQVLLDCGMRTFGSCRGGDPRYAHKWIHENGIVDSTCSSYVAS
HPSWIGGNDCAAAQCHTCTMNGDCFVVEDPVKYYISEYGTLNFTTSEEFQLQAMNEIYHRGPIVVSMY
SLTPEFKQYKGGYILRDSTKYPGTTHVVSLVGWGTDTGTGTKYWIVRNSIGTHWGDHGYFLVERGTNT
YNIENKGAWAVPIV

>G19464

MWGGSFCKATVLAMILLLHSSAGAEQDGCRCKRTMKGREHLHTLPTMSLAETQELEQLPKHLDWCER
GFCVPSWNQHIPQYCGSCFAHGAMSSAQDRIKILNKKRHYSVADVMLGRQSFLNCAPGHGLSDGCDG
GEAADVYEFMRRYGLPDESCLPYNATDHTKFQATNGTCPPQGYCMNCMYTPESKTVPQCFPVTKMVR
YRAKSHGHLSGELAMMKEIQRGGPITCGIACSDEFTYEYKAGILEDKTGFMDIDHDVEIVGWGEEDGVK
YWHVRNSWGTYWGMNGFFKIVRGKNNLGIEADCAYMEPDISDEELVWEEKSIYGGSIFGIVPFKETAKD
HPIKDTSEDVTRRDDEVPPAHYLPETNSFATEVESRHDDSSFILLAMMFFVSGCVCAALAAVLILKFRGH
RYVYRTIP

>G19648

MGKKKSKTTLPTEDMEASASESLLPASDAEYHRLEHHENAALRRQRTKSRRIWCAIIALTTGILSFIAVLAI
LRAVEDANHLAPTKRPVFPTQYEASVTFNMPYIDMVEPLYVHVDEVKGLQKLSYYGGTDVYIYNTSGAS
YQIIPVIRERKCFKSGSEPLQHIFPNMTLFEPQHGVFLVEGRPCFSWKFVTKLHEPTEDGLLGEYTLYVD
QKTERPVRFHYVGRNGMLGGSHIDEYFLDYIYVREGPVDENVFSSLPGSMNCTEMPGDDGGPSRNPK
EDIHLLMPEGTTRKKEIFDDFSSAHVKVYNDEAEAVDRMATFHHNRRFINAENRKGHSYHLEVNRFADLT
HDERLKLHRPSRVKRAKDNGAMAVHELSSFEDPGDMDWRTKGAVTPVKDQGACGSCWTFGTTGALE
GALFAQQKKLYNMSQQNLLDCSWDYGNNACDGGLDYQAYEWIMANGGLETTTTYGAYRNAPDYCHF
NANNAIGRMTGFVNVTSVEALNDALATIGPLSVSIDATLPSFYFYGGGYYDDVDCKSDLDSLDHSVLAVG
VTTHNGQKYTLVKNSWSTHWGEDGYVKISQKNNLCGVATAATYPVLAN
